# A rare TMEM175 mutation drives Parkinson’s disease pathology by disrupting proton gating and lysosomal acidification

**DOI:** 10.1172/JCI206733

**Published:** 2026-11-02

**Authors:** Xiaotong Yang, Ping Li, Man Lv, Lingyong Wang, Ruyi Shan, Zikai Jiang, Danzhen Chen, Fangqian Huang, Weijie Cai, Liding Zhao, Zuoyou Guo, Xinhui Chen, Zhidong Cen, Yichang Wang, Wei Yang, Xinran Li, Wei Luo, Lin Yang, Meiqin Hu, Haoxing Xu

**Affiliations:** 1 New Cornerstone Science Laboratory & Liangzhu Laboratory, the Second Affiliated Hospital & School of Basic Medical Sciences, Zhejiang University, Hangzhou, China.; 2 Department of Neurology, the Second Affiliated Hospital and; 3 Departments of Pharmacology and Pathology, Sir Run Shaw Hospital, Zhejiang University School of Medicine, Hangzhou, China.; 4 Department of Biophysics, the School of Basic Medical Sciences, Zhejiang University, Hangzhou, China.; 5 Zhejiang Key Laboratory of Pain Perception and Neuromodulation, Hangzhou, China.; 6 Institute of Fundamental and Transdisciplinary Research & The State Key Laboratory of Brain-Machine Intelligence, Zhejiang University, Hangzhou, China.; 7 Department of Molecular, Cellular, and Developmental Biology, University of Michigan, Ann Arbor, Michigan, USA.

**Keywords:** Aging, Cell biology, Development, Ion channels, Lysosomes, Parkinson disease

## Abstract

Transmembrane protein 175 (TMEM175), implicated in Parkinson’s disease (PD) risk, has been variably proposed to function as a background K^+^ “leak” channel or a proton-activated proton channel in lysosomes. Here, we identified the rare *TMEM175* variant p.L275R in a patient with early onset PD. Electrophysiological analyses revealed that TMEM175-L275R mutant proteins retain normal lysosomal expression and K^+^ “leak” activity, but specifically lose proton- and agonist-dependent activation gating. In skin fibroblasts isolated from the patient with PD harboring the monoallelic p.L275R mutation (*TMEM175*^+/L275R^), and in derived induced pluripotent stem cells and induced dopaminergic neurons, lysosomal H^+^ flux, acidification, and degradation were impaired. These lysosomal defects were corrected upon genome editing to produce the isogenic WT allele (+/+^Cor^), but appeared when the mutant allele was introduced onto a WT background (+/L275R^Mut^). Pathological α-synuclein aggregation was facilitated in patient-derived (+/L275R) and mutation-introduced (+/L275R^Mut^) neurons. Consistent with these genetic findings, pharmacological activation of TMEM175 with small-molecule agonists restored lysosomal functions and reduced α-synuclein aggregation in *TMEM175*^+/L275R^ neurons. In in-vivo studies, equivalent-site–mutation knock-in mice demonstrated synucleinopathy and PD-like motor phenotypes. Collectively, our work provides genetic and pharmacological evidence that severe loss-of-function mutations may contribute causally to the onset age and pathogenesis of PD and identifies lysosomal proton channels and transporters as potential therapeutic targets for PD intervention.

## Abstract 2

TMEM175 proton gating maintains lysosomal function; its loss causes Parkinson’s pathology, reversible by pharmacological activation.

## Graphical abstract

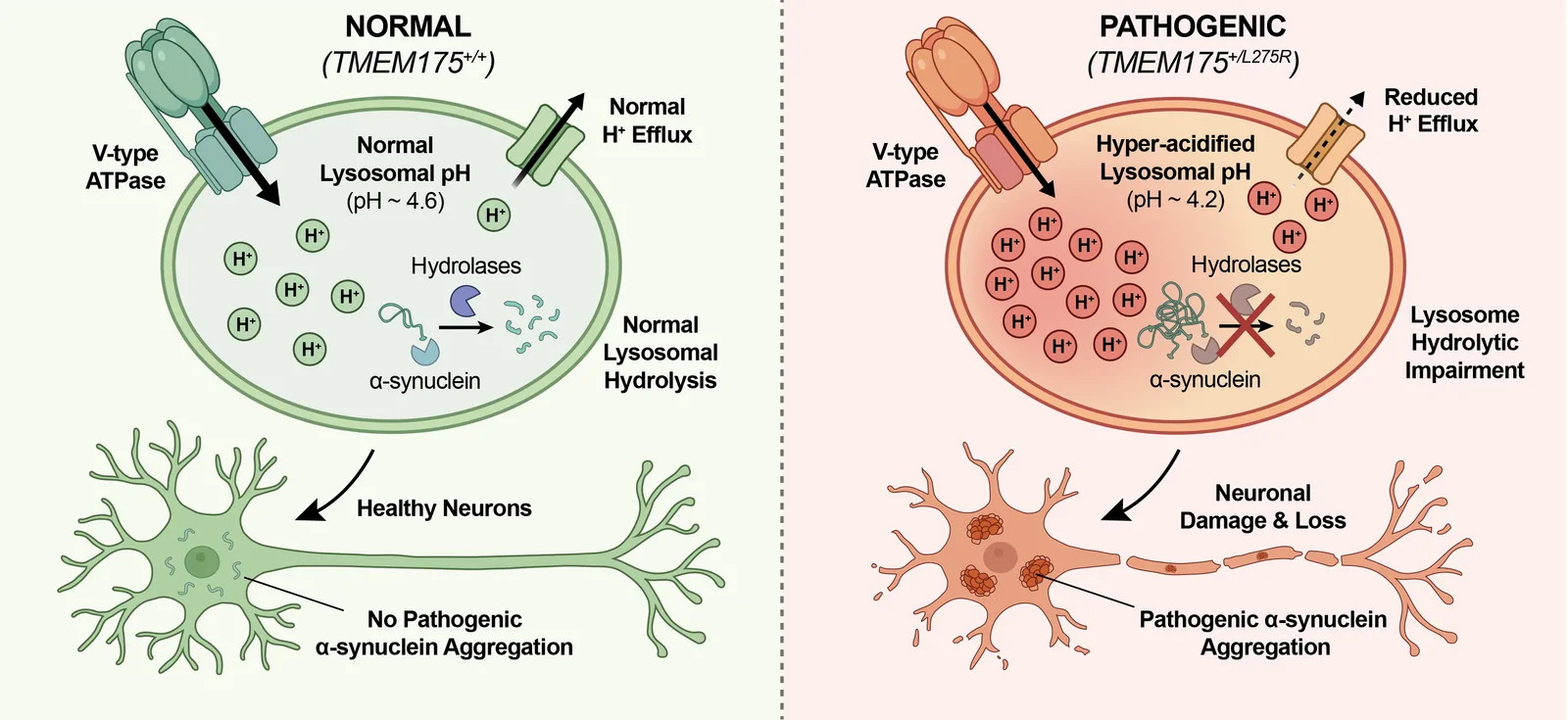

## Introduction

Parkinson’s disease (PD) is a common neurodegenerative disorder characterized by the progressive loss of dopaminergic neurons (1). A key pathological hallmark of PD is the intraneuronal accumulation and spreading of α-synuclein (encoded by *SNCA*) protein aggregates, which form Lewy bodies, predominantly in the substantia nigra (2). Emerging evidence suggests that lysosomes — cells’ recycling and degradation centers — are primarily responsible for the clearance of α-synuclein aggregates through pH-dependent luminal hydrolases such as cathepsin B (encoded by *CTSB*) and cathepsin D (3, 4). Deficiency of lysosomal glucocerebrosidases (GCases, encoded by *GBA*), which break down glucocerebrosides at acidic pH, contributes to α-synuclein aggregation and PD pathology (5). Recent genome-wide association studies (GWASs) suggest that as many as half of the known PD risk factors, such as *GBA* and *CTSB* variants, are autophagic or lysosomal genes (6, 7), suggesting that lysosome dysfunction is likely an elementary pathological mechanism in synucleinopathy and PD (8).

The maintenance of an optimal lysosomal pH (4.5–5.0) is essential for lysosome function, not only for hydrolase-mediated degradation, but also for lysosomal membrane trafficking, cargo delivery, and catabolite export (9, 10). Luminal acidity is established and controlled primarily by several H^+^-conducting lysosomal membrane proteins, including vacuolar-type (V)-ATPase and multiple H^+^ efflux channels and transporters (10–12). Transmembrane protein 175 (TMEM175) was recently identified as a lysosomal proton (H^+^) or K^+^ channel that regulates lysosomal pH balance and hydrolysis (11, 13, 14). Whereas TMEM175 deficiency accelerates α-synuclein aggregation in cultured neurons and mouse brains (11), GWASs have revealed a significant association of *TMEM175* p.M393T with PD risk in European cohorts (15, 16), but not in some cohorts of other ancestries (7). M393T, a common variant present in up to 20% of healthy individuals and patients with PD, is a relatively mild loss-of-function (LOF) trafficking mutation (11, 15). Whether rare but more severe LOF *TMEM175* variants exert greater risk, or even act as causative monogenic factors in PD pathogenesis, remains unclear.

In this study, we identified a rare LOF *TMEM175* p.L275R mutation in a patient with early onset PD from a local cohort comprising approximately 800 cases, all of whom were enrolled at the Second Affiliated Hospital, Zhejiang University School of Medicine (SAHZU). Heterologous overexpression studies revealed that the mutation abolished proton- and agonist-dependent channel activation gating without affecting lysosomal expression and other channel properties, including K^+^ and H^+^ permeability. As TMEM175 is a proton-activated proton-permeable channel, a proton gating deficiency is sufficient to diminish the lysosomal H^+^ “leakage” that is required to prevent lysosomal over acidification (11, 17, 18). Using patient-derived fibroblasts and induced pluripotent stem cells (iPSCs) and dopaminergic (iDA) neurons, we found that monoallelic expression of the mutant variant (+/L275R) was sufficient to cause profound defects in lysosomal acidification and degradation, leading to excessive neuronal aggregation of pathogenic α-synuclein. Genetic and pharmacological restoration of TMEM175 activity and lysosomal acidity in patient-derived cells fully rescued lysosome-dependent clearance of α-synuclein aggregates.

## Results

### Electrophysiological analyses identified a gating-deficiency TMEM175 mutation in a local PD cohort.

To investigate the role of TMEM175 in PD pathogenesis, we performed whole-genome/exon sequencing on more than 800 cases in a local PD cohort and identified several rare variants (minor allele frequency < 0.01) in the protein-coding region of *TMEM175* (Supplemental Table 1; supplemental material available online with this article; https://doi.org/10.1172/JCI206733DS1). As heterologously overexpressed TMEM175 proteins can ectopically traffic to the plasma membrane (11), we first studied these variants using whole-cell patch clamp in HEK293T cells transfected with WT and mutant TMEM175 (Figure 1A). Under this recording configuration, the extracellular side is topologically equivalent to the luminal lysosome side, such that the pH of the external/bath solution (pH_E/L_) can vary. At pH_E/L_ 4.6, which mimics the physiological acidity of the lysosomal lumen (11), large H^+^ currents (*I*_H_) with positive reversal potentials were seen in cells transfected with WT TMEM175, but not in nontransfected control cells (Figure 1, B and C). Among the mutant variants analyzed, a leucine–to–arginine (L-to-R) substitution (L275R) in the pore-helix resulted in the almost complete loss of *I*_H_, yet with normal protein expression (Figure 1, B and C, and Supplemental Figure 1, A–K). A dramatic difference in *I*_H_ between TMEM175^WT^ and TMEM175^L275R^ was also seen at pH_E/L_ 3.0 (Supplemental Figure 2, A and B). Nevertheless, although small, whole-cell *I*_H_ through TMEM175^L275R^ was significant, especially at pH_E/L_ 3.0 (Figure 1, B and C, and Supplemental Figure 2, A–D). Two other substitutions at the same residue — L275F and L275P — were present in public databases (GnomAD v4.1.0: 4-955871-C-T, 4-955872-T-C). TMEM175^L275P^ also showed a loss of H^+^ conductance (Supplemental Figure 2, E and F, and Supplemental Figure 1K). As TMEM175 is a proton-activated proton-permeable channel (11), the reduction in *I*_H_ could be caused by a deficit in H^+^ permeability and/or activation gating. However, 4-(2-Butyl-6,7-dichloro-2-cyclopentyl-indan-1-on-5-yl) oxobutyric acid (DCPIB) and arachidonic acid, chemical agonists of TMEM175 (11), failed to further stimulate H^+^ currents through TMEM175^L275R^ at pH_E/L_ 4.6 or 3.0 (Figure 1, D and E, Supplemental Figure 2, G and H). When we coexpressed mCherry-TMEM175^L275R^ with EGFP-TMEM175^WT^, we observed a roughly 50% reduction in whole-cell H^+^ current amplitude relative to cells coexpressing mCherry-TMEM175^WT^ and EGFP-TMEM175^WT^ (Supplemental Figure 2, I and J). When channel activity was assayed in the physiological setting of lysosomes using the whole-endolysosome [late endosomes and lysosomes (LEL)] patch-clamp technique (19), TMEM175^L275R^ failed to produce detectable whole-LEL H^+^ currents (Supplemental Figure 3, A–D), yet with normal lysosomal expression and trafficking (Supplemental Figure 3, E–G). Thus, L275R, as a mutation in the pore-helix (20, 21), may specifically abolish channel activation gating.

TMEM175 was initially characterized as a constitutively active (“leaky”) K^+^/Cs^+^-permeable channel (14). Luminal/extracellular K^+^ was recently reported to inhibit TMEM175’s H^+^ conductance, suggesting that K^+^, but not H^+^, is responsible for the lysosomal functions of TMEM175 (22). However, we found that extracellular/luminal K^+^ (1–10 mM) did not significantly inhibit whole-cell and whole-LEL TMEM175-mediated H^+^ currents (Supplemental Figure 4, A, D, and G–I). The rapid accumulation of local H^+^ on the cytosolic side during continuous whole-cell recordings of proton channels such as those of TMEM175 and otopetrins tends to quickly “erode” the transmembrane H^+^ gradient, resulting in the “artifactual” inhibition of H^+^ currents (17, 23) (Supplemental Figure 4, B–F). On the other hand, consistent with previous findings (11, 14), at neutral pH_E/L_, basal TMEM175-specific whole-cell K^+^/Cs^+^ currents could be detected (Figure 1, F and G). Remarkably, basal K^+^/Cs^+^ currents were comparable for TMEM175^WT^ and TMEM175^L275R^. However, DCPIB failed to further stimulate *I*_K/Cs_ in TMEM175^L275R^-transfected cells, in sharp contrast to the dramatic (up to 100-fold) activation seen with TMEM175^WT^ (Figure 1, F and G). Thus, the L275R mutation results in defective gating without compromising the channel’s cationic (H^+^/Cs^+^/K^+^) permeability/selectivity (Supplemental Figure 4J).

Endogenous TMEM175 generates only negligible amounts of H^+^ currents under the physiological setting of luminal pH (pH_L_ 4.6), potentially due to lysosome-associated membrane protein 1/2 (LAMP1/2) inhibition (24). Hence, pH_L_ 4.6 is proposed as the “threshold” pH for endogenous TMEM175 activation to prevent lysosomal over acidification (11). However, upon further acidification below pH_L_ 4.6 or in the presence of chemical agonists, TMEM175 may mediate profound H^+^ “leakage” activity to counteract V-ATPase–mediated acidification (10, 18). On the other hand, a high level of TMEM175 overexpression can produce significant H^+^ “leakage” even under physiological conditions (i.e., pH_L_ 4.6) (11). Although TMEM175^L275R^ retains its proton permeability, the lack of a proton activation effect may eliminate its H^+^ “leakage” activity in lysosomes. Indeed, lysosomal acidity, roughly estimated by LysoTracker staining, revealed that, unlike TMEM175^WT^, TMEM175^L275R^ overexpression failed to induce intralysosomal deacidification (Supplemental Figure 4K). Hence, the naturally occurring gating-deficiency L275R variant may be a unique tool for the differentiation of the controversial roles of constitutive K^+^ “leakage” and H^+^-gated H^+^ “leakage” in the regulation of lysosomal pH homeostasis, as well as PD pathology in patients carrying this variant.

### The monoallelic L275R mutation diminishes lysosomal H^+^ conductance in cells derived from patients with PD.

A patient carrying a monoallelic *TMEM175* p.L275R mutation (*TMEM175^+/L275R^*; case #37) was diagnosed with early onset PD in a local hospital at the age of 45 years based on clinical evaluations (Supplemental Figure 5A) and dopamine transporter positron emission tomography (DAT-PET) (25, 26) examination, which showed the bilateral loss of dopaminergic neurons, akin to other sporadic PD cases (Figure 2A). We collected skin fibroblasts from the patient (Figure 2, B–D) and generated iPSCs (Figure 2, E–G) and iDA neurons (Figure 2, H–K) using established protocols (27, 28). Successful neuronal differentiation was verified using immunostaining and functional assays (Figure 2I and Supplemental Figure 5, B and C). Whole-LEL recordings demonstrated that agonist-activated (DCPIB-activated) and proton-activated (pH_L_ 3.0–activated) lysosomal H^+^ currents were reduced by 40%–70% in *TMEM175^+/L275R^* cells relative to those in control *TMEM175^+/+^* cells derived from an age-matched healthy individual (Figure 2, B–K, and Supplemental Figure 5, D and E; Supplemental Figure 6). The nonphysiological pH_L_ of 3.0 was chosen as a “suprathreshold” stimulus to improve the experimental detection of endogenous *I*_H_ [see (11)]. Other lysosomal channel conductances, including TRPML1, did not differ between *TMEM175^+/+^* and *TMEM175^+/L275R^* fibroblasts (Supplemental Figure 5, F and G), nor was TMEM175 mRNA expression (Supplemental Figure 5H).

In a converse effort, we introduced a monoallelic L275R mutation (+/L275R^Mut^) into control (+/+) iPSCs using CRISPR/Cas9 technology (29). These +/L275R^Mut^ cells also exhibited greater than 50% reduction in *I*_H_ compared with the parental control cells (Figure 2, E–K). When we corrected the mutant allele in patient-derived +/L275R iPSCs using genome editing to generate isogenic “+/+” iPSCs (+/+^Cor^), *I*_H_ was fully restored (Figure 2, E–G, and Supplemental Figure 6, A and B). Similar results were seen with iDA neurons (Figure 2, H–K, and Supplemental Figure 6, C–J). Taken together, these results suggest that the monoallelic expression of the L275R variant plays a causative role in the observed *I*_H_ reduction.

### The monoallelic L275R mutation disrupts lysosomal acidification and degradation in human cells.

TMEM175 mediates fast H^+^ leakage in lysosomes, and its deficiency causes lysosomal hyperacidification in cells (refs. 11, 13–15, 24, 30; but see ref. 22 for a contrary view). We examined lysosomal acidity in *TMEM175*^+/L275R^ fibroblasts using 2 ratiometric lysosomal pH indicators: Oregon Green-488 dextran and CQ-Lyso (12, 31). When the fluorescence ratios were calibrated to pH values (Figure 3A), the average lysosomal pH in *TMEM175*^+/L275R^ fibroblasts was 4.18 ± 0.02, 0.38 units lower than that in *TMEM175*^+/+^ cells (pH_L_= 4.56 ± 0.02; Figure 3, B–D). An optimal lysosomal pH (4.5–5.0) is required for efficient lysosomal degradation, such that lysosomal hyperacidification (pH < 4.5) may counterintuitively decrease the activities of most lysosomal hydrolases (11, 12, 32, 33). The overall degradative capability, assayed by dye-quenched BSA (DQ-BSA) staining (34); the cathepsin B activity, assayed by Magic-Red staining (35); and the GCase activity, evaluated by PFB-FDGlu staining (36), were reduced in *TMEM175*^+/L275R^ fibroblasts compared with *TMEM175*^+/+^ cells (Figure 3, E–H, and Supplemental Figure 7, A and B). Consistent with these degradation defects, the accumulation of autofluorescence lipofuscin materials, often seen in fibroblasts affected by lysosomal storage disease (19, 37), was observed in the lysosomes of *TMEM175*^+/L275R^ fibroblasts (Supplemental Figure 7, C and D). When lysosomal hyperacidification was pharmacologically “corrected” using a low dose (1–3 nM) of the V-ATPase inhibitor bafilomycin-A1 (11) (Figure 3, B–D), lysosomal degradation was restored (Figure 3, E–H, and Supplemental Figure 7, A–D) without affecting lysosomal enzyme expression (Supplemental Figure 7E). Hence, lysosomal hyperacidity caused by reduced lysosomal H^+^ leakage is primarily responsible for degradation impairment in patient-derived (+/L275R) fibroblasts.

We also observed lysosomal hyperacidity and degradation impairment in +/L275R^Mut^ iPSCs and iDA neurons compared with their parental control cells (Figure 3, I–N). Discernible “rescue” effects were seen in genetically corrected isogenic WT (+/+^Cor^) iPSCs and iDA neurons (Figure 3, I–N). Thus, the monoallelic L275R mutation likely impairs lysosomal acidification and degradation across all 3 patient-derived (+/L275R) cell types.

### Pharmacological TMEM175 activation using potent small-molecule agonists rescues lysosome dysfunction in patient-derived cells.

Human TMEM175 proteins form homodimeric channels (20). Although the monoallelic L275R mutation causes haploinsufficiency or has a partial dominant-negative effect on lysosomal H^+^ conductance (Figure 2D), the expression of the single-copy WT allele resulted in intermediate-sized H^+^ currents that could be enhanced by chemical agonists. At pH_L_ 3.0, DCPIB upmodulated lysosomal H^+^ currents in *TMEM175^+/L275R^* fibroblasts, to a level comparable to those in control *TMEM175^+/+^* cells without agonist treatment (Figure 2D). We reasoned that an appropriate dose of DCPIB could be used to “counteract” lysosomal pH abnormality (Figure 4, A and B), restoring the lysosomal degradation capability in *TMEM175^+/L275R^* cells (Figure 4C and Supplemental Figure 7, A and B). Indeed, DCPIB treatment readily normalized the activities of cathepsin B and GCase in *TMEM175^+/L275R^* fibroblasts, but not in *Tmem175* KO mouse embryonic fibroblasts (MEFs; Supplemental Figure 7F). Remarkably, prolonged (> 2 days) DCPIB treatment also mitigated lipofuscin accumulation without boosting the expression of relevant hydrolases (Supplemental Figure 7, C–E).

To reduce potential off-target effects of DCPIB, we conducted several rounds of structure-activity relationship analysis to increase the compound potency by 10-fold from a half-maximal effective concentration of 11.7 μM for DCPIB [LysoHAgonist (LHA) 001] to 1.1 μM for LHA016 (Figure 4D and Supplemental Figure 8A). Solid-supported membrane–based (SSM-based) electrophysiology, used to measure ionic currents in native lysosomal membranes without LEL enlargement manipulation required for whole-LEL recordings (38, 39), confirmed LHA016’s specificity and potency for TMEM175 (Supplemental Figure 8B). The activation effect of LHA016 on TMEM175 was specific, as it evoked *I*_H_ in WT, but not *TMEM175*-KO cells (Supplemental Figure 8C). Lysosomal pH assays (11, 12) revealed that LHA016 at concentrations as low as 1–3 μM, which produced minimal inhibition on DCPIB-sensitive VRAC channels (11) (Supplemental Figure 8D), significantly abridged lysosomal hyperacidification in *TMEM175^+/L275R^* but not *TMEM175^–/–^* iNeurons (40) (Figure 4E). Consistently, LHA016’s rescue effect on lysosomal degradation was seen in *TMEM175^+/L275R^*, but not *TMEM175^–/–^*, iNeurons (Figure 4F), suggesting on-target specificity.

### Small-molecule agonists of TMEM175 prevent pathological α-synuclein aggregation in TMEM175^+/L275R^ neurons.

Lysosomal degradation, mediated by cathepsins B and D, is a primary route for the clearance of pathological α-synuclein aggregates (3, 4). To maximally facilitate α-synuclein aggregation in iPSC-derived neurons, doxycycline-inducible (Dox-inducible) expression of exogenous α-synuclein^A53T^ was combined with α-synuclein preformed fibril (α-syn pff) seeding (40, 41) (Figure 5A). Patient-derived (+/L275R) and mutated (+/L275R^Mut^) iDA neurons exhibited more pathologically phosphorylated α-synuclein (p-α-syn) fluorescence puncta than did control (+/+) and corrected (+/+^Cor^) iDA neurons (Figure 5B). More p-α-syn aggregation puncta and detergent-resistant insoluble p-α-syn aggregates were observed in patient-derived (+/L275R) and mutated (+/L275R^Mut^) iNeurons than in control (+/+) and corrected (+/+^Cor^) neurons (Figure 5, C and D, and Supplemental Figure 8E). Taken together, these results suggest that the p.L275R mutation is likely a disease-causing variant promoting PD-like synucleinopathy.

Remarkably, treatment with LHA016 at concentrations as low as 1 μM significantly reduced pathological α-synuclein aggregation in *TMEM175^+/L275R^* iNeurons (Figure 5E), but not in *TMEM175^–/–^* iNeurons (Supplemental Figure 8, F–H). Thus, pharmacological upregulation of TMEM175 H^+^ channel activity readily restores lysosomal pH homeostasis to mitigate synucleinopathy.

### Primary mouse neurons carrying the Tmem175 p.L272R mutation exhibit lysosomal hyperacidity and degradation inefficiency.

To investigate the role of the L275R mutation in vivo, we generated knock-in mice with the p.L272R mutation, equivalent to human p.L275R (Figure 6A and Supplemental Figure 9A). *I*_H_ through mouse TMEM175^L272R^ was markedly reduced in multiple primary mouse cells (Supplemental Figure 9, B and C), including hippocampal neurons, astrocytes, and bone marrow–derived macrophages (BMDMs; Figure 6, B and C, and Supplemental Figure 9, D–F). Whereas heterozygotic (*Tmem175^+/L272R^*) primary neurons and astrocytes retained roughly 30%–40% of lysosomal H^+^ currents, those from homozygotic (*Tmem175 ^L272R/L272R^*) mice lost their lysosomal H^+^ conductance completely, resembling cells from KO (*Tmem175^–/–^*) mice (Figure 6, B and C, and Supplemental Figure 9, D–F). Accordingly, *Tmem175^+/L272R^* and *Tmem175^L272R/L272R^* primary neurons exhibited lysosomal hyperacidification and degradation impairment; the defects observed in homozygotic cells (*Tmem175 ^L272R/L272R^*) were comparable to those in *Tmem175^–/–^* neurons (Figure 6, D–F, and Supplemental Figure 9, G and H).

### Mice carrying the Tmem175 p.L272R mutation show PD-like synucleinopathy and motor deficits.

As in *Tmem175^–/–^* neurons (Supplemental Figure 10, A and B), pathological α-synuclein aggregation was increased significantly in *Tmem175^+/L272R^* and *Tmem175^L272R/L272R^* neurons (Figure 6, G–J). To investigate the in-vivo roles of the L272R mutation, we performed striatal α-syn pff injection of *Tmem175^+/L272R^* and *Tmem175^L272R/L272R^* mice according to an established protocol (41). Three months after injection, cryosections and detergent-resistant insoluble proteins were collected from striatal tissues for immunostaining and immunoblotting analyses. In both assays, the levels of p-α-synuclein were significantly higher in *Tmem175^+/L272R^* and *Tmem175^L272R/L272R^* mice than in their *Tmem175^+/+^* littermates (Figure 7, A–C). Moreover, *Tmem175^+/L272R^* and *Tmem175 ^L272R/L272R^* mice exhibited certain degrees of motor deficits (Figure 7D), with the homozygous mutants resembling *Tmem175 ^–/–^* mice (Supplemental Figure 10, C–E). To determine the extent of dopaminergic neuron loss, we performed brain DAT-PET imaging (26) of genetically modified transgenic mice. All mice that received α-syn pff injections (PD modeling) showed significant reductions in ^18^F-FP-CIT intensity around the injection areas, suggesting a general loss of dopaminergic neurons (Figure 7, E and F). This reduction was most obvious in *Tmem175^L272R/L272R^* mice, with intermediate phenotypes observed for *Tmem175^+/L272R^* mice (Figure 7, E and F). Consistent with the reported transneuronal α-synuclein “spreading” effect observed in pff-seeding mouse models (42), the uptake of ^18^F-FP-CIT was reduced on the pff- and vehicle-injected sides (Figure 7, E and F). Collectively, our data from cellular and animal models suggest that the L272R knock-in mutation promotes toxic α-synuclein aggregation, causing the progressive loss of dopaminergic neurons and increasing the susceptibility to PD pathology.

## Discussion

PD risk–associated variants of genes such as *SNCA, GBA, CTSB,* and *LRRK2* are found in approximately 10%–15% of PD cases, but this rate is considerably (2–3-fold) increased in patients with early onset (at < 40 or 50 years of age) PD (5, 43–45). Hence, the likelihood of a monogenic cause may be substantially increased among individuals with early onset PD (46). *TMEM175* is a newly identified PD risk gene, but the penetrance of common variants such as p.M393T is very low (11, 15, 16, 47). Whereas TMEM175 is required for lysosomal pH homeostasis, the underlying mechanisms remain controversial (11, 22). In the current study, we identified a LOF mutation from a local PD patient that selectively abolishes proton and agonist activation of the channel without affecting K^+^ leakage activity. More importantly, by performing genetic and pharmacological studies with patient-derived fibroblasts, iPSCs, and iDA neurons, as well as knock-in mice (Figure 7G), we obtained evidence that severe LOF variants in the TMEM175 H^+^ channel may cause lysosomal dysfunction at the cellular level and synucleinopathy at the organismal level.

TMEM175 was initially characterized as a lysosomal K^+^ “leak” channel that regulates lysosomal membrane potential (14). Subsequent studies demonstrated that it can function as a proton-activated proton channel (11, 21). However, endogenous TMEM175 is barely active under the physiological lysosomal pH (4.6) (11). A recent study showed that luminal K^+^ inhibits H^+^ permeation at low millimolar concentrations, raising questions about the physiological significance of TMEM175’s proton permeation (22). In the current study, we demonstrated that the reported K^+^ inhibition is likely an experimental artifact. Indeed, extracellular K^+^ or Na^+^ had negligible effects on TMEM175-mediated H^+^ flux, even at concentrations exceeding 100 mM (11). More importantly, we identified the rare *TMEM175* variant p.L275R in a patient with early onset PD and demonstrated using electrophysiology that the L275R mutation selectively affected proton gating but not K^+^ leak channel activity. Consistent with the notion that L275R is a gating-deficiency mutation, structural studies have shown that L275 is located in the pore-forming domain of TMEM175 (20, 21). Due to its proton activation–gating effect, TMEM175 does not mediate significant H^+^ leakage at hypoacidic pHs, but can produce a large amount of H^+^ leakage at hyperacidic pHs (12, 30). Unlike the result obtained with the WT channel, the reexpression of the L275R mutant on the lysosomal membranes of *TMEM175*-KO cells did not restore lysosomal H^+^ currents. On the other hand, reduced, but not abolished, lysosomal H^+^ currents were detected in patient-derived (*TMEM175^+/L275R^*) cells due to the remaining WT allele. Hence, L275R is a gating-deficiency LOF mutation that can lead to haploinsufficiency in patients with PD carrying the monoallelic variant. The differential effects of the L275R mutation on lysosomal H^+^ and K^+^ leakage suggest that TMEM175 regulates lysosomal acidification and degradation primarily through its role in H^+^ permeation.

To complement genetic findings using mutant allele introduction and correction, we showed that small-molecule TMEM175 agonists, which can readily restore lysosomal H^+^ conductance and lysosomal functions, were sufficient to mitigate the PD-like phenotypes in patient-derived (+/L275R) cells. Although an indirect contribution of LHA016-activated K^+^ currents to lysosomal pH correction cannot be formally excluded, the background K^+^ leak (14) shared by both WT and TMEM175^L275R^, is unlikely to account for the lysosomal pH phenotypes observed in +/L275R cells. Hence, we provide both human genetic and pharmacological evidence that loss of lysosomal H^+^-stimulated H^+^ conductance, but not lysosomal K^+^ leak conductance, is a causative contributor to PD pathology related to TMEM175.

Common and rare variants in PD risk genes may contribute to PD pathogenesis (45). For *TMEM175*, odds ratios (PD versus control) for the common variant p.M393T are 1.2–2.0 in multiancestry PD cohorts (7). Whereas homozygous M393T mutations result in 30%–50% reductions in lysosomal channel activity, monoallelic and biallelic L275R mutations cause 50%–70% and greater than 90% reductions, respectively. Hence, L275R is likely a monogenic risk variant. However, we found only one PD case carrying the L275R mutation in our local cohort. The present findings therefore need to be replicated with large-scale multiancestry cohorts. To investigate the in-vivo role of p.L275R in the pathogenesis of PD, we generated *Tmem175^L272R^* knock-in mice and employed pff seeding for PD modeling. Our findings need to be validated using other PD models, such as transgenic α-synuclein A53T mice or striatal MPTP injection to induce dopaminergic neuron loss (48). Finally, building on our cellular study findings, the development of blood-brain barrier–penetrating small-molecule TMEM175 agonists may enable the in-vivo testing of a TMEM175 targeting strategy for PD intervention.

## Methods

### Sex as a biological variable.

Our study examined male and female animals, and similar findings are reported for both sexes.

### Participants and PD diagnosis.

The clinical studies of patients with PD and fibroblast isolation from patients were approved by the Ethics Committee of the Second Affiliated Hospital, Zhejiang University School of Medicine (SAHZU, no. A2023734). Following the principles of the Declaration of Helsinki, written informed consent, including consent to the publication of diagnosis-related details, images, and videos, was obtained from all participants. A 53-year-old female patient of Han nationality with PD who carried the monoallelic *TMEM175* p.L275R mutation initially presented with bradykinesia and rigidity in her left limbs at the age of 45 years. The patient was admitted to the Department of Neurology at SAHZU, and responded well to dopamine replacement therapy (Supplemental Figure 5A). According to the Movement Disorder Society clinical diagnostic criteria (49), the patient was diagnosed with clinically established PD. Skin fibroblasts were collected from the patient with written consent; fibroblasts isolated from an age-matched healthy male individual of Han ethnicity served as controls.

### Reprogramming of human fibroblasts to iPSCs and iDA neurons.

iPSCs were reprogrammed from skin fibroblasts using the CytoTune-iPS Sendai protocol (A34546; Invitrogen) and differentiated into iDA neurons using a floor plate (FP) cell-based differentiation protocol (27, 28). Briefly, iPSCs were cultured for 5 days in the FP induction medium (N1) supplemented with SB431542 (10 μM), LDN193189 (100 nM), SHH-C24 (100 ng/ml), FGF-8 (100 ng/ml), and purmorphamine (2 μM). From days 5 to 11, the medium was gradually transitioned from N1 to N2, mixing at varying percentages: 75% N1/25% N2 on days 5–6, 50%/50% on days 7–8, 25%/75% on days 9–10, and 100% N2 on day 11. The N1 medium (50 ml) consisted of 41.5 ml KnockOut DMEM, 7.5 ml KnockOut replacement serum, 0.5 ml GlutaMAX, 0.5 ml nonessential amino acids, and 2-mercaptoethanol (50 μM). The N2 medium (50 ml) consisted of 49 ml Neurobasal, 0.5 ml N-2, and 0.5 ml GlutaMAX. Starting on day 11, FP cells were passaged for expansion and cryopreservation. After 6 passages, they were dissociated with Accutase and replated onto Matrigel-coated plates. The culture medium was then switched to DA induction neurobasal medium supplemented with B27, 0.5 ml GlutaMAX, GDNF (20 ng/ml; Peprotech), BDNF (20 ng/ml; Peprotech), L-ascorbic acid (0.2 mM; Sigma Aldrich), DAPT (10 nM; Tocris), dibutyryl-cAMP (10 μM; Sigma Aldrich), and TGF-β3 (1 ng/ml; Peprotech).

To promote α-synuclein aggregation in iDA neurons, Dox-inducible exogenous α-synuclein^A53T^ was introduced into iPSCs *via* the piggyBac (PB) system (a gift from Dr. Markus Delling, University of California). PB-teton-SNCA^A53T^ plasmids and transposase plasmids were transfected into iPSCs using lipofectamine stem transfection reagent (STEM00001; Invitrogen). After 24 hours, puromycin (1 μg/ml) was added to select monoclonal cells. Western blot confirmed consistent α-synuclein^A53T^ expression across genotypes. Ten days before inoculating α-syn pff into iDA neurons, Dox (2 μg/ml) was added to induce α-synuclein^A53T^ expression.

### Rapid transdifferentiation to generate iNeurons from iPSCs.

A one-step trans-differentiation method was used to rapidly generate cortical-like iNeurons from iPSCs, as described previously (40). The PB-SNCA^A53T–sfGFP^-NGN2 construct (Addgene: #209080; RRID: Addgene_209080), a gift from Vikram Khurana (Harvard University), was used. The PB-SNCA^A53T^-sfGFP-NGN2 plasmids were transfected into iPSCs with transposase plasmids using Lipofectamine Stem Transfection Reagent (STEM00001; Invitrogen). After 24 hours, puromycin (1 μg/ml) was added to select monoclonal populations. Dox (0.5 μg/ml) was then added to induce transdifferentiation and express exogenous α-synuclein^A53T^ for 10–15 days. Western blotting confirmed consistent expression of exogenous α-synuclein^A53T^ among iNeurons across genotypes.

### Generation of iPSCs with mutagenic (+/L275R^Mut^) and corrective WT (+/+^Cor^) genotypes.

Mutagenic and corrective WT *TMEM175* iPSCs were generated using a CRISPR-Cas9 system (29) and single-stranded oligodeoxynucleotides (ssODNs) as homologous recombination templates. In brief, the ssODN 5’-GACACATTGGCCACCTGATAGCTGGGGCAGGTGACGCGGGGTCCTCACCAGATGTCCAGGATGAGACGCGTGGCCACGAT GGCGTAGACTCCGTCGCTGAAGGCTTCCACGCGCTCCTTGCTGAGTGGCTCGT-3’, in which the endogenous CTT codon for L was replaced with a CGT codon (reverse sequence ACG in ssODN) for R, was synthesized to generate mutagenic (*+/L275R^Mut^*) iPSCs, and the ssODN 5’-CACATTGGCCACCTGATAGCTGGGGCAGGTGACGCGGGGTCCTCACCAGATGTCCAGGATGAGAAGCGTGGCCACGATGGC GTAGACTCCGTCGCTGAAGGCTTCCACGCGCTCCTTGCTGAGTGGCTCG-3’ containing an endogenous CTT codon (reverse sequence AAG in ssODN) for L was synthesized to generate corrective WT (*+/+^Cor^*) iPSCs. A guide RNA (gRNA; 5’-GATGTCCAGGATGAGAAGCG-3’) was designed to trigger homologous recombination. The gRNA in the px330-GFP vector was transfected into target cells along with ssODNs using Lipofectamine Stem Transfection Reagent (STEM00001; Invitrogen) for 24 h, followed by monoclonal selection.

### Mammalian cell lines.

COS1, HeLa, and HEK293T cells, purchased from American Type Culture Collection (ATCC), and healthy individual/patient-derived human fibroblasts (HFs), were cultured in DMEM (11960044; Gibco) + 10% fetal bovine serum (FBS; 20% for HFs; A5256701; Gibco), and used at low passages with occasional authentication for mycoplasma contamination using a PCR kit (C0301S; Beyotime). Unless otherwise indicated, cells were transfected with Lipofectamine 3000 (Invitrogen). iPSCs were maintained in StemFlex basal medium (A3349301; Gibco) with StemFlex supplement 10× (A3349201; Gibco) on Matrigel (CLS354277; Corning)–coated plates, and passaged by dissociation with ReLeSR (100-0484; STEMCELL).

Generation of *TMEM175* KO cell lines. *TMEM175* KO HeLa and HEK293T cells were generated as described previously (11). *TMEM175* KO COS1 cells were generated using gRNAs targeting exons 9 (5’-CGGTGACCTTGCTGACGTAA-3’, 5’-ACGTCAGCAAGGTCACCGGC-3’) and 11 (5’-TGGGTCGTCCTGGCCCGCAG-3’, 5’-GCTGGGACTGTGGGTCGTCC-3’), expected to delete a 3094-bp genome sequence around exon 10 with a frameshift near the II-S1 helix. The gRNAs were cloned into a pSpCas9 (BB)-2A-puro vector (Addgene), transfected into target cells, and selected with puromycin (Gibco). Remaining cells were plated to establish monoclonal populations, and the frameshift was confirmed by PCR and DNA sequencing.

### Targeted deletion and knock-in mutation of Tmem175 in mice.

*Tmem175* KO (*Tmem175*^–/–^) mice were generated as described previously (11). For p.L272R mutagenic (*Tmem175 ^L272R/L272R^*) mice, a ~1-kb genomic DNA region spanning exon 10 and flanking intron was amplified as a template. The CTC codon for L at site 272 in the open reading frame was replaced with the AGG codon for R using site-directed mutagenesis to generate the donor vector. A gRNA targeting exon 10 (5’- CGTGGCCACACTCCTCATCC-3’) was designed to trigger homologous recombination. After the transcription of sgRNA in vitro, zygote microinjection was performed on the C57BL/6N mouse strain. Heterozygous intercrossing generated *Tmem175^L272R/L272R^* mice with the expected Mendelian ratio. The genotype of newborn *Tmem175 ^L272R^ ^/L272R^* mice was confirmed with PCR (primer pair 5’-TTAAGCATCTCTGCGTCACTTCC-3’ and 5’-CCAACAGTGGGATAAGACCTAAG-3’) and DNA sequencing (primer TTAAGCATCTCTGCGTCACTTCC). Pups of *Tmem175*^–/–^ mice, *Tmem175 ^L272R^ ^/L272R^* mice, and WT littermates were used for the dissection of primary hippocampal and cortical neurons, astrocytes, and MEFs on postnatal day 0 (P0). For the α-synuclein aggregation experiments, male mice aged 3–4 months were used. Unless otherwise stated, male mice were used for the animal experiments.

### Isolation and culture of primary mouse cells.

Neurons and astrocytes were isolated and cultured as described previously (11). Briefly, cortical and hippocampal tissues were dissected from P0 mice and treated with 0.25% trypsin at 37ºC for 30 min. Isolated neurons were plated on poly-D-lysine–coated coverslips, settled for 3 h in DMEM with 10% FBS, cultured in Neurobasal-A with 1% GlutaMAX and 2% B27, and then fed every 2 days by replacing half the medium. Astrocytes were cultured in poly-D-lysine–coated flasks in DMEM/F12 with 20% FBS and 1% GlutaMAX. Neurons and astrocytes cultured at 3–7 days in vitro were used for imaging. For MEFs, P0 skin was treated similarly with trypsin, then cultured in DMEM with 20% FBS and 1% GlutaMAX. BMDMs were isolated from 8–10-week-old male mice; bone marrow was processed by infusion in DMEM, filtered, centrifuged, and resuspended in DMEM with 10% FBS and M-CSF. Medium was changed on days 3 and 5, with M-CSF removed before imaging.

### Generation of TMEM175 site-directed mutants.

Site-directed mutagenesis was performed on the EGFP-TMEM175 or mCherry-TMEM175 (pEZ-M29/M55; GeneCopoeia) template with appropriate primers. PCR amplifications were performed using 2× Phanta Flash master mix (P510-01; Vazyme). All constructs were confirmed by DNA sequencing.

### Whole-cell patch-clamp electrophysiology.

Macroscopic currents were recorded in whole-cell patch-clamp mode using an Axonpatch 200B with Digidata 1440A. Unless otherwise indicated, pipette (cytosolic) and bath (extracellular) solutions contained NMDG-MSA as major ions. The pipette solution had 150 mM NMDG, 20 mM HEPES (pH 7.2, adjusted with MSA), and 2 mM MgCl_2_ to provide Cl^–^ for stable conduction. Bath solution pH (7.2–3.0, adjusted with MSA) was varied with buffers (20 mM HEPES, 10 mM MES, and 10 mM citrate) with a pKa near the target pH. Bath solutions were applied *via* perfusion for rapid exchange. An agar bridge filled with 1 M KCl served as the reference electrode. Glass electrodes, with a resistance of 3–5 MΩ, were used. Currents were recorded using a ramp protocol (–120 mV to +120 mV, 400 ms) at 2-s intervals, with the holding potential at 0 mV. Data were collected with Clampex 10.7 (Molecular Devices) at room temperature (22–25ºC) and analyzed with Clampfit 10.7 and Origin 2021. Unless otherwise indicated, current density (pA/pF) was measured at -120 mV.

Action potentials of iPSC-derived iDA neurons were recorded under a whole-cell current-clamp configuration using the procedure: 0 pA for 200 ms, followed by 10-pA steps ranging from 0 to 120 pA for 1,000 ms. The pipette solution contained (in mM): 140 gluconate, 20 HEPES, 2 MgCl_2_, 4 NaCl, 0.39 CaCl_2_, 1 EGTA, and 2 Na-ATP (pH 7.2, adjusted with KOH), and the bath solution contained (in mM): 145 NaCl, 5 KCl, 2 CaCl_2_, 1 MgCl_2_, 10 glucose, and 10 HEPES (pH 7.4, adjusted with NaOH).

### Whole-LEL patch-clamp electrophysiology.

Endolysosomal electrophysiology was performed in isolated enlarged endolysosomes (LELs) using a modified patch-clamp method (19). Briefly, cells were treated with 1 μM vacuolin-1 (67300; Millipore Sigma) (50) to selectively enlarge LELs for 1–12 h. To release them, a glass pipette was pressed against a cell and quickly pulled away to break the cell membrane. After forming a giga-seal with endolysosomes and compensating capacitance transients, a few hundred millivolt voltage steps (1–10 ms) were applied to rupture the vacuolar membrane (51). The whole-LEL setup was confirmed by the return of capacitance transients after break-in.

Unless otherwise stated, the bath (cytosolic) and pipette (luminal) solutions contained NMDG-MSA as major ions. The bath (cytosolic) solution contained (in mM): 150 NMDG and 20 HEPES (pH 7.2, adjusted with MSA). The pipette (luminal) solution contained (in mM): 150 NMDG, 10 HEPES (pH adjusted with MSA), supplemented with 10 MES (for pH 4.6) or citric acid (for pH 3.0). The lysosomal transmembrane potential (V_m_) was defined as V_Cytosol_ – V_Lumen_ (9) to match whole-cell recordings (V_m_ = V_Cytosol_ – V_Extracellular_). A ramp protocol (–120 to +120 mV, 400 ms, holding potential at 0 mV unless otherwise indicated) was used to record the currents with 2-s intervals between ramps. Data were collected and analyzed similarly to whole-cell patch clamp.

### SSM-based electrophysiology.

SSM measurements were obtained using a SURFE²R N1 device (Nanion, Germany) as described previously (38, 39). Briefly, a lipid monolayer was applied to a functionalized gold electrode to form the SSM sensors, placed in a flow-through cuvette. After sonication, 30 μl (~2 μg lysosomal proteins) of ultracentrifuge-isolated lysosomes was adsorbed for 1 h. The solution exchange involved three phases: flow of non-activating solution (in mM: 140 NaCl, 5 MgCl_2_, 30 HEPES and 30 MES, pH 7.6) for 1 s, flow of activating solution (in mM: 50 KCl, 90 NaCl, 5 MgCl_2_, 30 HEPES and 30 MES, pH 7.6) for 1 s, then non-activating solution again for 1 s.

### Lysosomal pH determination.

Lysosomal pH was measured using the Oregon Green 488 dextran conjugate (10 Kd) ratiometric dye (D7170; Molecular Probes) as described previously (11, 52). Briefly, cells were loaded with 150 μg/ml Oregon Green 488 dextran overnight, then chased in dye-free medium for 3 hours before imaging. Imaging used a spinning-disk confocal microscope (SpinSR10; Olympus) with a 60× lens and camera (ORCA-Fusion BT; Hamamatsu) at 445/488-nm excitations, with emissions filtered at 525/50 nm. pH calibration involved preparing standard buffers (in mM: 130 KCl, 1 MgCl2, 15 HEPES, 15 MES) at pH 3.0-8.0, with 10 μM valinomycin and 10 μM nigericin, and fitting fluorescence intensity ratios (F488/F445) to generate a pH standard curve, which was used to calculate lysosomal pH from fluorescence ratios. Additionally, a chemical ratiometric pH indicator (CQ-Lyso, HY-D2119; MCE) was used (31). Cells were loaded with 5 μM CQ-Lyso in medium, incubated at 37°C for 15 min before imaging. Fluorescence emission ratios at 525/50 and 617/73 nm filter sets excited at 488 nm were converted to pH values using a standard curve.

### LysoTracker staining.

For the rough determination of lysosomal acidity, cells were incubated with 50 nM LysoTracker Red (L7528; Invitrogen) in culture medium at 37°C for 30 min, then washed twice with HBSS before imaging: microscope (IX73; Olympus), mCherry filter set (49008; Chroma).

### Detection of lysosomal hydrolytic activity.

Lysosomal cathepsin B and overall hydrolytic activities were determined using Magic Red cathepsin B assay kit (937; ImmunoChemistry) and DQ-BSA red dye (D12051; Invitrogen), respectively. Cells were incubated with Magic Red (1:1,000 dilution) or DQ-BSA (10 μg/ml) at 37°C for 1 or 16 h, then washed twice with Tyrode’s solution before imaging: microscope (IX73; Olympus), mCherry filter set (49008; Chroma).

### Detection of lysosomal GCase activity.

Lysosomal GCase activity was determined using PFB-FDGlu (HY-139452; MCE), a synthetic GCase substrate that fluoresces green upon cleavage (36). Cells were incubated with PFB-FDGlu (25 μM) at 37°C for 16 h, then washed twice with Leibovitz’s L-15 medium (11415064; Gibco) before imaging: microscope (IX73; Olympus), EGFP filter set (49002; Chroma).

### Intracellular Ca^2+^ imaging.

Ca^2+^ influx from the extracellular space to the cytosol was determined using Fluo-4 AM dye (F14201; Invitrogen). Cells were loaded with Fluo-4 AM (2 μM) and pluronic-F127 (3 μM) in culture medium at 37°C for 30 min. Prior to imaging, cells were washed with Tyrode’s solution. Imaging: microscope (IX73; Olympus), EGFP filter set (49002; Chroma).

### α-Syn pff inoculation of cultured neurons.

To facilitate p-α-syn aggregation in cultured primary mouse neurons and iDA neurons, sonicated recombinant human WT α-syn pff (2.5 mg/ml in PBS, a gift from Dr. Zhen Zhong, Zhejiang University) (53) was added to neuronal culture medium at a final dose of 3 μg/ml. Following inoculation, the culture medium was refreshed every 48 h for at least 7 days.

### Intrastriatal α-syn pff inoculation.

Recombinant human WT α-syn pff (2.5 mg/ml) was sonicated (M220; Covaris) for 30 min (30 cycles of 30 s on/off) at 10°C. Then, α-syn pff (2.5 μg) was injected into mouse brains as previously described (11, 41). Mice aged 3–4 months were anesthetized with 0.3% pentobarbitone. Sonicated pff (1 μl) or vehicle control was stereotaxically injected (10-μl syringe at 0.1 μl/min, with the needle retained for >5 min) into the dorsal striatum (0.2 mm from bregma, 2 mm from the midline, 2.6 mm deep). Mice were monitored post-surgery and sacrificed with pentobarbitone overdose 30 days later. Post-perfusion, brains were fixed in 4% paraformaldehyde overnight at 4°C, then transferred to PBS with 30% sucrose until sinking, embedded in OCT, and cryosectioned (30 μm). Sections were blocked in 3% BSA with 0.3% Triton X-100 for 1 h, then incubated overnight at 4°C with primary antibodies [α-syn phospho S129 (EP1536Y), 1:1,000; ab51253; Abcam]. After PBS washes, secondary antibodies and DAPI were applied. P-α-syn puncta areas were quantified using the “Analyze Particles” function in Fiji/ImageJ.

### Western blotting.

Cells grown to 70%–80% confluence were lysed on ice with a lysis buffer (Triton X-100, 1% v/v; Sigma) supplemented with a protease inhibitor cocktail (C0001; TargetMol) for 20 min. Lysates were centrifuged at 14,000 × *g* and 4°C for 15 min, and the supernatants collected. For p-α-syn detection, remaining pellets were rinsed twice with cold PBS and lysed in SDS buffer (2% v/v) under sonication to get the supernatant as the detergent-resistant insoluble fraction. Protein concentrations were estimated using a bicinchoninic acid assay. Supernatants were mixed with 5× protein loading buffer, boiled at 95°C for 10 min, run on SDS-PAGE (4%–20%), and transferred to 0.2-μm PVDF membranes. Membranes were blocked in Tris-buffered saline with 5% non-fat milk and 0.1% Tween-20 for 1 h. Primary antibodies [anti–human α-syn (Syn 211), 1:1,000, AHB0261, Thermo Fisher Scientific; anti–mouse α-syn, 1:1,000, 610787, BD Biosciences; anti–α-syn phospho S129 (EP1536Y), 1:1,000, ab51253, Abcam; anti–β-tubulin III, 1:2,000 T8660, Sigma; anti–β-actin (13E5), 1:1,000, 4970S, CST; anti–microtubule-associated protein 2 (MAP2), 1:1,000, AB5622, Sigma; anti-LAMP1, 1:1,000, ab24170, Abcam; anti–cathepsin B, 1:1,000, 31718S, CST; anti–cathepsin D, 1:1,000, A19680, Abclonal; anti–glucosylceramidase β (OTl1 D12), 1:1,000, MA5-26589, Invitrogen; and anti-GFP, 1:1,000, 11814460001, Sigma] were diluted in a blocking solution (PBS with 0.1% Tween-20 and 2% BSA) proportionally as indicated and covered with PVDF membranes overnight at 4°C. The Amersham Imager 680 (Cytiva) was used to image PVDF membranes after incubation with a secondary antibody [goat anti-rabbit immunoglobulin G (IgG) heavy and light chains horseradish peroxidase (HRP), 511203, Zenbio or goat anti-mouse IgG HRP, 7076S, Cell Signaling Technology] at room temperature for 1 h. Band intensities were quantified using Fiji/ImageJ.

### Immunolabeling.

Cells on coverslips were washed with PBS, fixed with 4% paraformaldehyde or cold methanol for 20 min, then permeabilized with 0.3% Triton-X 100 in blocking solution (PBS with 2% BSA). Cells were incubated with a primary antibody overnight at 4°C and a secondary antibody for 1 h at room temperature. Primary antibodies [anti–human LAMP1 (H4A3), 1:200, ab25630, Abcam; anti-hemagglutinin (3F10), 1:200, 11867423001, Roche; anti–tyrosine hydroxylase, 1:500, sc-25269, Santa Cruz Biotechnology; anti-MAP2, 1:500, ab5622, Sigma; anti–α-syn phospho S129 (EP1536Y), 1:1,000, ab51253, Abcam; and anti–β-tubulin III, 1:2,000, T8660, Sigma] and secondary antibodies (1:1,000) were diluted into the blocking solutions proportionally as indicated. Coverslips were mounted on glass slides with mounting solution (BL701A; Biosharp). Images were acquired with a confocal microscope (SpinSR10; Olympus) with a 60× oil lens or a super-resolution structured illumination microscope (MI-SIM; CSR Biotech) with a 100× oil lens.

### Real-time quantitative PCR (qPCR).

Total RNA was extracted from samples using a SteadyPure Quick RNA Extraction Kit (Accurate Biotechnology). First-strand cDNA was synthesized from purified RNA using an Evo M-MLV RT Mix Kit (Accurate Biotechnology). Real-time PCR was performed with synthesized cDNA, gene-specific, intron-spanning primers (*TMEM175*: 5’-GTACGCATTCCACTTCCCG-3’, 5’-CGTGTCGTCGGTACAGAGC-3’; *ACTB*: 5’-ACAGAGCCTCGCCTTTGCC-3’, 5’-GATATCATCATCCATGGTGAGCTGG-3’) and SYBR Green master mix. Relative mRNA levels were normalized to *ACTB* (β-actin).

### Wire-hanging test.

Mouse muscle strength was assessed with a wire-hanging test three months after α-syn pff injection. Mice underwent 5-minute tests once daily for three days. Duct tape around a wire cage lid prevented climbing. Mice hung upside down, gripping with forepaws and hind paws, attached approximately 1 m above bedding. The time to fall onto bedding was recorded, with 300 s as the cut-off.

### Rotarod test.

To assess motor coordination and balance, the rotarod test was performed using a Touchscreen Rota Rod device (LE8205, 76-0770; Panlab). Three months after α-syn pff injection, mice were placed in separate lanes on the rod, which started at 4 rpm to assess walking and balance. The speed increased to 10 rpm over 300 s. Between trials, the apparatus was cleaned with diluted ethanol. The procedure was repeated three times at 30-min intervals.

### DAT-PET visualization of dopamine transporters in mouse brains.

Micro-DAT-PET/CT imaging was performed on mice 5–6 months after α-syn pff injection using the Vista eXplore system (Sedecal) as described previously (54). The radiotracer ^18^F-FP-CIT (synthesized by the Department of Neurosurgery, SAHZU) was used to specifically “visualize” dopamine transporter density and activity in mouse brains (25). An ^18^F-FP-CIT probe [dose: 1.75 MBq (50 μCi)/10 g body weight] was injected into the tail vein. A 20-min static CIT PET scan and subsequent 49-s CT scan were performed 60 min post-injection with the mouse under 1.5% isoflurane anesthesia. PET images were reconstructed using the three-dimensional ordered subset expectation maximization method with a fixed threshold. Volumes of interest in different brain regions, including the cortex, hippocampus, striatum, hypothalamus, amygdala, cerebellum, thalamus, and midbrain, were carefully delineated using the M. Mirrione-T2 RI template in PMOD v3.902 software (PMOD Technologies). Standardized uptake values (SUVs) for CIT PET uptake were calculated and expressed as the ratio between lesion-side striatal and cerebellar (reference) SUVs.

### Statistics.

Data are presented as means ± SEM. For experiments with 2 groups, a 2-tailed Student’s *t* test was used to assess the significance of differences. For experiments with more than 2 groups, 1-way ANOVA with Šidák’s multiple-comparison test (abbreviated as 1-way ANOVA) was used to reduce the type 1 error and obtain adjusted *P* values. The significance level was set to *P* < 0.05.

### Study approval.

All animals were used with the approval of Zhejiang University’s Institutional Animal Care and Use Committee (protocol # ZJU20230162) and maintained under a standard 12/12-hour light/dark cycle with ad libitum access to food and water.

### Data availability.

All data are available in the main text or supplemental materials. Plasmids and cell lines newly generated in this work are available upon request to the corresponding author. Values for all data points in graphs are reported in the Supporting Data Values file.

## Manuscript information

### Author contributions.

HX and MH designing research studies. XY, PL, ML, LW, RS, ZJ, DC, FH, WC, LZ, ZG, XC and YW conducting experiments, acquiring data, and analyzing data. ZC, WY, WL, and LY providing reagents. XY, PL, XL, MH and HX writing the manuscript with critical input from all the coauthors. All authors reviewed and approved the manuscript.

### AI disclosure.

The authors declare that no AI tools were used in the original research for or writing of this article.

### Conflict of interest.

HX is the scientific co-founder and partial owner of Lysoway Therapeutics Inc. (Boston) and Lyso-X Therapeutics Inc. (Hangzhou). The other authors declare no competing financial interests.

### Funding.

This work was supported by the following:

- National Science and Technology Major Project of China, 025ZD0215900 (to MH).
- National Science Foundation of China 32421001 (to HX and MH), 32570805 and 32300565 (to MH), U25D9014 (to HX).
- National Key R&D Program of China 2025YFA1804300 (to HX).
- Investigator Program of the New Cornerstone Science Foundation (to HX).

## Supplementary Material

Supplemental data

Unedited blot and gel images

Supporting data values

## Figures and Tables

**Figure 1:**
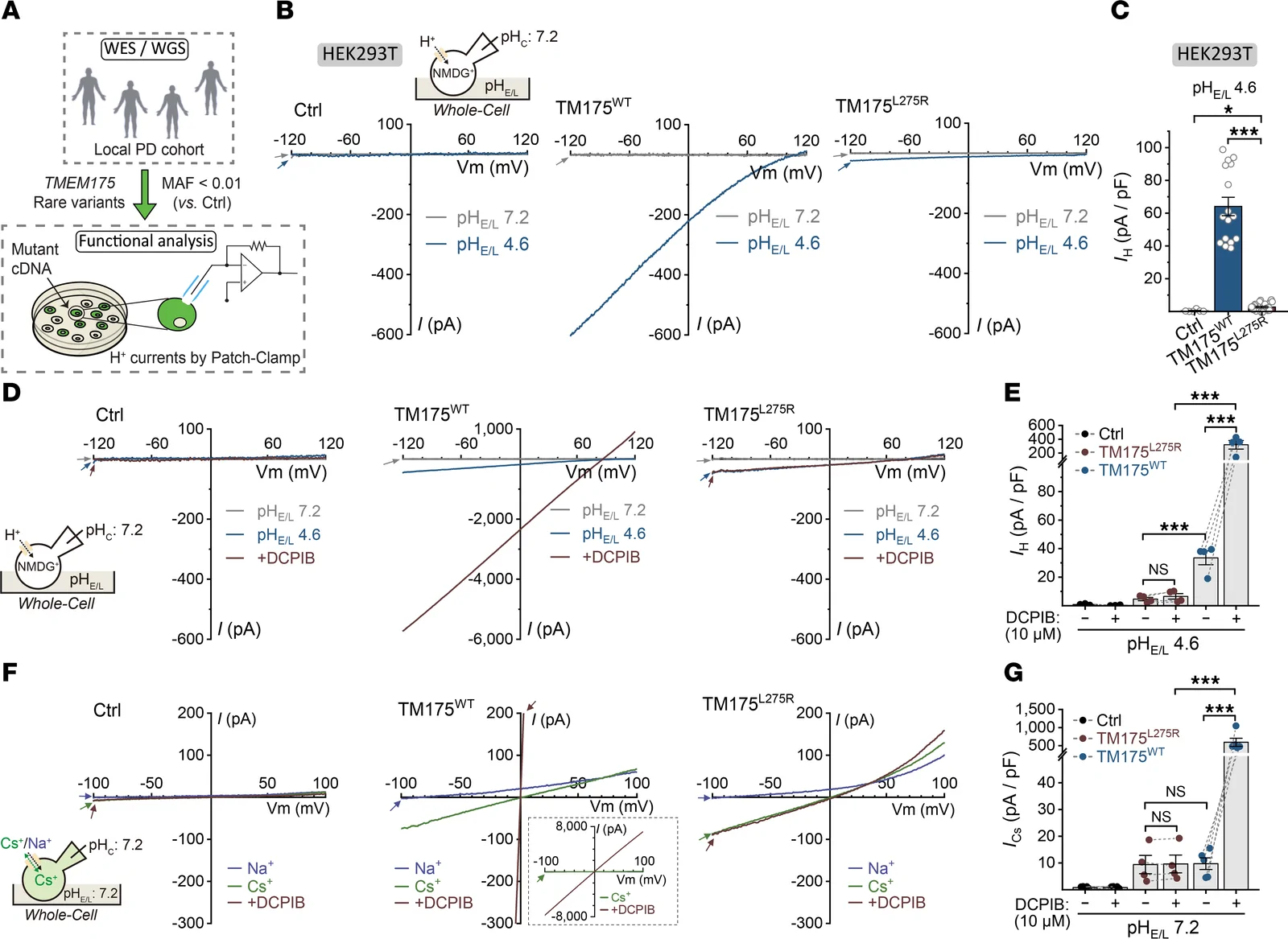
The PD patient–derived L275R mutation selectively disrupts the activation gating of the TMEM175 channel. (**A**) Diagram of whole-cell patch-clamp recordings of H^+^ currents through TMEM175 channels carrying PD patient–derived rare variants (MAF < 0.01) in a heterologous overexpression system. *TMEM175* rare variants were identified from a WES/WGS database for a local PD cohort. (**B** and **C**) Whole-cell H^+^ currents recorded in HEK293T cells overexpressing TMEM175^WT^ or TMEM175^L275R^ with cytosolic pH (pH_C_) set to 7.2, and extracellular/luminal pH (pH_E/L_) varied as indicated. Nontransfected cells served as the negative control (Ctrl). Unless otherwise indicated, all recording solutions contained NMDG^+^ and MSA^–^ as the major ions; *n* = 6 (Ctrl); 16 (WT); 27 (L275R) cells. (**D** and **E**) Whole-cell H^+^ currents recorded in HEK293T cells overexpressing TMEM175^WT^ or TMEM175^L275R^ in response to DCPIB (10 μM); *n* = 3–4 cells. Each dashed line represents the same cell before and after DCPIB application. (**F** and **G**) Whole-cell Cs^+^ currents recorded in HEK293T cells overexpressing TMEM175^WT^ or TMEM175^L275R^. The recording solutions contained 140 mM Cs or Na-MSA (pH 7.2); *n* = 4–5 cells. Unless otherwise indicated, data are means ± SEM with dots representing samples from ≥ 3 biological replicates. ***P < 0.001, *P < 0.05; by 1-way ANOVA test.

**Figure 2:**
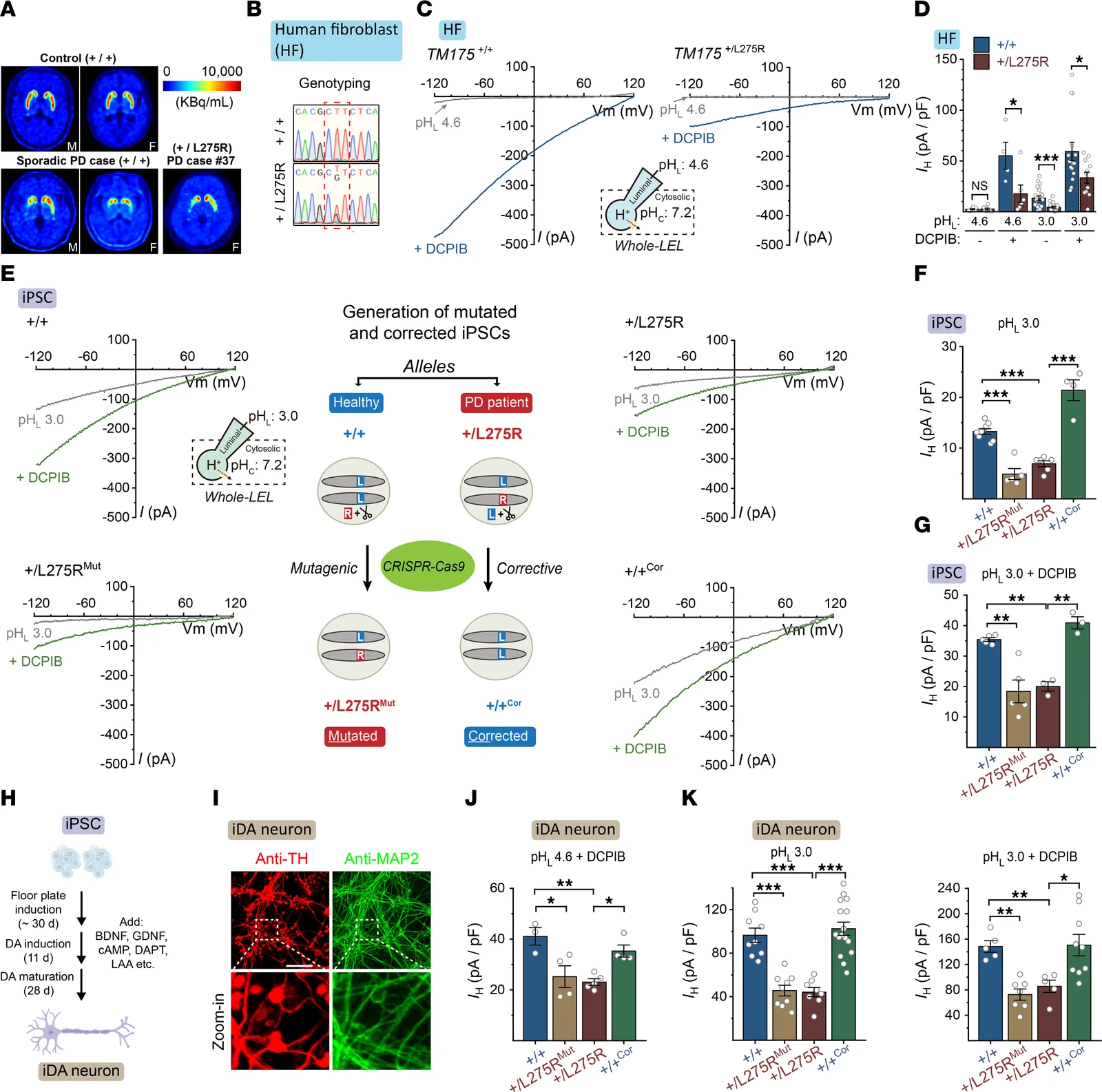
The monoallelic L275R mutation is responsible for the diminished lysosomal H^+^ currents in PD patient–derived fibroblasts, iPSCs, and iDA neurons. (**A**) Brain DAT-PET images of (i) a female (F) PD case (#37) carrying the monoallelic p.L275R variant (*TMEM175^+/L275R^*), (ii) 2 sporadic PD cases, and (iii) 2 healthy controls (*TMEM175^+/+^*). M, male. (**B**) Genotyping of human skin fibroblasts (HFs) from PD case #37 and an age-matched healthy individual. (**C**) Whole-LEL H^+^ currents from *TMEM175^+/+^* and *TMEM175^+/L275R^* fibroblasts (DCPIB: 100 μM, pH_L_: 4.6, pH_C_: 7.2). (**D**) Summary of whole-LEL H^+^ currents (at –120 mV, V_m_ = V_Cytosol_ – V_Lumen_) from experiments as in **C**; *n* = 4–22 LELs. (**E**) Whole-LEL H^+^ currents from healthy control (*+/+*), patient-derived *+/L275R*), “mutated” (*+/L275R*^Mut^), and “corrected” WT *TMEM175* (*+/+^Cor^*) iPSCs (DCPIB: 100 μM, pH_L_: 3.0, pH_C_: 7.2). Mutated (*+/L275R^Mut^*) and corrected (*+/+^Cor^*) *TMEM175* iPSCs were generated from healthy control (*+/+*) and patient-derived (*+/L275R*) *TMEM175* parental cells, respectively. (**F** and **G**) Summary of whole-LEL H^+^ currents (at –120 mV) from each group shown in **E**; *n* = 3–9 LELs. (**H**) Generation of iDA neurons from iPSCs using indicated differentiation factors. (**I**) Immunostaining of tyrosine hydroxylase (TH) and MAP2 in iDA neurons. Scale bar: 100 μm. (**J** and **K**) Summary of whole-LEL H^+^ currents (at –120 mV) from *TMEM175* (*+/+*), (*+/L275R*), (*+/L275R^Mut^*), and (*+/+^Cor^*) iDA neurons (DCPIB: 100 μM, pH_L_: 4.6/3.0, pH_C_: 7.2); *n* = 3–16 LELs. See Supplemental Figure 6 for the representative traces. Data are presented as means ± SEM, and dots represent samples from ≥ 3 biological replicates. ***P < 0.001, **P < 0.01, *P < 0.05; by 1-way ANOVA test.

**Figure 3:**
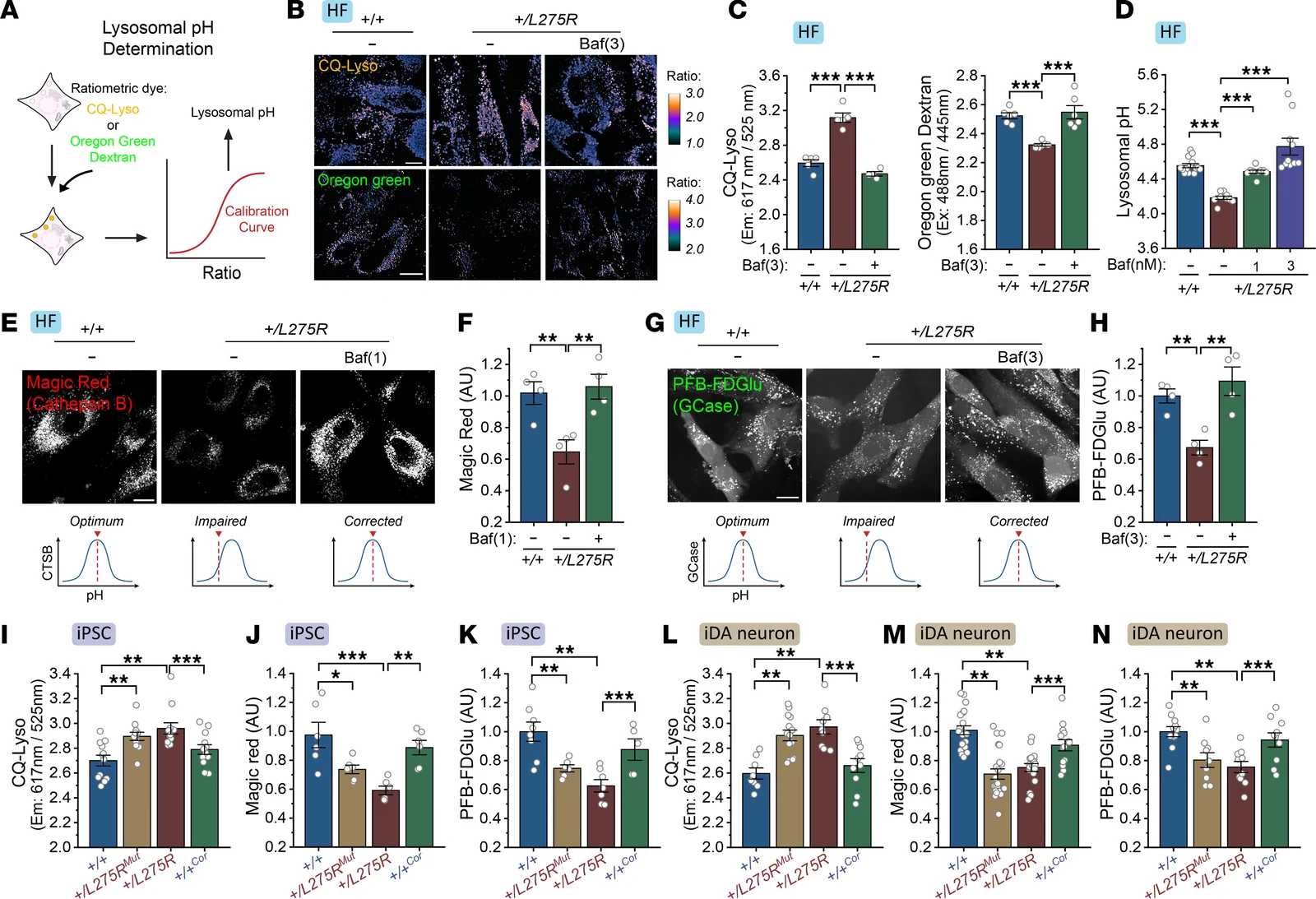
The monoallelic L275R mutation impairs lysosomal acidification and hydrolytic degradation in PD patient–derived cells. (**A**) Schematic of lysosomal pH determination using ratiometric pH indicators (CQ-Lyso and Oregon-Green-dextran). (**B**) Lysosomal luminal acidity in *TMEM175^+/+^* and patient-derived (*TMEM175^+/L275R^*) fibroblasts (HFs). A low dose (3 nM) of bafilomycin-A1 (Baf-A1; Baf[3]) was used to correct lysosomal pH abnormality in patient-derived (*TMEM175^+/L275R^*) fibroblasts. Scale bar: 20 μm. (**C** and **D**) Lysosomal pHs in HFs with the indicated genotypes and treatments. pH values were calibrated based on fluorescence ratios in **C**; *n* = 10–14 images. A low Baf-A1 dose (1 or 3 nM; Baf[1] or Baf[3]) was used for lysosomal pH normalization. (**E** and **F**) Lysosomal cathepsin B activity, assayed by Magic Red staining, in *TMEM175^+/+^* and *TMEM175^+/L275R^* fibroblasts. Scale bar: 20 μm; *n* = 4 replicates. (**G** and **H**) Lysosomal GCase activity, assayed by PFB-FDGlu green staining, in *TMEM175^+/+^* and *TMEM175^+/L275R^* fibroblasts. Scale bar: 20 μm; *n* = 4 replicates. (**I**–**N**) Lysosomal luminal acidity (CQ-Lyso staining), cathepsin B activity (MagicRed staining), and GCase activity (PFB-FDGlu staining) in iPSCs (*n* = 5–12 images) and iDA neurons (*n* = 8–17 images) of various genotypes. Data are presented as means ± SEM, with dots representing biological replicates or indicated samples from ≥ 3 biological replicates. ***P < 0.001, **P < 0.01, *P < 0.05, by 1-way ANOVA test.

**Figure 4:**
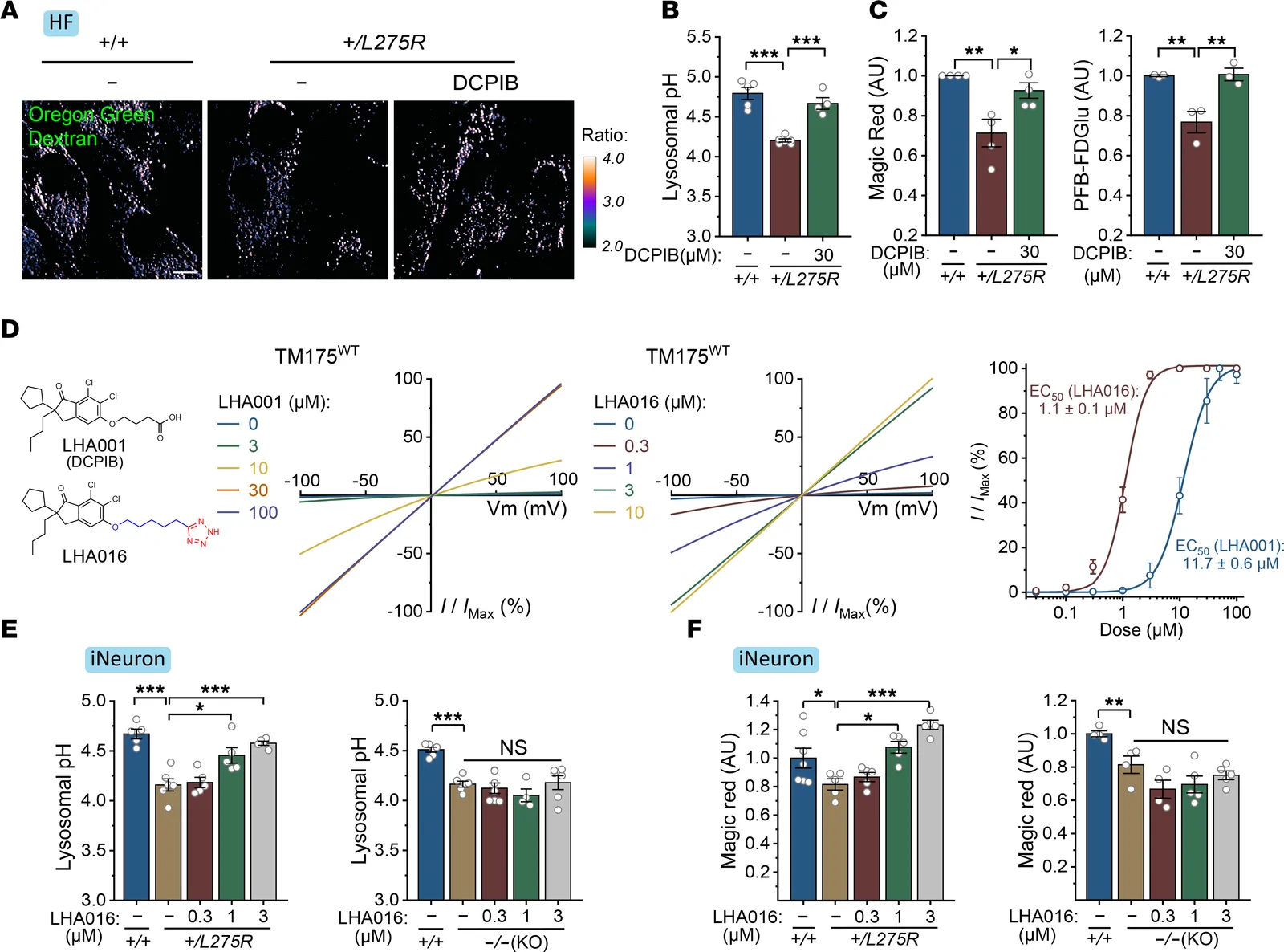
Small-molecule agonists of TMEM175 rescue lysosomal functions in patient-derived *TMEM175^+/L275R^* fibroblasts. (**A** and **B**) Lysosomal pHs in *TMEM175^+/+^* and patient-derived *TMEM175^+/L275R^* fibroblasts (HFs) with or without DCPIB (30 μΜ) treatment (Oregon Green dextran assay). Scale bar: 10 μm; *n* = 4–6 images. (**C**) Summary of lysosomal cathepsin B activity (Magic Red staining) and GCase activity (PFB-FDGlu staining) in *TMEM175^+/+^* and *TMEM175^+/L275R^* fibroblasts; *n* = 3–4 replicates. (**D**) Activation effects of DCPIB (LHA001) and LHA016 on whole-cell TMEM175 Cs^+^ currents. All solutions contained 140 mM Cs-MSA (pH 7.2). Left: chemical structures; right: dose-response curves, *n* = 3 or 4 cells. (**E** and **F**) Effects of LHA016 on lysosomal pH (*n* = 4–6 images) and cathepsin B activity (Magic Red assay, *n* = 4–7 images) in *TMEM175^+/+^*, *TMEM175^+/L275R^*, and *TMEM175^–/–^* iNeurons. Data are presented as means ± SEM, with dots representing biological replicates or indicated samples from ≥ 3 biological replicates. ***P < 0.001, **P < 0.01, *P < 0.05; by 1-way ANOVA test.

**Figure 5:**
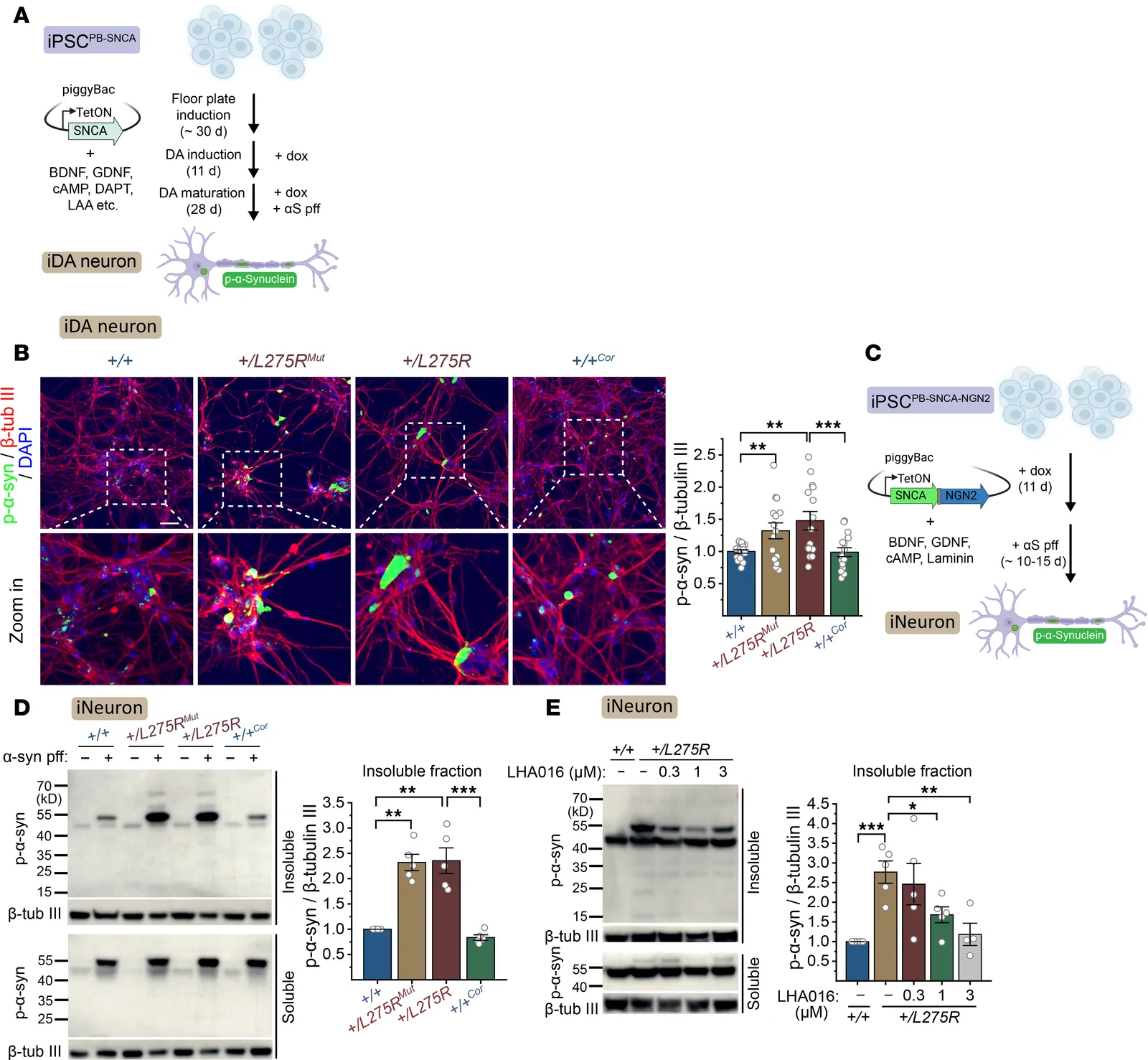
Pharmacological TMEM175 activation averts pathological α-synuclein aggregation in induced *TMEM175^+/L275R^* neurons. (**A**) Diagram of generation of iPSC-differentiated iDA neurons, in which α-synuclein A53T (PB-SNCA^A53T^) expression was induced by Dox to facilitate p-α-syn aggregation. (**B**) Immunostaining of p-α-syn aggregation in *TMEM175* (*+/+*), (*+/L275R*), (*+/L275R^Mut^*), and (*+/+^Cor^*) iDA neurons after α-syn pff (3 μg/ml) seeding for 14 days. β-tubulin III **(**β-tub III) served as a neuronal marker. Scale bar: 50 μm, *n* = 13–20 images. (**C**) Diagram of rapid transdifferentiation of iPSCs into iNeurons, in which Dox-inducible coexpression of neurogenin 2 (NGN2) and α-synuclein A53T (PB-SNCA^A53T–sfGFP^-P2A-NGN2). (**D**) Western blots of p-α-syn aggregation (detergent-resistant insoluble fractions) in iNeurons with the indicated genotypes after α-syn pff (3 μg/ml) seeding for 15 days. β-Tub III served as a neuronal loading control. Soluble fractions of p-α-syn are shown for comparison; *n* = 5 replicates. (**E**) Effects of LHA016 on p-α-syn aggregation in *TMEM175^+/+^* and *TMEM175^+/L275R^* pff-seeded (10 days) iNeurons; *n* = 4 replicates. Data are presented as means ± SEM, with dots representing biological replicates or indicated samples from ≥ 3 biological replicates. ***P < 0.001, **P < 0.01, *P < 0.05; by 1-way ANOVA test.

**Figure 6:**
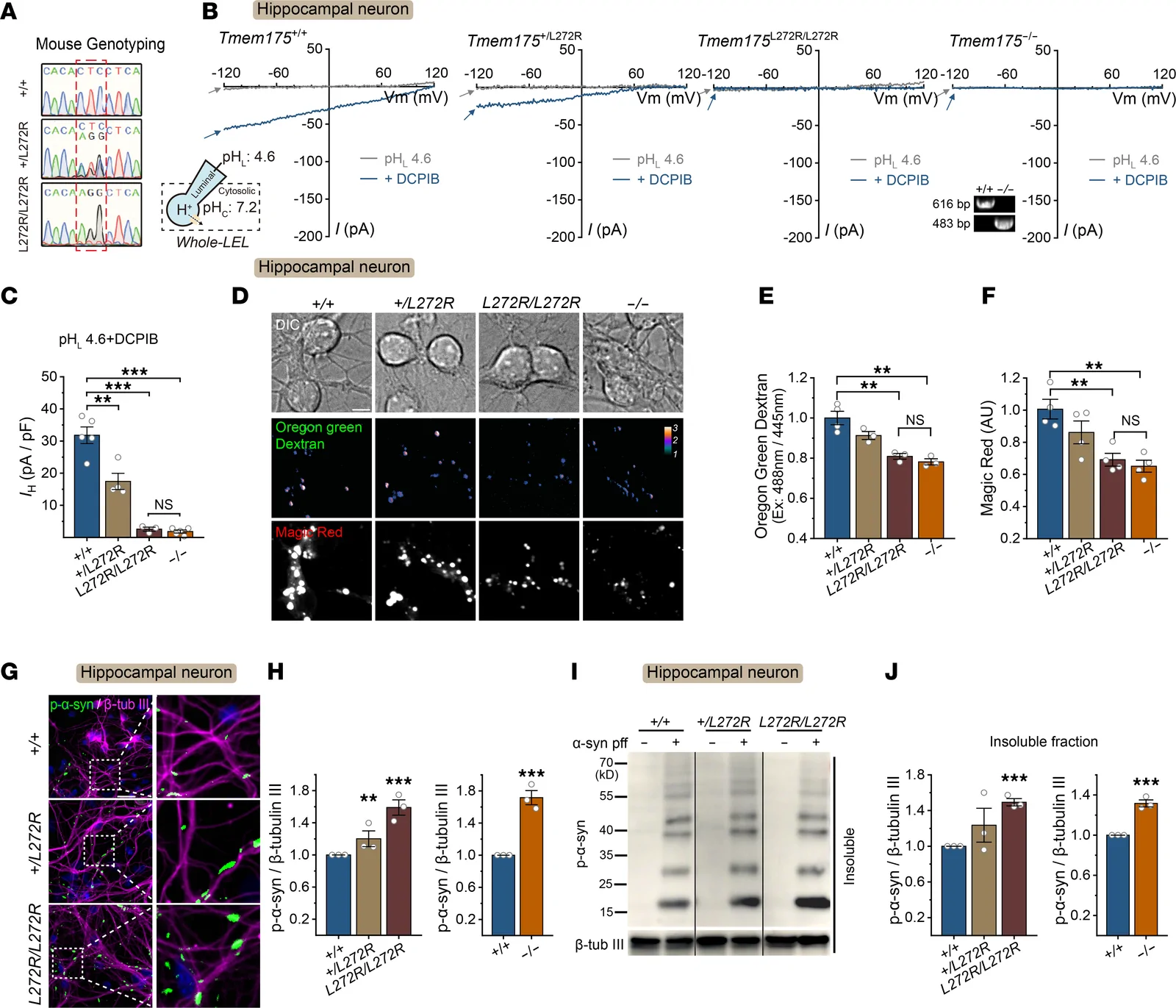
Lysosomal H^+^ currents, acidification, and hydrolytic degradation are compromised in primary neurons isolated from *Tmem175^L272R^* knock-in mice. (**A**) Genotypes of *Tmem175^+/+^*, *Tmem175^+/L272R^*, and *Tmem175^L272R/L272R^* mice. (**B** and **C**) Whole-LEL H^+^ currents in cultured mouse hippocampal neurons of the indicated genotypes in response to DCPIB (100 μM), with pH_L_ = 4.6 and cytosolic pH (pH_C_) = 7.2. Genotyping results for *Tmem175^–/–^* mice are shown on the bottom right. Summary of current densities (at –120 mV) is shown in **C**; *n* = 5 (+/+); 4 (+/L272R); 3 (L272R/L272R); 5 (-/-) LELs. (**D**–**F**) Lysosomal acidity (Oregon Green dextran assay) and cathepsin B activity (MagicRed assay) in primary hippocampal neurons isolated from mice of indicated genotypes. Scale bar: 5 μm. Summaries are shown in **E** and **F**; *n* = 3 or 4 replicates. (**G** and **H**) Immunostaining of p-α-syn aggregation in primary neurons with the indicated genotypes after pff seeding for 7 days. β-tubulin III (β-tub III) served as the neuronal marker. Scale bar: 50 μm, *n* = 3 mice. (**I** and **J**) Western blots of p-α-syn aggregation in primary hippocampal neurons isolated from mice of indicated genotypes after pff seeding for 7 days. β-tub III served as the neuronal loading control. The thin black dividing line indicates splicing of noncontiguous lanes of the same blot. For panels **H** and **J**, results from separate experiments conducted with *Tmem175*^–/–^ neurons are shown for comparison. Data are presented as means ± SEM, with dots representing biological replicates or indicated samples from ≥ 3 biological replicates. Statistical significance was determined using a 2-tailed Student’s *t* test for **H** and **J** and 1-way ANOVA test for **C**, **E**, and **F**, with ***P < 0.001, **P < 0.01.

**Figure 7:**
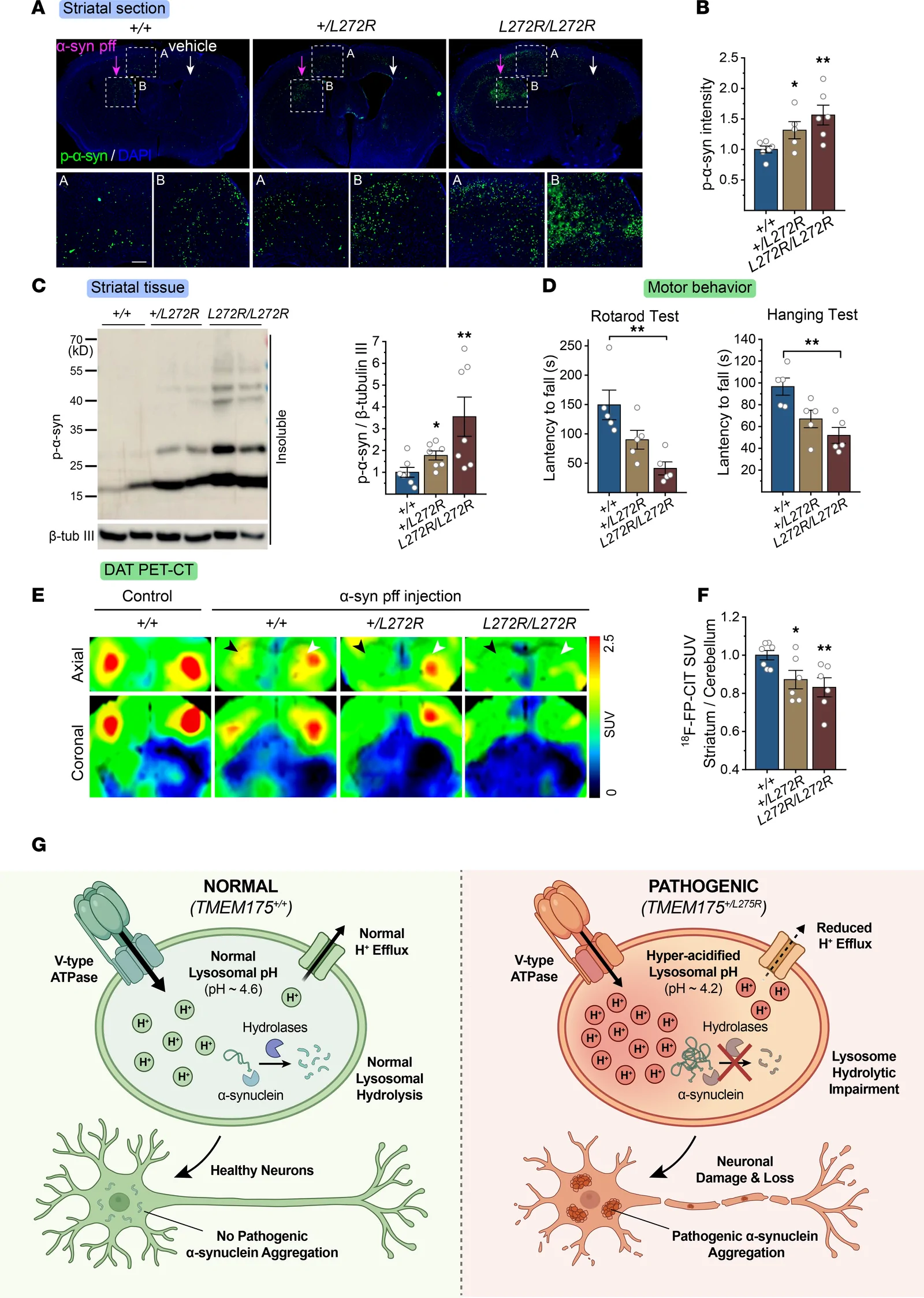
*Tmem175^L272R^* knock-in mice exhibit synuclein aggregation, dopaminergic neuron loss, and motor impairment. (**A** and **B**) Immunostaining of p-α-syn aggregation in striatal sections from mice of indicated genotypes after a single intrastriatal injection (magenta arrows) of α-syn pff (2.5 μg) for 3 months. White arrows: contralateral vehicle injection. Alphabetically labeled boxed regions are shown at higher magnification below. DAPI staining served as the nuclear control. Scale bar: 100 μm, *n* = 5–6 replicates. (**C**) Western blots of p-α-syn aggregation in brain tissues from *Tmem175^+/+^*, *Tmem175^+/L272R^*, and *Tmem175^L272R/L272R^* mice receiving single intrastriatal injections of α-syn pff (2.5 μg) for 3 months. Neuronal β-tubulin III (β-tub III) served as the loading control; *n* = 7 replicates. (**D**) Rotarod and hanging test results for *Tmem175^+/+^*, *Tmem175^+/L272R^*, and *Tmem175^L272R/L272R^* mice after a single intrastriatal injection of α-syn pff (2.5 μg) for 3 months; *n* = 5–6 mice. (**E** and **F**) Representative brain DAT-PET images from *Tmem175^+/+^*, *Tmem175^+/L272R^*, and *Tmem175^L272R/L272R^* mice after a single intrastriatal injection of α-syn pff (2.5 μg) for 5 months. Mice that received no pff injection served as negative controls. White and black arrowheads indicate the vehicle and α-syn pff injection sites, respectively; *n* = 6–7 mice. Data are presented as means ± SEM, with dots representing individual animals. **P < 0.01, *P < 0.05, by a 2-tailed Student’s *t* test. (**G**) Maintaining optimal lysosomal pH via V-ATPase (H^+^ influx) and TMEM175 (H^+^ efflux/leakage) is required for efficient hydrolytic degradation and clearance of α-synuclein aggregates in healthy dopaminergic neurons. Monoallelic expression of the *TMEM175* p.L275R mutation, which disrupts the proton gating effect on TMEM175, reduces lysosomal H^+^ leakage to cause lysosomal hyper-acidification and hydrolytic impairment. Subsequently, pathogenic α-synuclein aggregates accumulate in dopaminergic neurons, ultimately promoting the progressive loss of midbrain dopaminergic neurons and increasing PD susceptibility.

## Version 1 11/02/2026

Electronic publication
