# Supplementary material for: A rare TMEM175 mutation drives Parkinson’s disease pathology by disrupting proton gating and lysosomal acidification
Source: J Clin Invest. 2026 Nov 2;136(21):e206733. doi: 10.1172/JCI206733 (PMC13722388; doi:10.1172/JCI206733)
Supplement: Supplementary file 1 [file jci-136-206733-s148.pdf]

Supplemental Materials for

A rare TMEM175 mutation drives Parkinson's disease pathology by disrupting proton gating and lysosomal acidification

The PDF file includes:

Supplemental Figures 1–10

Supplemental Table 1

Supplementary Figures:

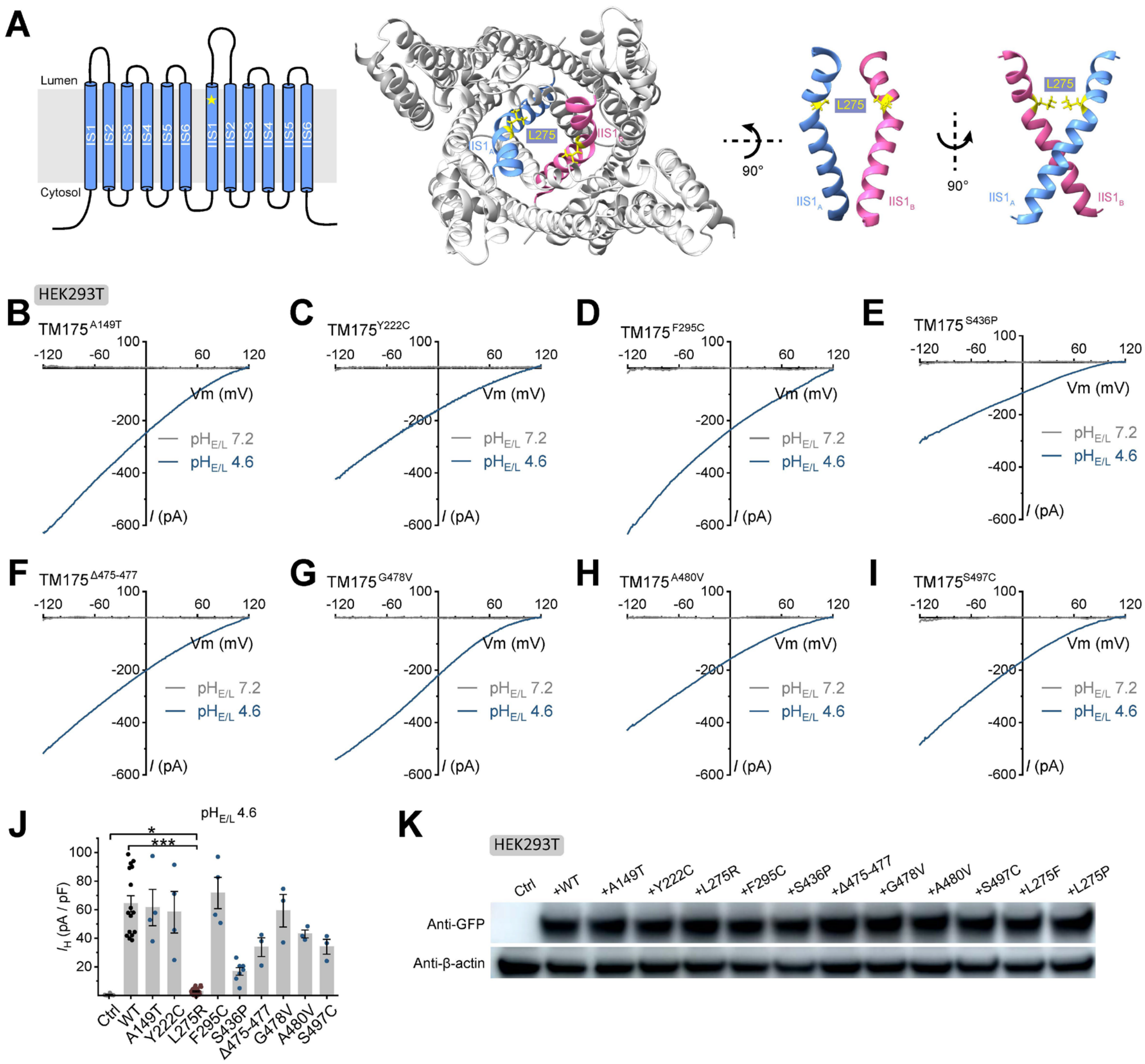

Supplemental Figure 1. LOF mutant variants were identified from a local PD cohort. (A) The membrane topology of human TMEM175, based on the reported Cryo-EM structure (PDB: 6WC9). The human TMEM175 channel is a dimer composed of two identical subunits, each containing 12 transmembrane segments with two similar repeats (IS1-IS6 and IIS1-IIS6). The structure shows a putative H^+/K^+ pathway formed by IS1 (blue) and IIS1 (pink) of both subunits. L275 locations are indicated in yellow. **(B–I)** Whole-cell H^+ currents recorded in HEK293T cells overexpressing WT and mutant TMEM175 channels, with the pH_C set to 7.2 and $pH_{E/L}$ set to 4.6; all recording solutions contained NMDG-MSA as major ions. **(J)** Whole-cell H^+ current densities (pA/pF, at –120 mV) from experiments as in (B–I); $n \geq 3$ cells. Data are presented as means ± SEM. ***$p < 0.001$, *$p < 0.05$ (two-tailed Student's *t*-test). Data from Fig. 1C were replotted here for comparison. **(K)** Western blots

of protein expression in HEK293T cells overexpressing WT and mutant EGFP-TMEM175 proteins (EGFP was fused in-frame to the N-terminus of TMEM175). β-actin served as the loading control.

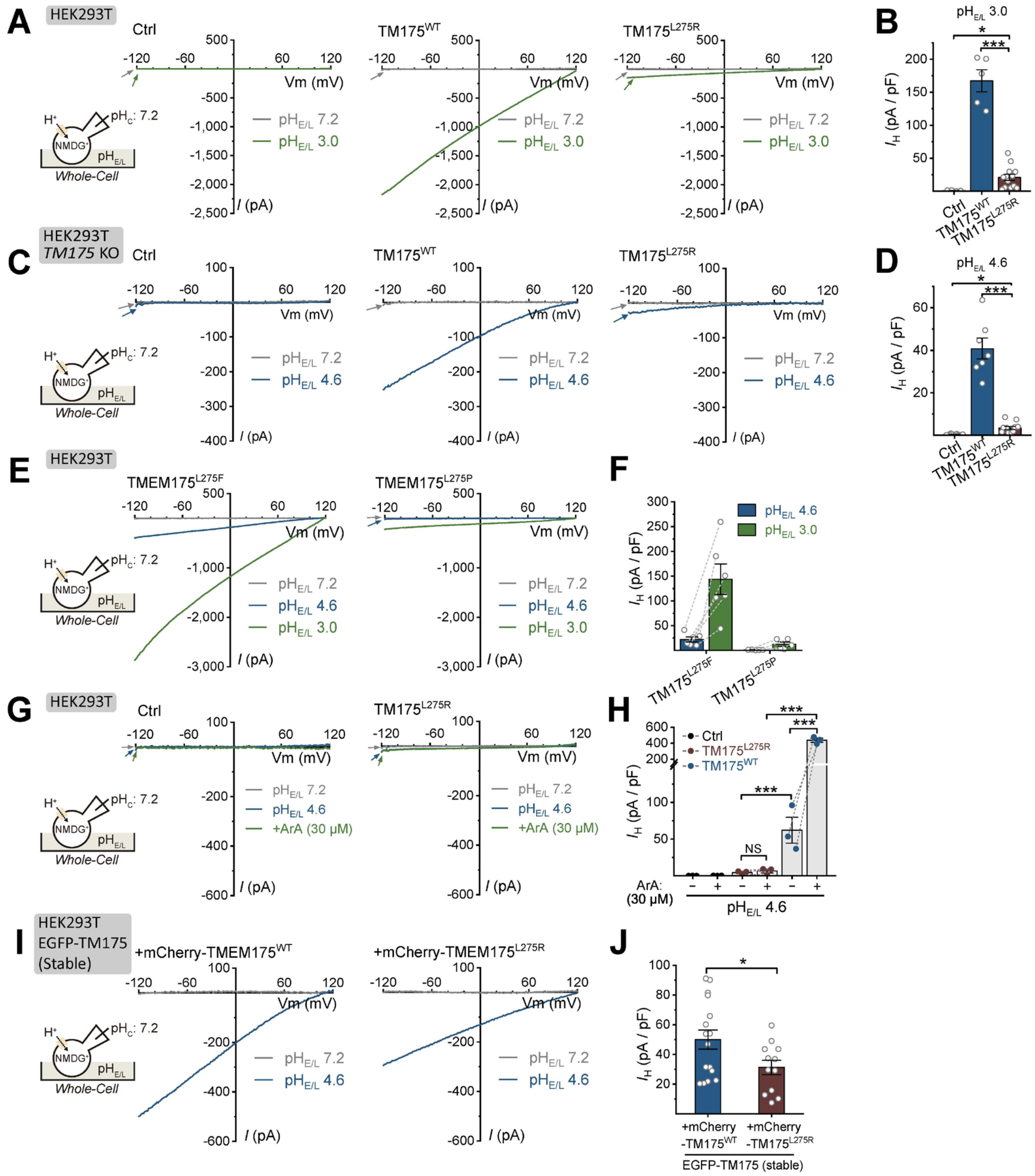

Supplemental Figure 2. L275R is a gating-deficiency mutation in TMEM175. (A, B) Whole-cell H^+ currents recorded in HEK293T cells overexpressing $TMEM175^{WT}$ or $TMEM175^{L275R}$, with the pH_C set to 7.2, and $pH_{E/L}$ varied as indicated. Non-transfected (Ctrl) cells served as negative control; *n* = 4/5/13 cells. **(C, D)** Whole-cell H^+ currents recorded following the protocols for panel (A) in *TMEM175* KO HEK293T cells overexpressing $TMEM175^{WT}$ or $TMEM175^{L275R}$; *n* = 12/7/11 cells.

(E, F) Whole-cell H^+ currents in HEK293T cells overexpressing $TMEM175^{L275F}$ or $TMEM175^{L275P}$, with pH_C set to 7.2 and $pH_{E/L}$ varied as indicated; n = 6/5 cells. (**G, H**) Whole-cell H^+ currents recorded in HEK293T cells overexpressing $TMEM175^{WT}$ or $TMEM175^{L275R}$ in response to arachidonic acid (ArA; 30 μM), with pH_C set to 7.2 and $pH_{E/L}$ varied as indicated; n = 3/3/3 cells. **(I, J)** Whole-cell H^+ currents recorded in HEK293T cells stably expressing EGFP–TMEM175, co-transfected with mCherry-$TMEM175^{WT}$ or mCherry-$TMEM175^{L275R}$, with pH_C = 7.2 and $pH_{E/L}$ varied as indicated; n = 16/12 cells (two-tailed Student's *t*-test). Data are presented as means ± SEM with dots representing samples from ≥biological replicates; Unless otherwise indicated, statistical significance was determined using a one-way ANOVA test, with ***$p < 0.001$, *$p < 0.05$; NS, $p > 0.05$.

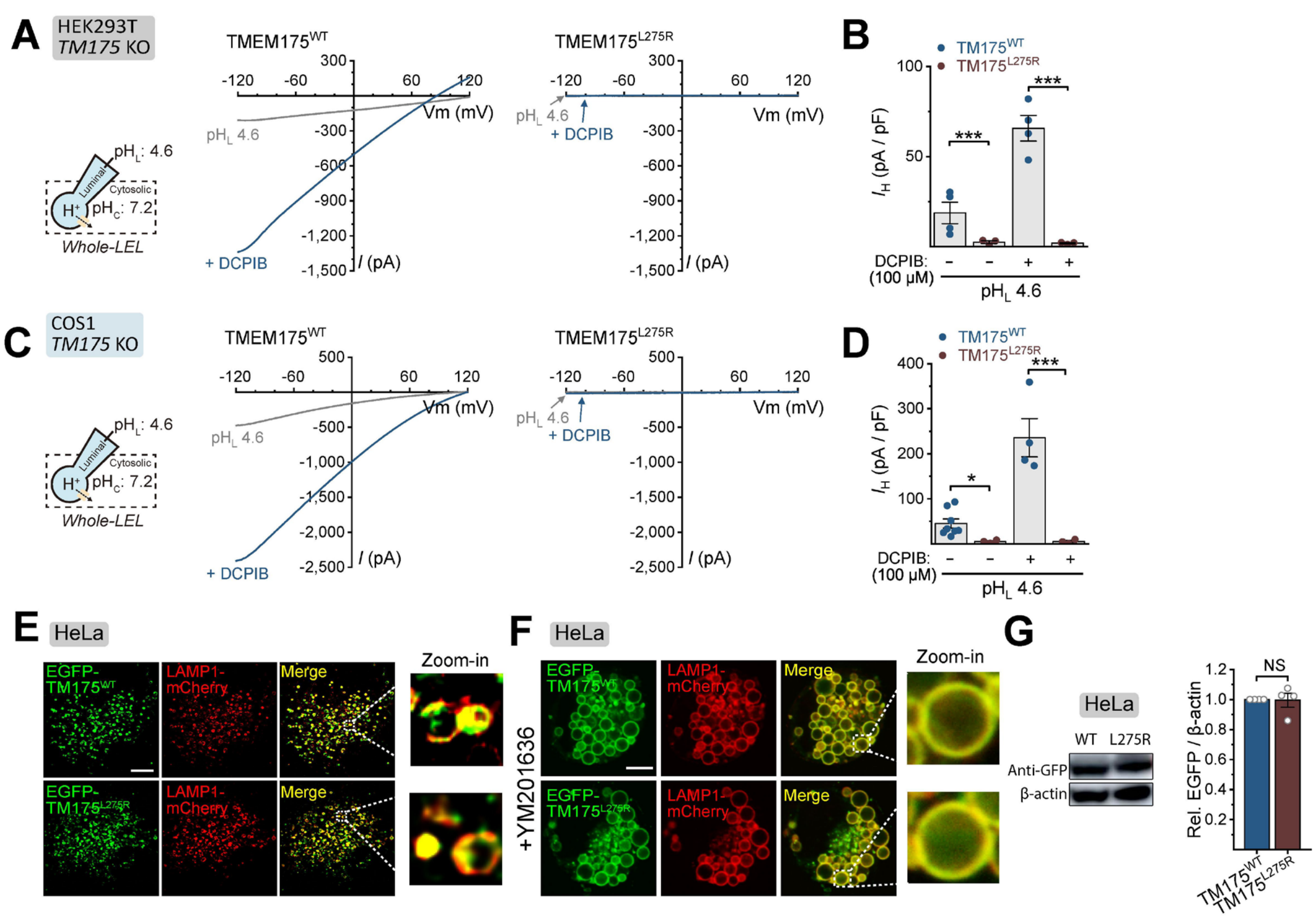

Supplemental Figure 3. L275R is a gating-deficiency mutation in TMEM175. (A, B) Whole-LEL H^+ currents recorded in *TMEM175* KO HEK293T cells overexpressing EGFP-$TMEM175^{WT}$ or EGFP-$TMEM175^{L275R}$ in response to DCPIB (10 μM), with pH_L = 4.6 and pH_C = 7.2; n = 4 cells. **(C, D)** Whole-LEL H^+ currents recorded in *TMEM175* KO COS-1 cells overexpressing EGFP-$TMEM175^{WT}$ or EGFP-$TMEM175^{L275R}$ in response to DCPIB (10 μM), with pH_L = 4.6 and pH_C =

7.2; n = 3–8 cells. **(E)** Subcellular localizations of overexpressed EGFP-TMEM175^{WT} or EGFP-TMEM175^{L275R} proteins in HeLa cells. Co-expressed LAMP1-mCherry served as the lysosomal membrane marker. Scale bar: 5 µm. Right: magnified versions of the boxed regions. **(F)** Co-residence of EGFP-TMEM175^{WT} or EGFP-TMEM175^{L275R} with LAMP1-mCherry in transfected HeLa cells. Lysosomes were enlarged with YM-201636 (1 µM for 6 h) to distinguish their perimeter membranes. Scale bar: 10 µm. **(G)** Western blots of EGFP-TMEM175^{WT} and EGFP-TMEM175^{L275R} expression in transfected *TMEM175* KO HeLa cells. β-actin served as the loading control; n = 4 replicates. Data are means ± SEM, and dots represent biological replicates or indicated samples from ≥biological replicates. ***$p < 0.001$, *$p < 0.05$; NS, $p > 0.05$, by a two-tailed Student's t-test.

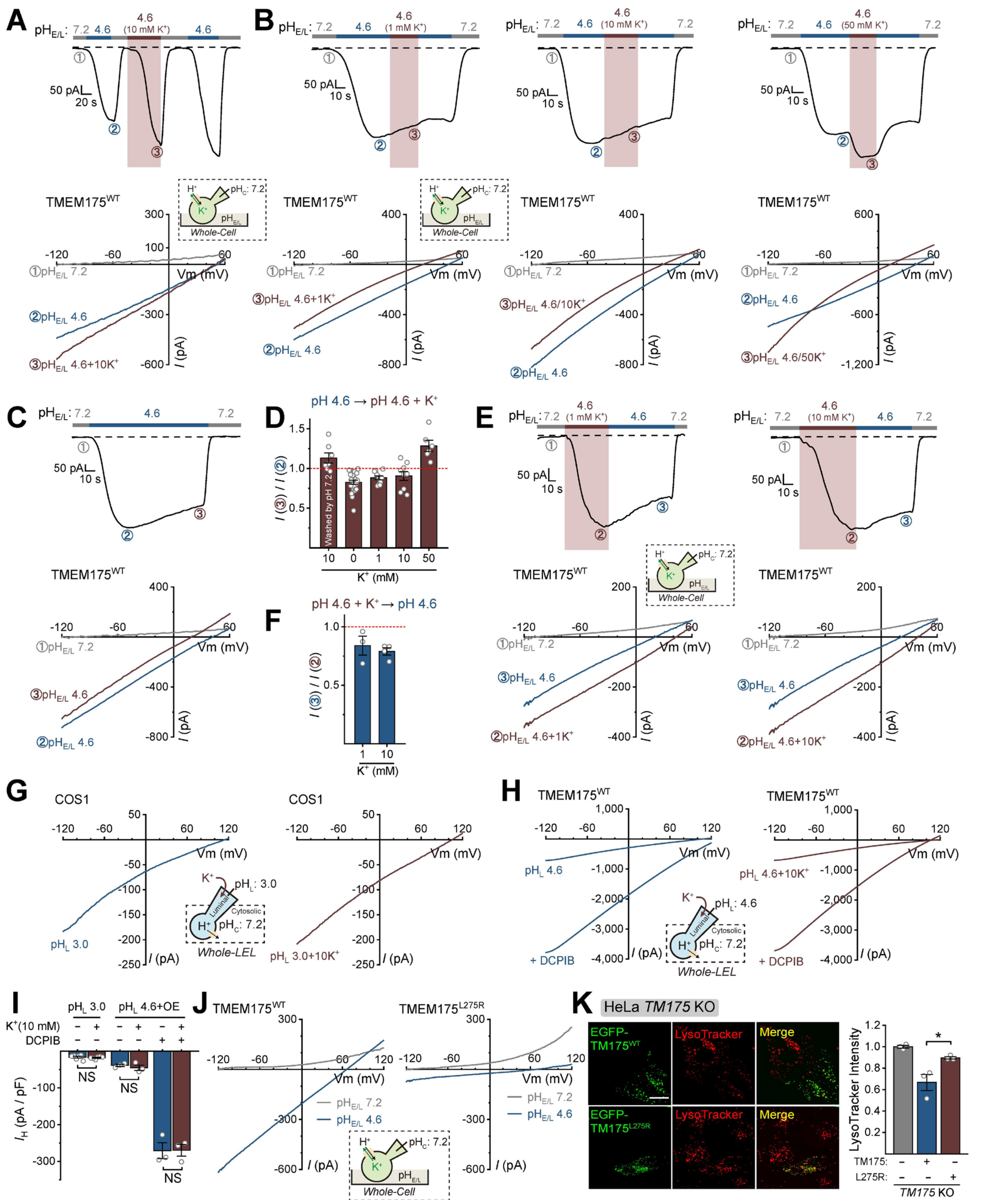

Supplemental Figure 4. K^+ had no inhibitory effect on TMEM175-mediated H^+ currents. (A–C) Whole-cell H^+ currents from TMEM175^{WT}-transfected HEK293T cells. The pipette/cytosolic solution contained K-MSA as major ions, with the pH set to 7.2. The bath/extracellular solutions

($pH_{E/L}$ 7.2/4.6) contained 0-, 1-, 10-, or 50-mM K^+ ($NMDG^+$ was substituted for equal osmolarity). The I-V relations at defined time points are shown below. The reversal potential shifted due to H^+ accumulation during continuous recordings. **(D)** Ratios of whole-cell currents (at -120 mV) before and after K^+ addition, from experiments in (A; inter-wash $pH_{E/L}$ 7.2) and (B-C); n = 7/24/11/9/6 cells. **(E)** K^+ removal had no de-inhibition effect on TMEM175-mediated H^+ currents. **(F)** Ratios of whole-cell currents before and after K^+ removal, from experiments in (E); n = 3/4 cells. **(G)** Representative whole-LEL H^+ currents in COS-1 cells with pH_L = 3.0 and pH_C = 7.2. Right: 10 mM K^+ added to the luminal solution. **(H)** Representative whole-LEL H^+ currents in COS-1 cells overexpressing EGFP-$TMEM175^{WT}$ (DCPIB: 10 µM, pH_L: 4.6, and pH_C: 7.2). (**I**) Whole-LEL H^+ current densities at –120 mV as in (G) and (H); *n* = 6/3 cells. **(J)** Whole-cell H^+ (inward) and K^+ (outward) currents recorded in HEK293T cells overexpressing $TMEM175^{WT}$ or $TMEM175^{L275R}$, with the pH_C = 7.2, and $pH_{E/L}$ varied as indicated. Pipette/cytosolic solution: K-MSA as major ions. **(K)** Lysosomal acidity in *TMEM175* KO HeLa cells re-expressing EGFP-$TMEM175^{WT}$ or EGFP-$TMEM175^{L275R}$, roughly estimated by LysoTracker red staining. Scale bar: 10 µm; *n* = 3 replicates. Data are presented as means ± SEM with dots representing biological replicates or indicated samples from ≥biological replicates. NS, $p > 0.05$, by a two-tailed Student's *t*-test.

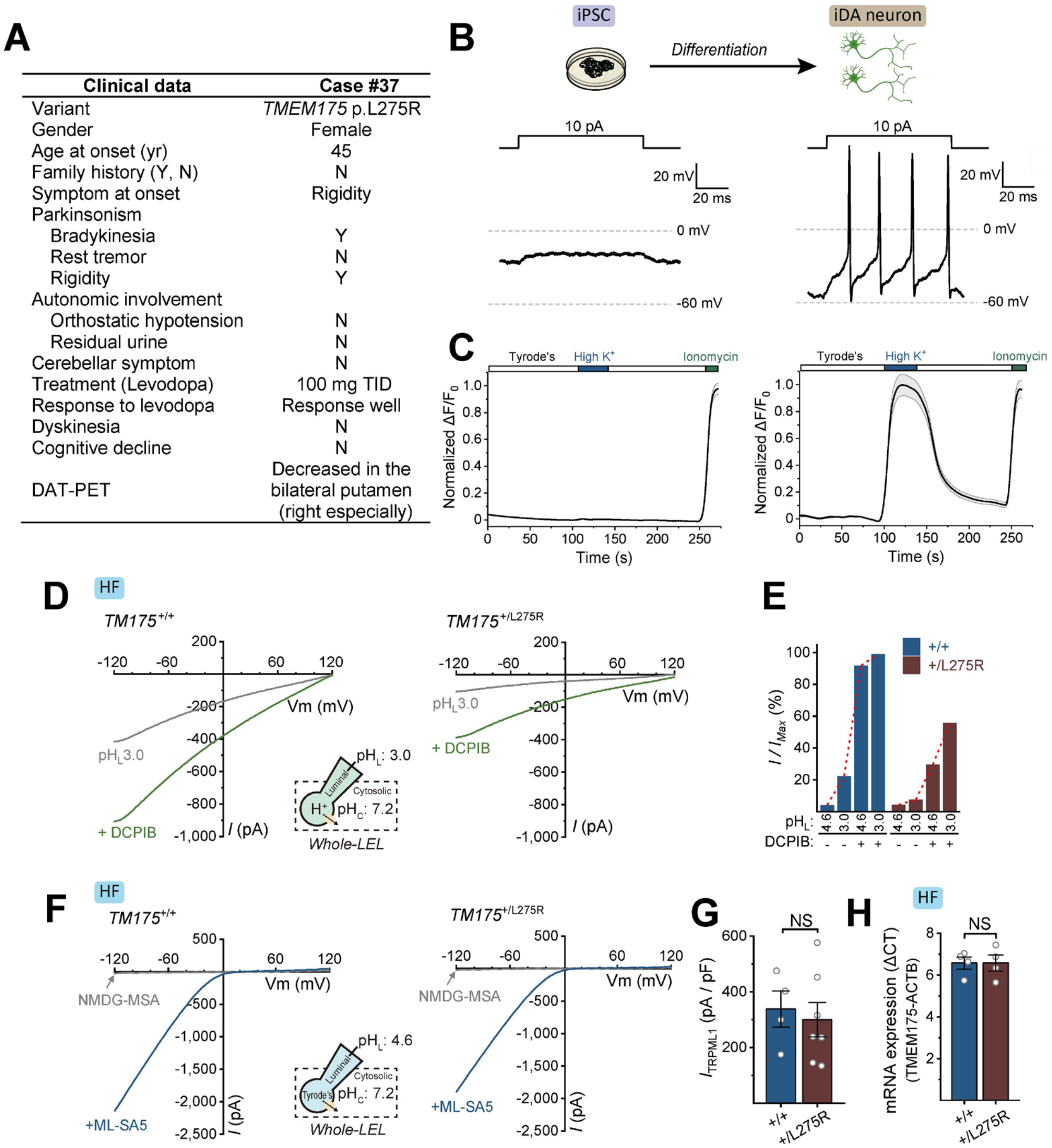

Supplemental Figure 5. The monoallelic p.L275R mutation reduced lysosomal H^+ currents in PD patient–derived fibroblasts. (A) Detailed clinical information for a PD patient harboring a monoallelic *TMEM175* p.L275R variant. **(B)** Whole-cell current clamp recordings of depolarization (inward current injection)-induced action potentials in iDA neurons. Parental iPSCs served as the control. **(C)** Ca^{2+} imaging (Fluo-4 AM dye) of iDA neurons and parental iPSCs. Neuronal Ca^{2+} responses were evoked by a high-K^+ (100 mM) solution. Ca^{2+} ionophore ionomycin served as the positive control. **(D)** Representative traces of whole-LEL H^+ currents from *TMEM175*$^{+/+}$ and *TMEM175*$^{+/L275R}$ fibroblasts in response to DCPIB (100 μM), with pH_L = 3.0 and pH_C = 7.2. **(E)** Summary of normalized whole-LEL H^+ currents (pH_L = 4.6/3.0 and pH_C = 7.2, measured at –120 mV) with or without DCPIB (100 μM) treatment. The average current density for each group was

normalized to that of the I_{max} [WT (pH_L 3.0 + DCPIB)] group. **(F)** Whole-LEL I_{TRPML1} recorded from *TMEM175*$^{+/+}$ and *TMEM175*$^{+/L275R}$ fibroblasts in response to ML-SA5 (1 μM). Luminal (modified Tyrode's, pH_L: 4.6) solutions contained (in mM) 145 NaCl, 2 $CaCl_2$, 1 $MgCl_2$, 5 KCl, 10 glucose, 10 HEPES, and 10 MES, and cytosolic solutions (pH_C: 7.2) contained NMDG-MSA as major ions. **(G)** Summary of whole-LEL I_{TRPML1} (at –120 mV) from experiments as shown in (F); n = 4/7 LELs. **(H)** *TMEM175* mRNA expression levels in *TMEM175*$^{+/+}$ and *TMEM175*$^{+/L275R}$ fibroblasts, n = 4 repeats. Dta are means ± SEM, and dots represent biological replicates or indicated samples from ≥ biological replicates. NS, $p > 0.05$, by a two-tailed Student's t-test.

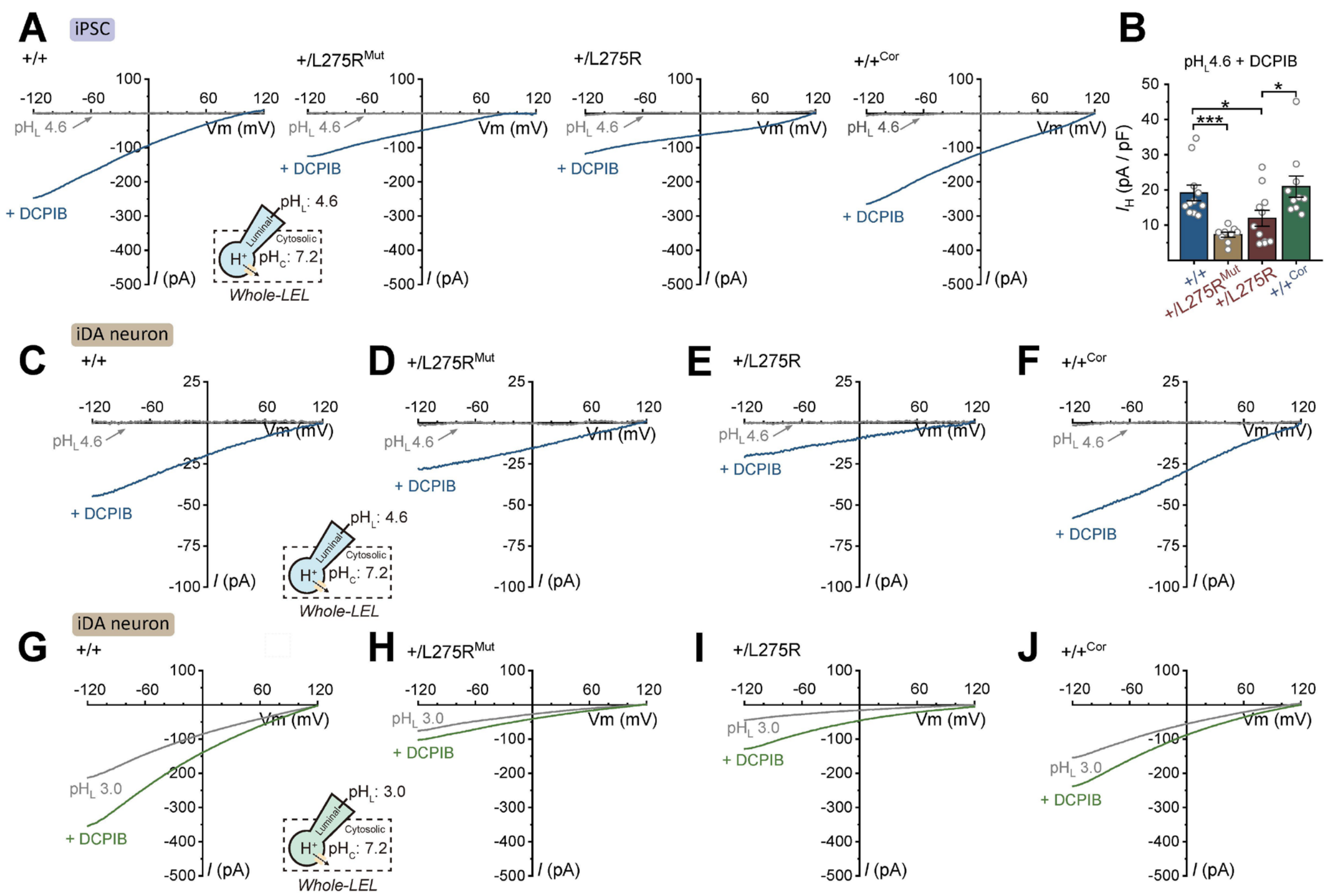

Supplemental Figure 6. The monoallelic p.L275R mutation reduced lysosomal H^+ currents in PD patient–derived iPSCs and iDA neurons. (A) Whole-LEL H^+ currents recorded from iPSCs of indicated genotypes in response to DCPIB (100 μM), with pH_L = 4.6 and pH_C = 7.2. **(B)** Whole-LEL H^+ current densities at –120 mV from experiments as in (A); n = 9-11 LELs (Data are means ± SEM, ***$p < 0.001$, *$p < 0.05$ by one-way ANOVA test). **(C–F)** Whole-LEL H^+ currents recorded from iDA neurons of indicated genotypes in response to DCPIB (100 μM) with pH_L = 4.6. See Fig. 2J for the current density summary. **(G–J)** Whole-LEL H^+ currents recorded from iDA neurons of indicated genotypes in response to DCPIB with pH_L = 3.0. See Fig. 2K for the current density summary.

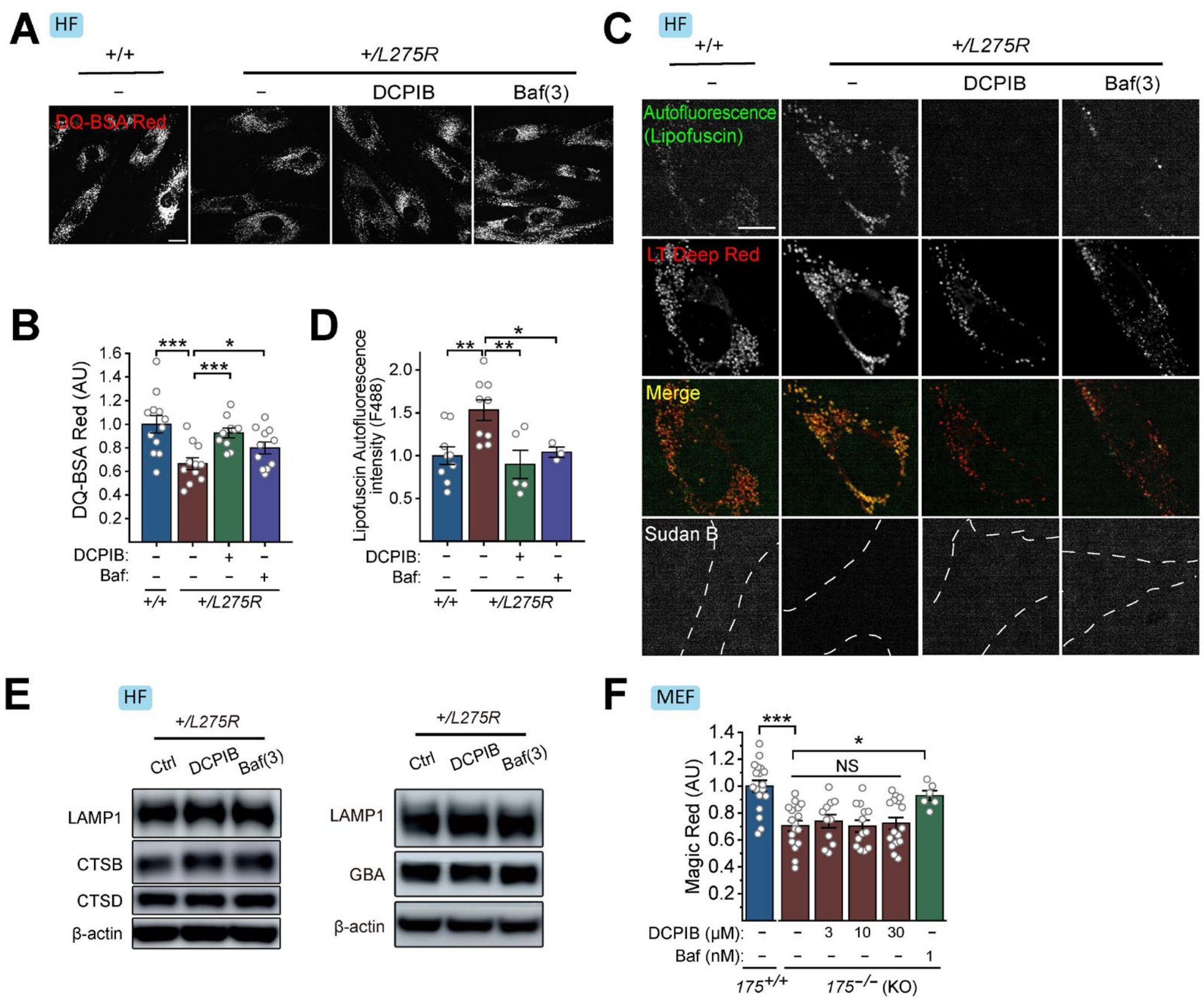

Supplemental Figure 7. The *TMEM175* p.L275R mutation impaired lysosomal hydrolytic activity. (A, B) Effects of 3 nM bafilomycin-A1 [Baf(3)] or DCPIB (30 µM) on overall lysosomal hydrolytic activity in *TMEM175*$^{+/+}$ and *TMEM175*$^{+/L275R}$ fibroblasts (DQ-BSA assay). Scale bar: 20 µm; *n* =11 or 12 images. **(C)** Effects of Baf(3) or DCPIB (10 µM) on lipofuscin levels in *TMEM175*$^{+/+}$ and *TMEM175*$^{+/L275R}$ fibroblasts. LysoTracker (LT) deep red served as a lysosomal marker. Sudan black (B; autofluorescence quencher) served as the control. Scale bar: 5 µm. **(D)** Autofluorescence at 488 nm (F488) from experiments as in (C); *n* = 3–9 replicates. **(E)** Western blots of LAMP1, CTSB, CTSD, and GBA protein expression in *TMEM175*$^{+/+}$ and *TMEM175*$^{+/L275R}$ fibroblasts treated with Baf(3) or DCPIB (30 µM). β-actin served as the loading control. **(F)** Effects of DCPIB on lysosomal cathepsin B activity (Magic Red assay) in *Tmem175* KO MEFs; *n* = 5–17 images. Data are presented as means ± SEM, and dots represent biological replicates or indicated samples from ≥biological replicates. ***$p < 0.001$, **$p < 0.01$, *$p < 0.05$; NS, $p > 0.05$, by a one-way ANOVA test.

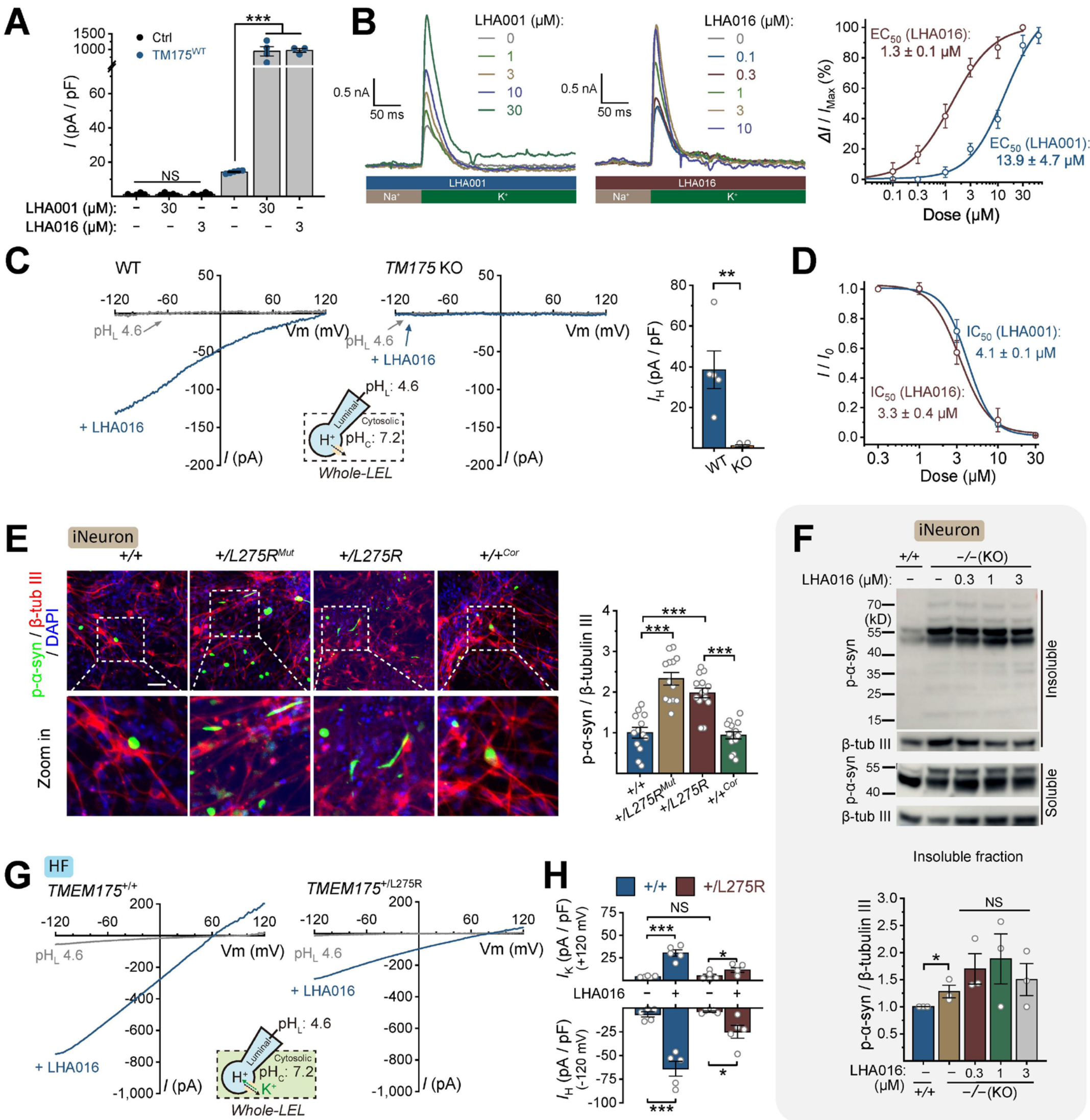

Supplemental Figure 8. LHA016, a potent small-molecule TMEM175 agonist, does not affect α-synuclein aggregation in *TMEM175* null neurons. (A) Summary of whole-cell Cs^+ currents in $TMEM175^{WT}$-transfected and non-transfected (Ctrl) HEK293T cells (LHA001: 30 μM, LHA016: 3 μM, $pH_{E/L}$ and pH_C: 7.2); n = 3–5 cells. **(B)** Representative traces of SSM-recorded K^+ currents in lysosomes overexpressing TMEM175 at different LHA001/LHA016 concentrations. Background/noise currents were recorded using the solution containing TMEM175-impermeable Na^+ as the major ions. Right: dose-response curves; n = 3-4 sensors/concentration. **(C)** Representative whole-LEL H^+ current recordings in WT and *TMEM175* KO COS1 cells (LHA016: 3 μM, pH_L: 4.6, and pH_C: 7.2); n = 5 LELs (two-tailed Student's *t*-test). **(D)** Concentration-dependent inhibition of VRAC currents by LHA016 and LHA001, n = 3 cells; **(E)** Immunostaining of p-α-syn aggregation

in iNeurons of indicated genotypes after α-syn pff (3 μg/ml) seeding for 10 days. β-tubulin III (β-tub III) served as the neuronal marker. Scale bar: 50 μm, n = 13–15 images. **(F)** Western blots of p-α-syn aggregation in *TMEM175*$^{+/+}$ and *TMEM175*$^{-/-}$ iNeurons in response to LHA016 treatment after α-syn pff seeding for 10 days. β-tub III served as the neuronal loading control; n = 3 replicates. **(G)** Representative whole-LEL currents in *TMEM175*$^{+/+}$ and *TMEM175*$^{-/-}$ fibroblasts (LHA016: 10 μM, pH_L: 4.6). Cytosolic solution contained K-gluconate as major ions (pH_C: 7.2). **(H)** Whole-LEL H^+ (–120 mV) and K^+ (+120 mV) current densities from experiments as shown in (G); n = 5 LELs. Data are presented as means ± SEM, and dots represent biological replicates or indicated samples from ≥biological replicates. ***$p < 0.001$, **$p < 0.01$, *$p < 0.05$; NS, $p > 0.05$, by one-way ANOVA test.

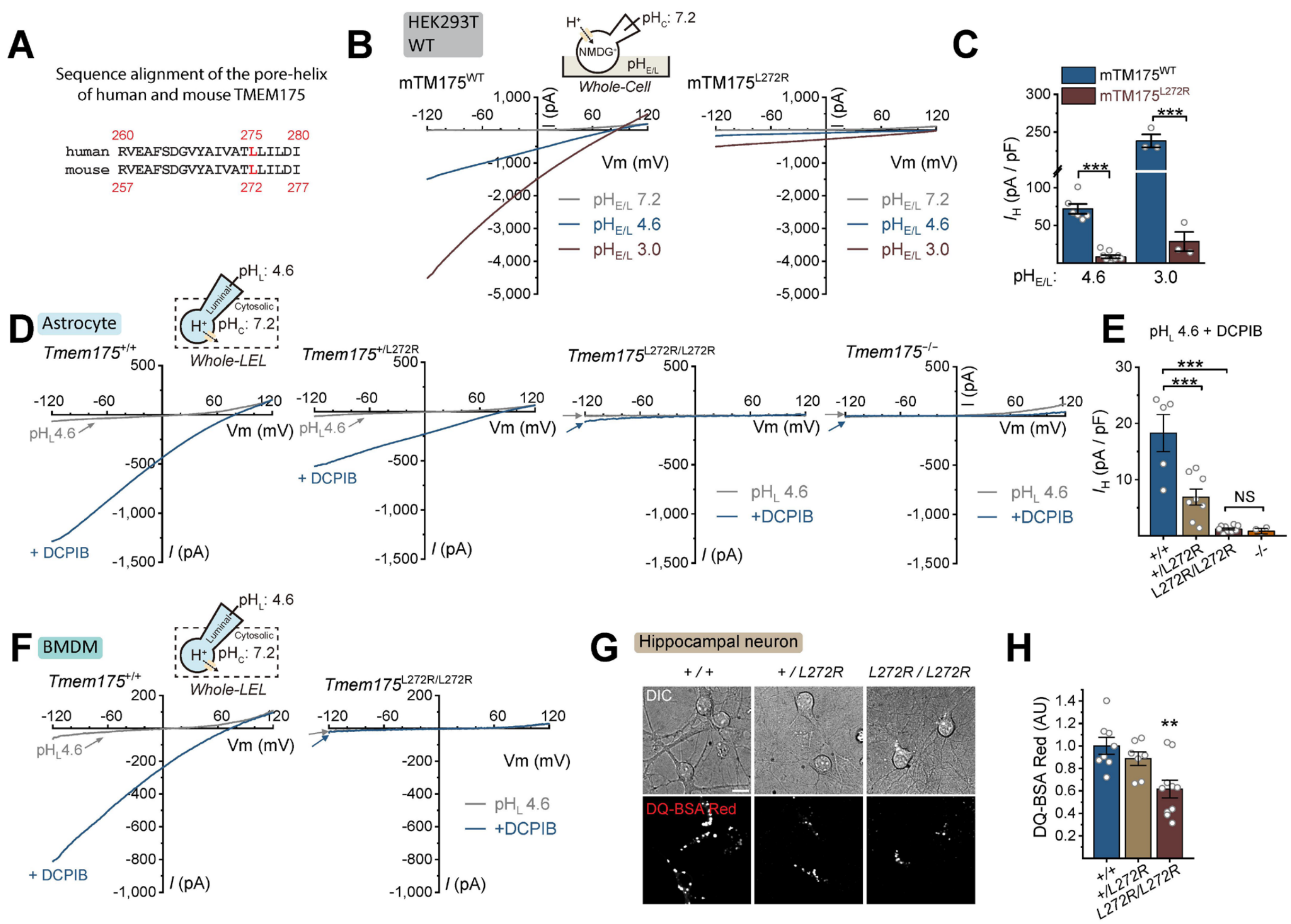

Supplemental Figure 9. The L272R mutation in mouse *Tmem175* abolishes lysosomal H^+ conductance. (A) Protein sequence alignment around the L275/L272 site in human and mouse TMEM175. **(B, C)** Whole-cell H^+ current in HEK293T cells overexpressing mouse WT TMEM175 ($mTMEM175^{WT}$) or $mTMEM175^{L272R}$ mutants, with pH_C set to 7.2 and $pH_{E/L}$ varied as indicated; n = 6/9/3/3 cells. **(D, E)** Whole-LEL H^+ currents from primary astrocytes isolated from *Tmem175*$^{+/+}$,

Tmem175$^{+/L272R}$, *Tmem175*$^{L272R/L272R}$, *and Tmem175*$^{-/-}$ mice in the presence of DCPIB (100 μM), with pH_L/pH_C = 4.6/7.2; *n* = 5/8/10/3 LELs. **(F)** Whole-LEL H^+ currents from primary BMDMs isolated from *Tmem175*$^{+/+}$ and *Tmem175*$^{L272R/L272R}$ mice in the presence of DCPIB (100 μM), with pH_L/pH_C = 4.6/7.2. **(G, H)** Overall lysosomal hydrolytic activity in primary neurons isolated from *Tmem175*$^{+/+}$, *Tmem175*$^{+/L272R}$, and *Tmem175*$^{L272R/L272R}$ mice (DQ-BSA assay). Scale bar: 10 μm; *n* = 7–10 images. Data are means ± SEM, and dots represent indicated samples from ≥biological replicates. ***$p <$ 0.001, **$p < 0.01$; NS, $p > 0.05$, by one-way ANOVA test.

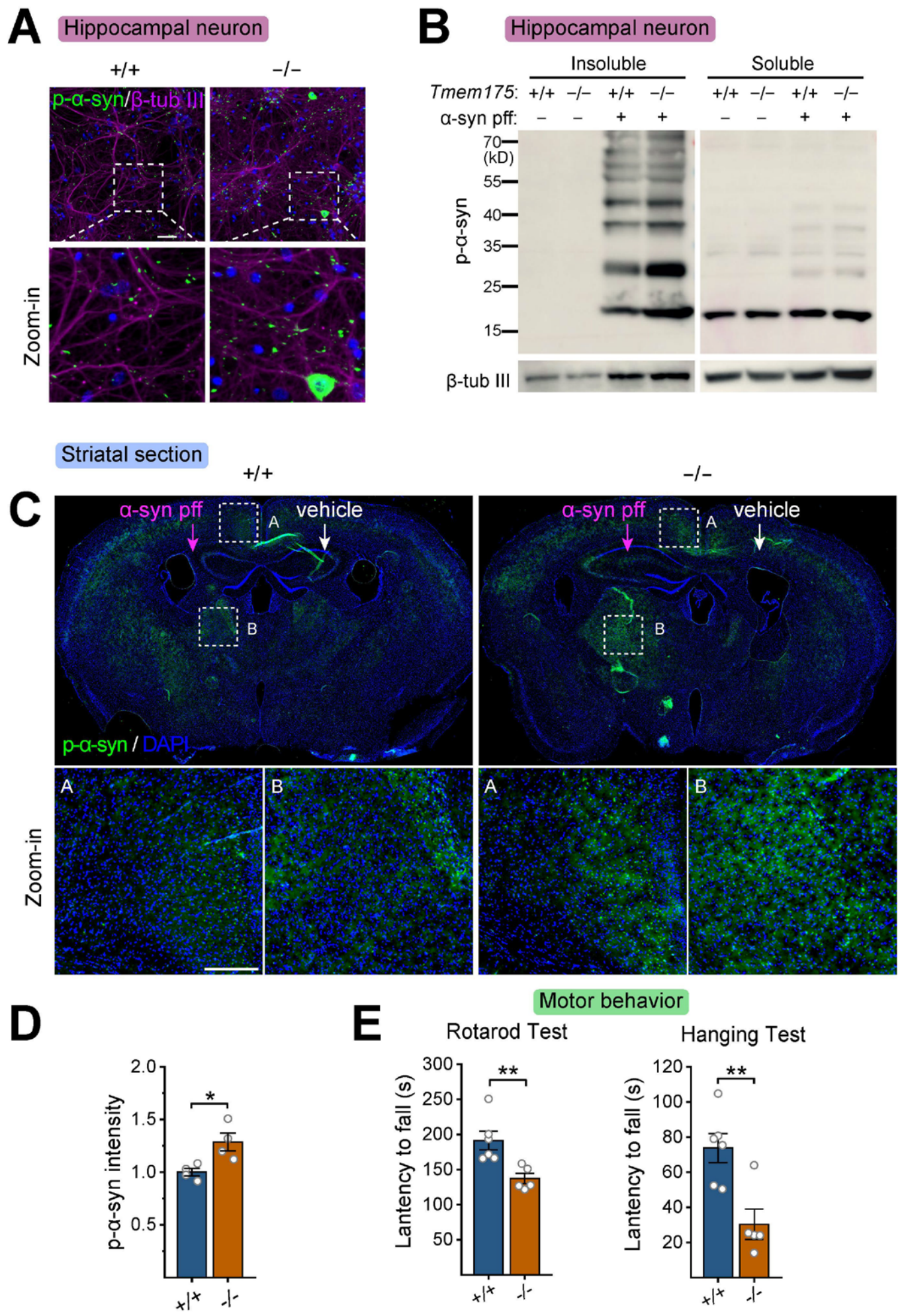

Supplemental Figure 10. Synucleinopathy was observed in *Tmem175* null mice. (A, B) Immunofluorescence staining and western blots of p-α-syn aggregation in *Tmem175*$^{+/+}$ and *Tmem175*$^{-/-}$ primary neurons after α-syn pff (3 μg/ml) seeding for 7 days. Scale bar: 50 μm. β-tubulin III (β-tub III) served as the neuronal marker and loading control. See Fig. 6H and 6J for quantification. **(C, D)** Immunostaining of p-α-syn aggregation in striatal sections from *Tmem175*$^{+/+}$ and

Tmem175$^{-/-}$ mice after a single intra-striatal injection (magenta arrows) of α-syn pff (2.5 μg) for 1 month. White arrows: contralateral vehicle injection. Alphabetically labeled boxed regions are shown at higher magnification below. DAPI staining served as the nuclear control. Scale bar: 100 μm, $n = 4$ replicates. **(E)** Rotarod and hanging test results for *Tmem175*$^{+/+}$ and *Tmem175*$^{-/-}$ mice after a single intra-striatal injection of α-syn pff (2.5 μg) for 1 month; $n = 6$ mice. Data are means ± SEM; statistical significance was determined using a two-tailed Student's *t*-test, with $^{**}p < 0.01$, $^{*}p < 0.05$.

Supplemental Table 1. TMEM175 rare variants from a local PD cohort

chr	Pos	Allele (ref>alt)	AA change	Annotation	Zygosity	GnomAD frequency
chr4	952433	c.445G>A	p.A149T	missense	Het	2.32E-03
chr4	955442	c.665A>G	p.Y222C	missense	Het	1.31E-04
chr4	955872	c.824T>G	p.L275R	missense	Het	variant not found
chr4	957865	c.884T>G	p.F295C	missense	Het	2.63E-05
chr4	958287	c.1306T>C	p.S436P	missense	Het	6.85E-06
chr4	958397	c.1424_1432delTCCTGCGGG	p.V475_R477del	cds-del	Het	5.68E-05
chr4	958414	c.1433G>T	p.G478V	missense	Het	1.30E-04
chr4	958420	c.1439C>T	p.A480V	missense	Het	1.24E-04
chr4	958471	c.1490C>G	p.S497C	missense	Het	2.57E-06

Assembly: hg38
