# Supplementary material for: A rare TMEM175 mutation drives Parkinson’s disease pathology by disrupting proton gating and lysosomal acidification
Source: J Clin Invest. 2026 Nov 2;136(21):e206733. doi: 10.1172/JCI206733 (PMC13722388; doi:10.1172/JCI206733)

Fig.5D (cropped)

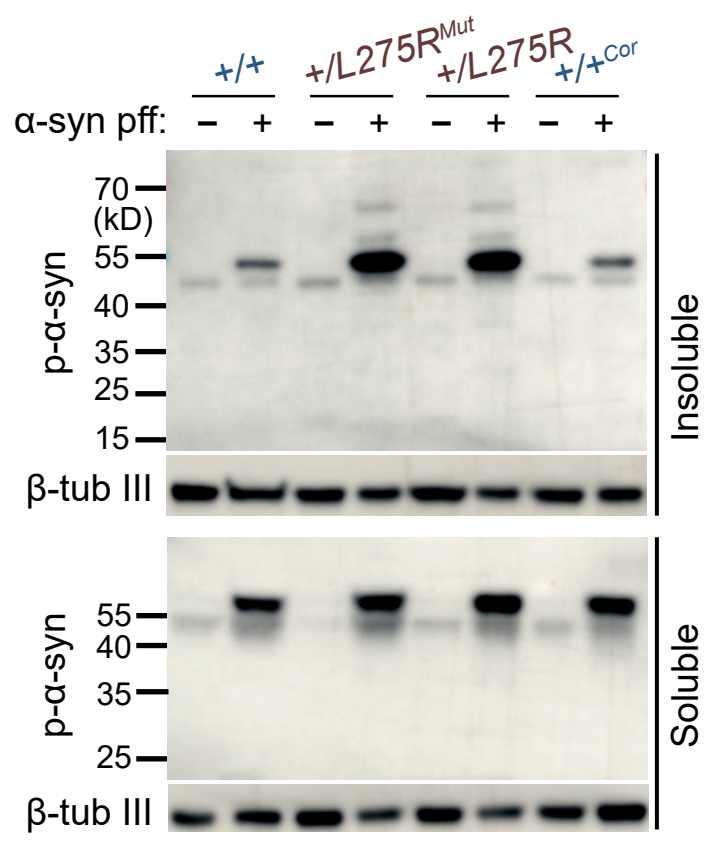

Full unedited blot for Fig. 5D

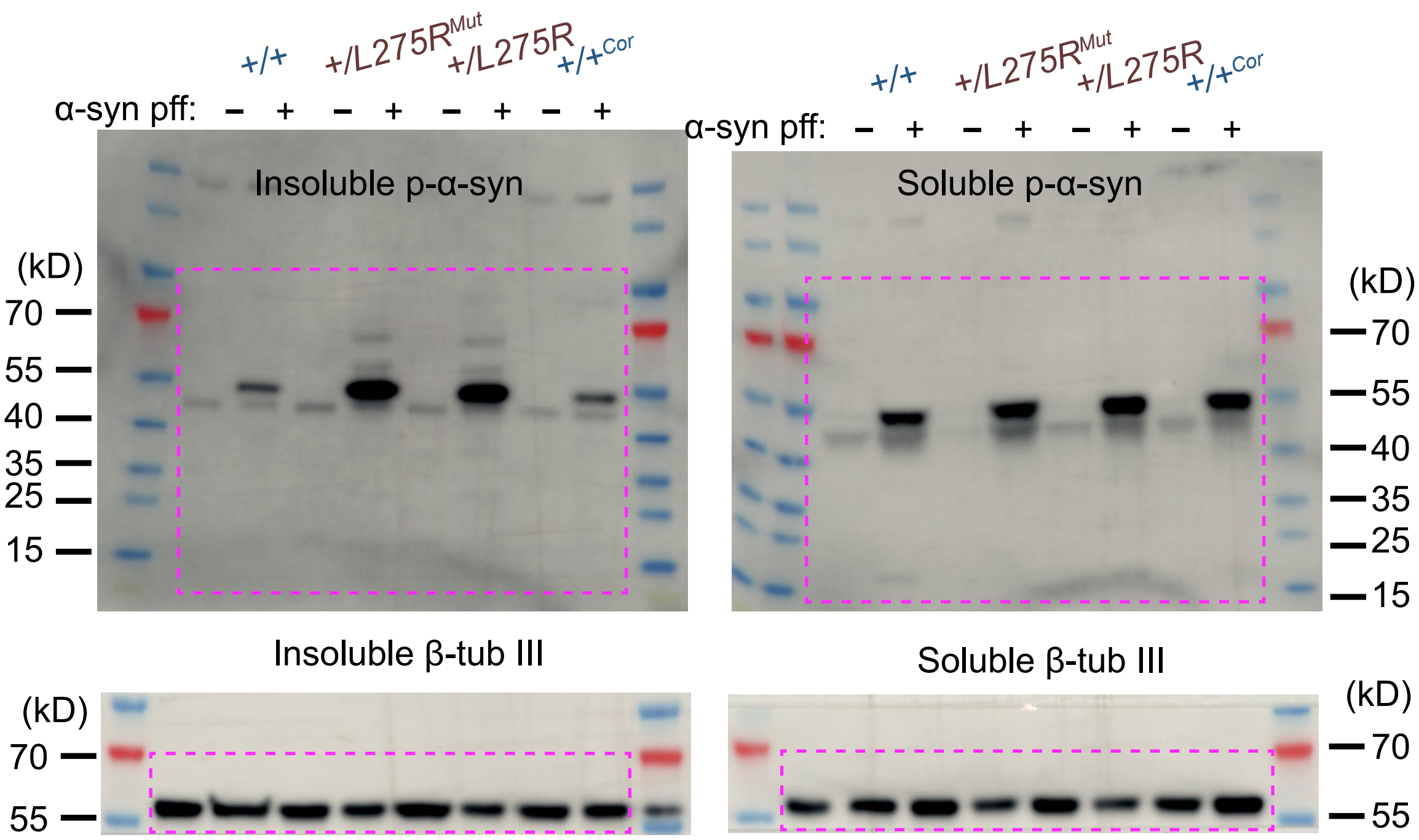

Fig.5E (cropped)

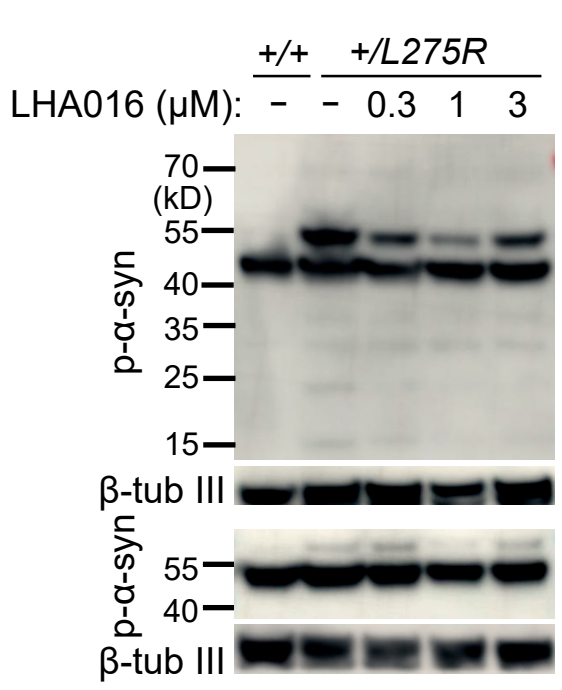

Full unedited blot for Fig. 5E

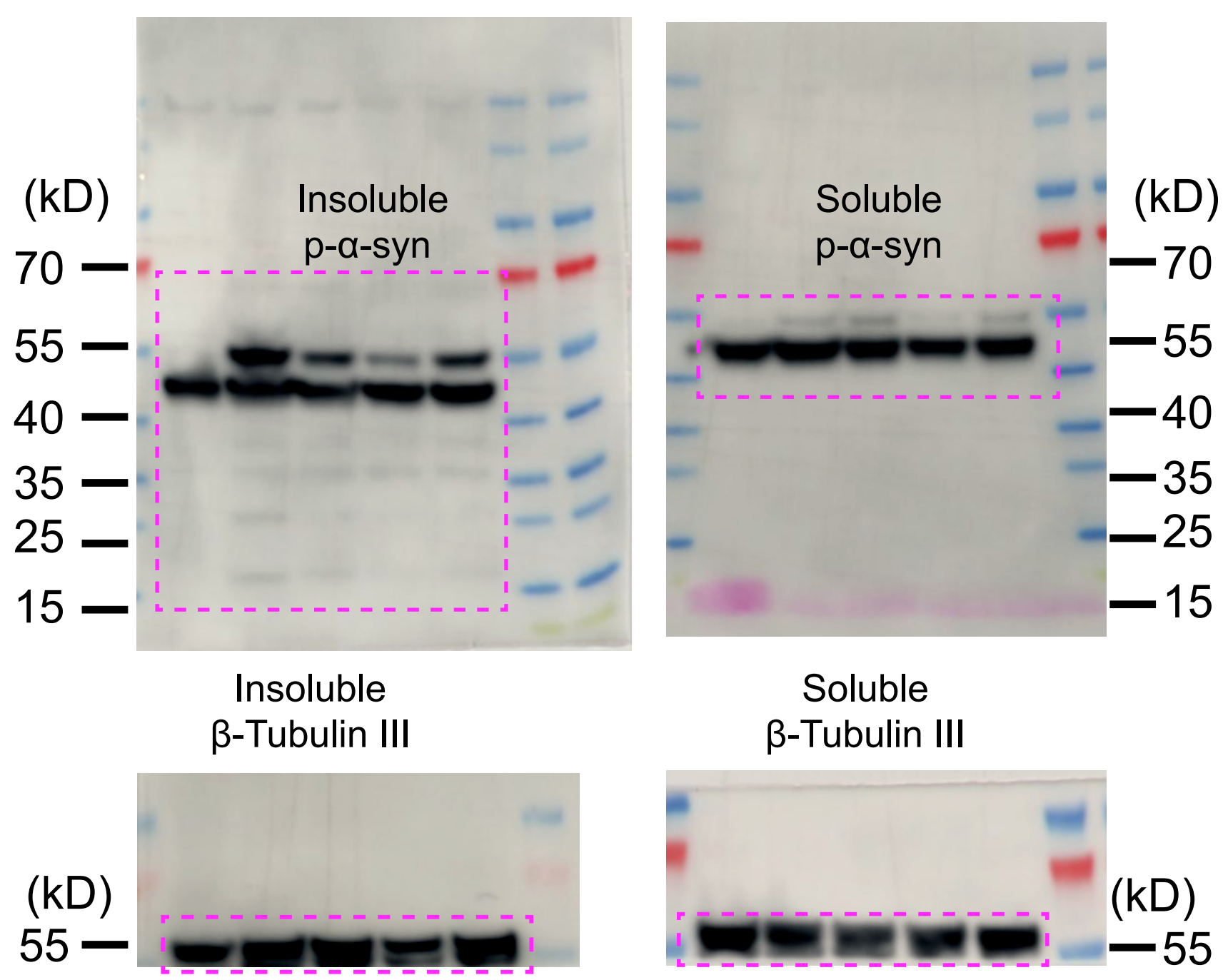

Fig.6I (cropped)

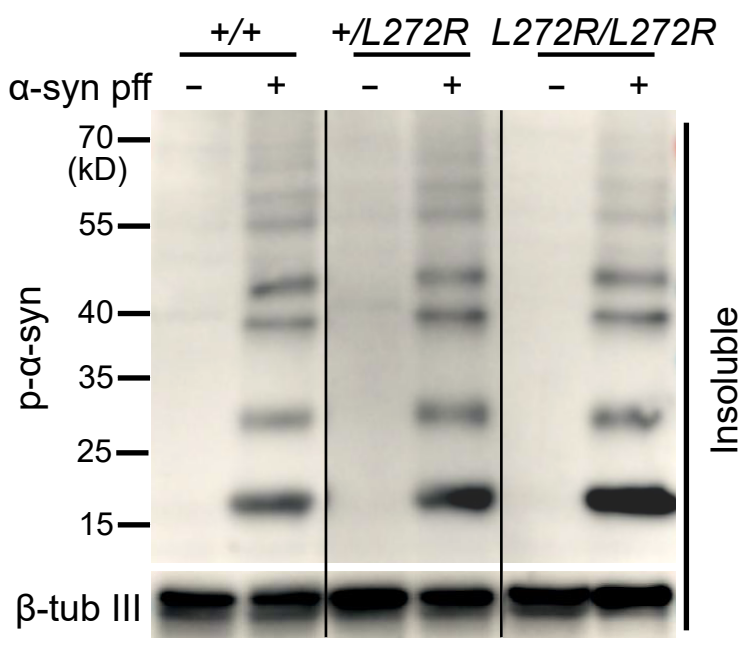

Full unedited blot for Fig. 6I

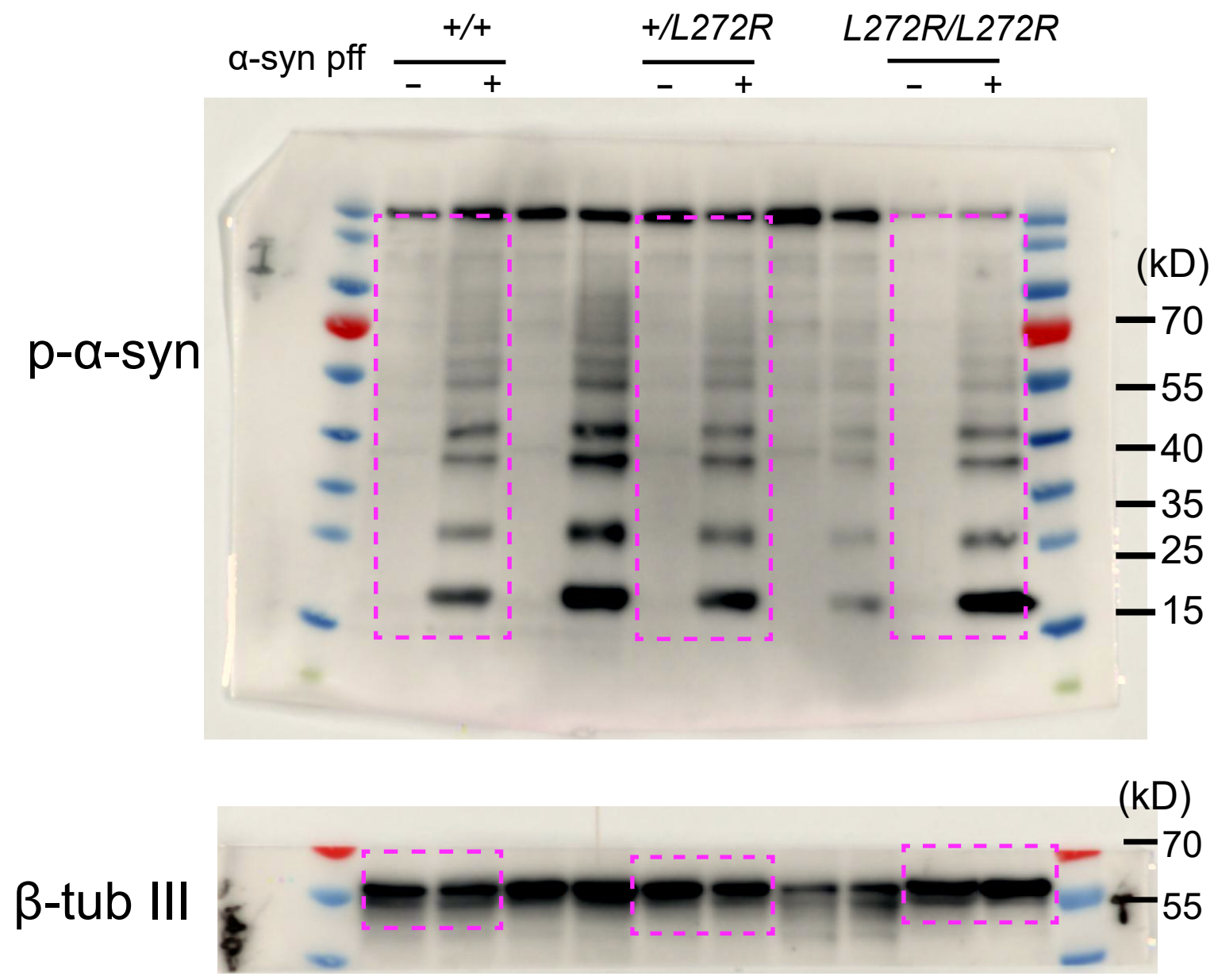

Fig.7C (cropped)

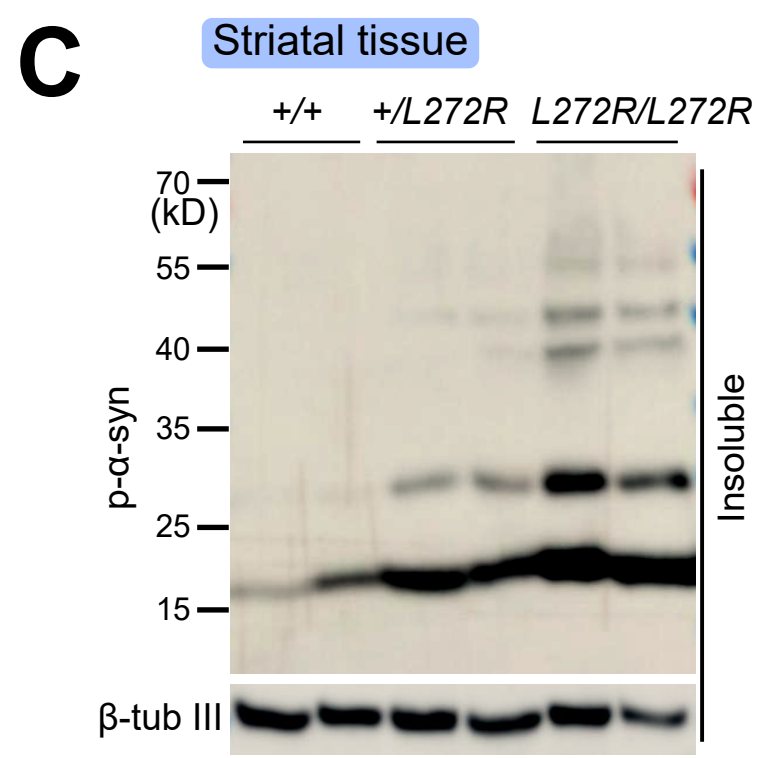

Full unedited blot for Fig. 7C

insoluble p-α-syn

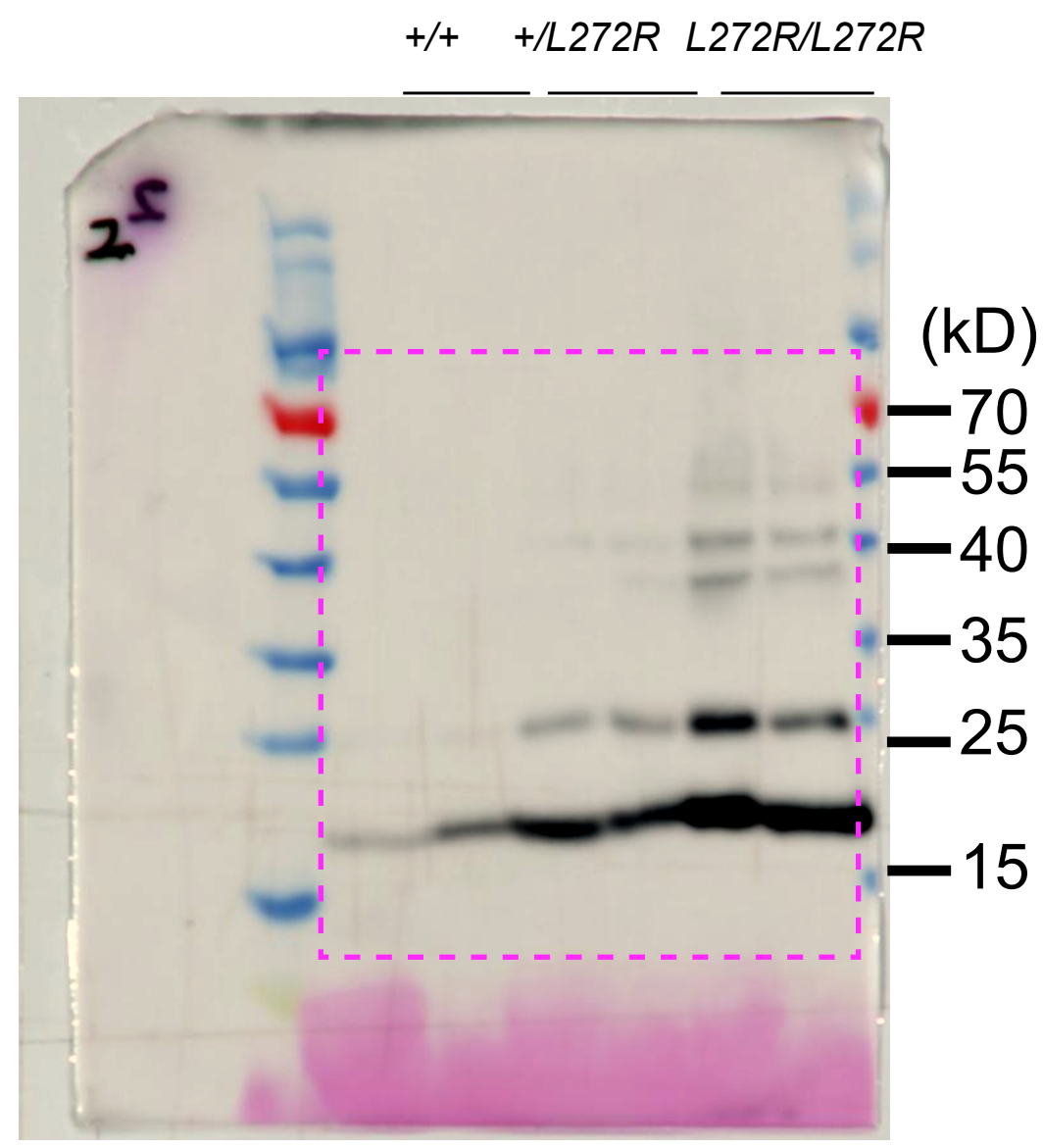

insoluble β-tub III

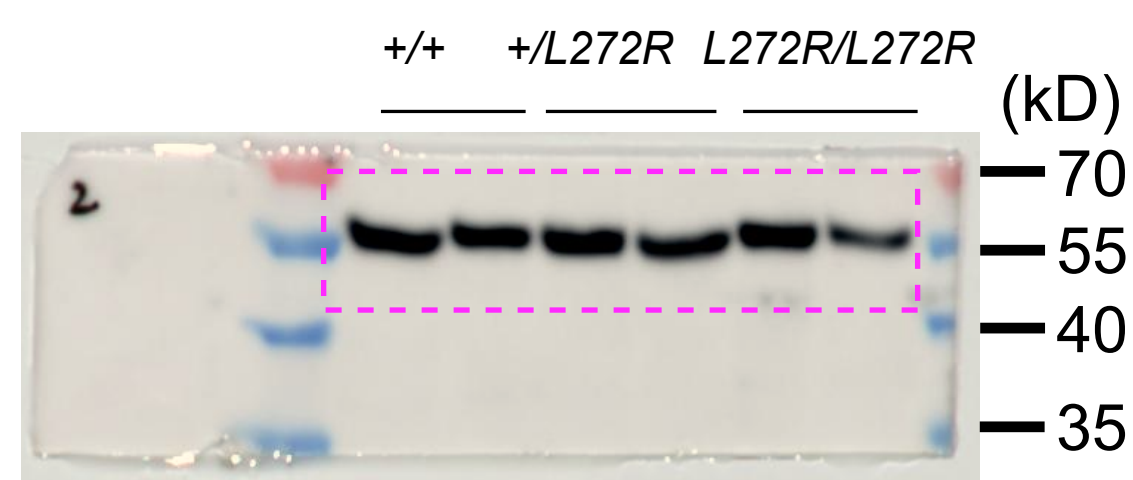

Fig. S1K (cropped)

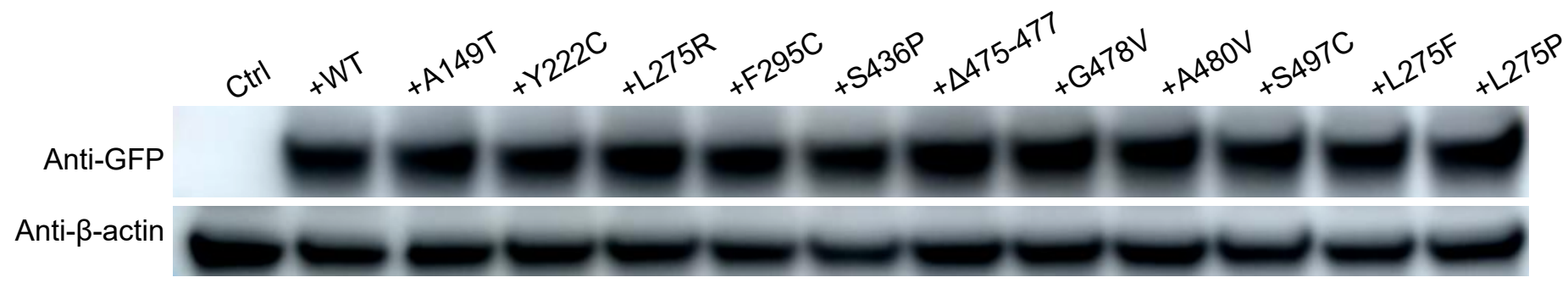

Full unedited blot for Fig. S1K

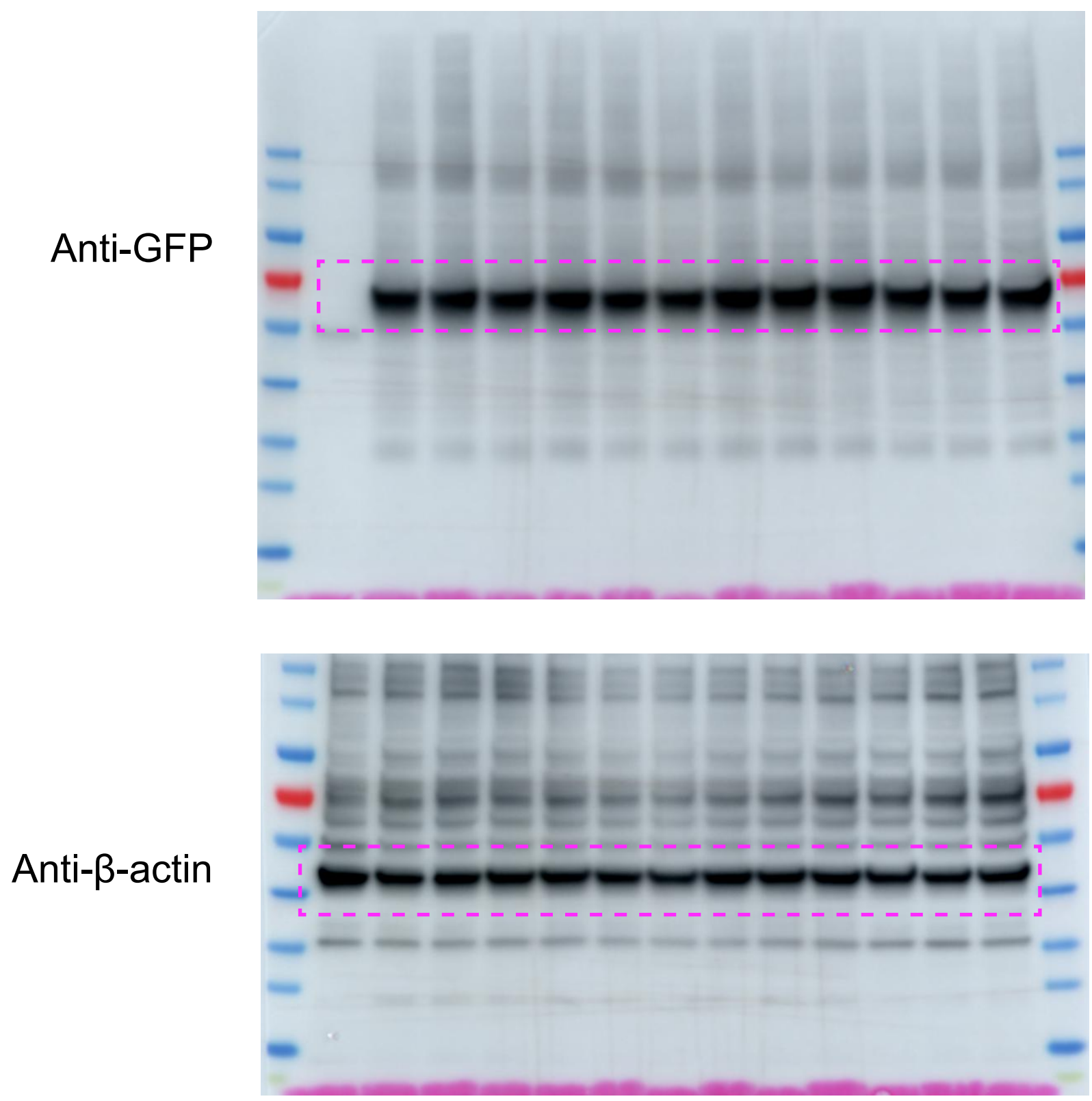

Fig. S3G (cropped)

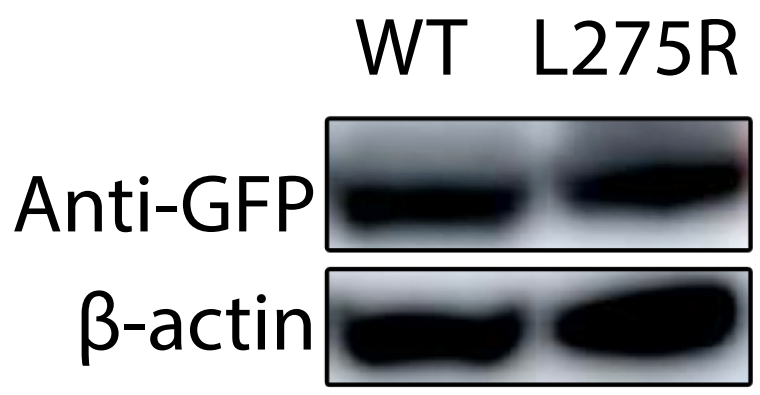

Full unedited blot for Fig. S3G

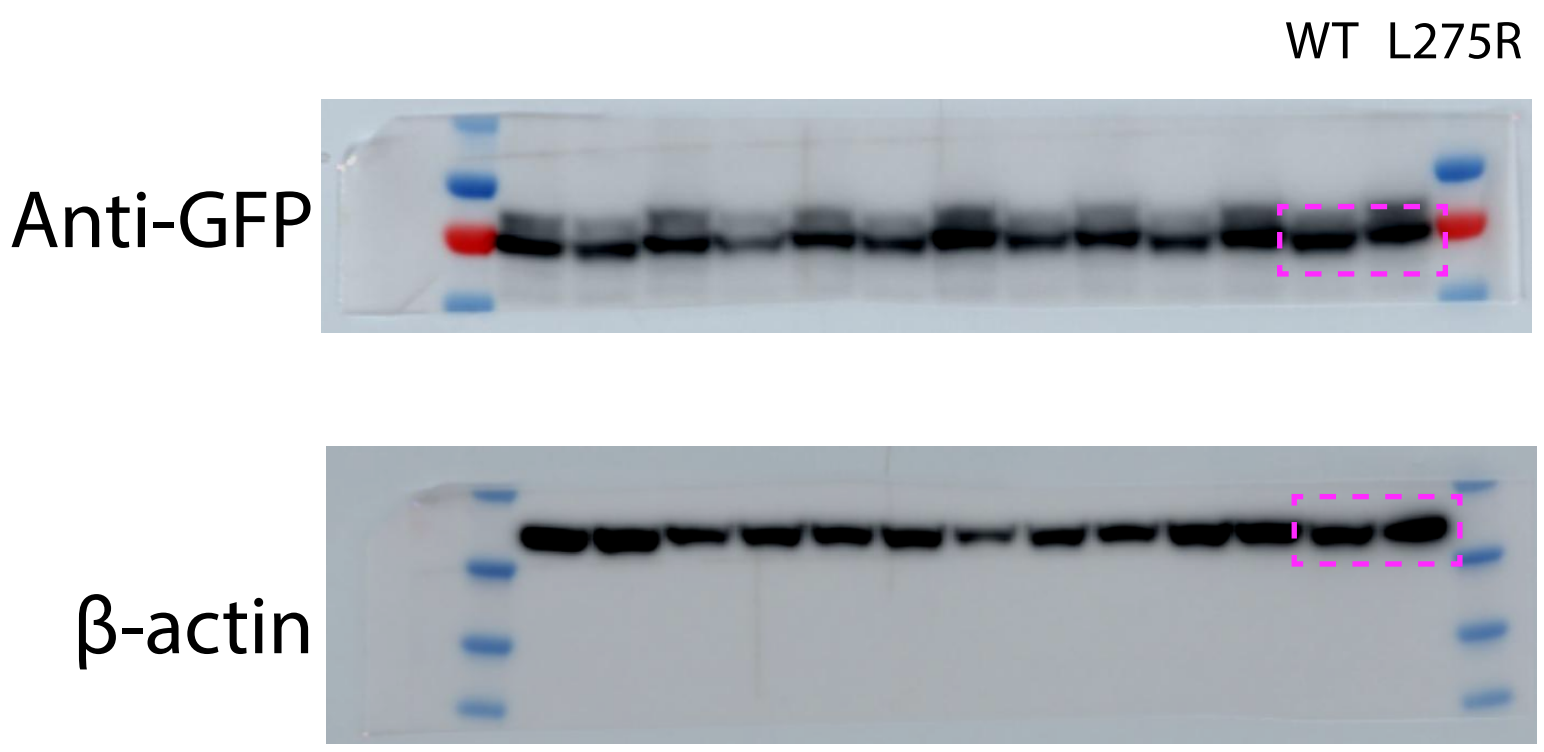

Fig. S7E (cropped)

Full unedited blot for Fig. S7E

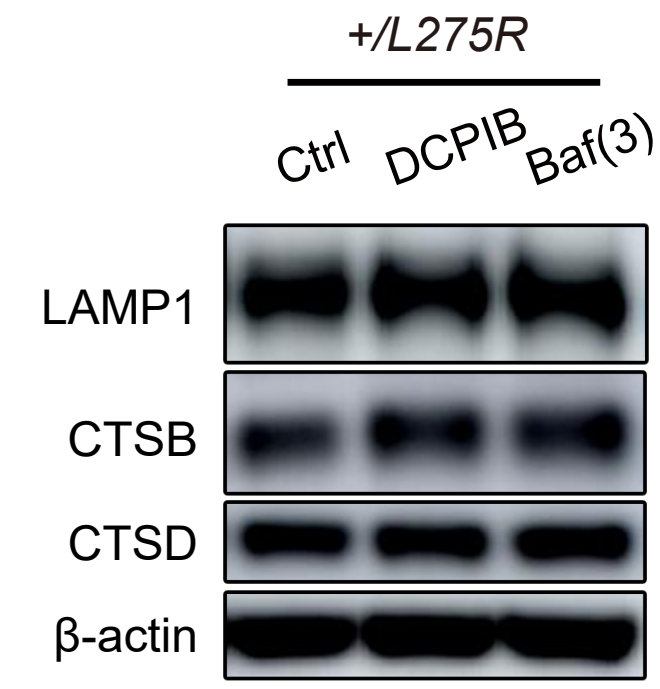

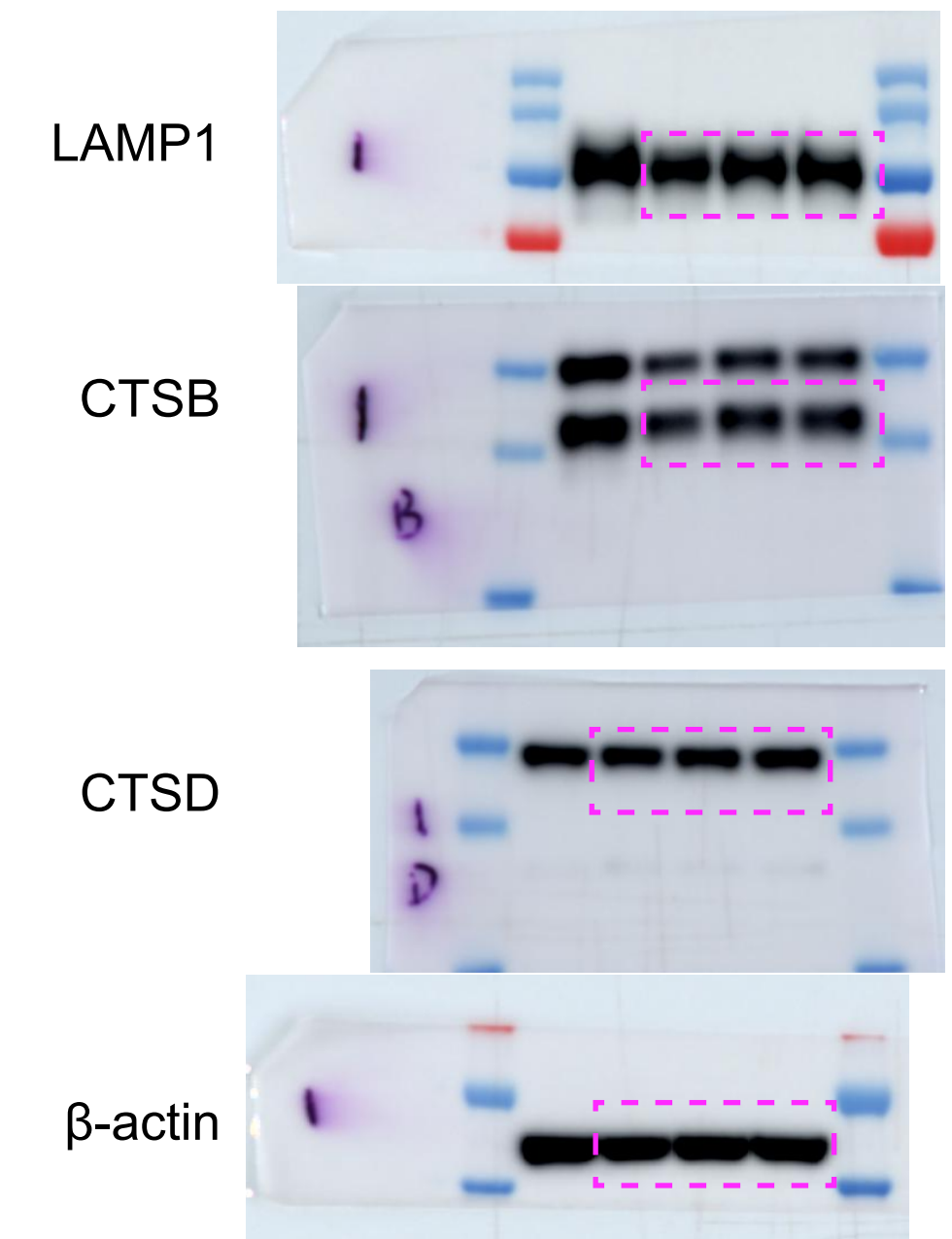

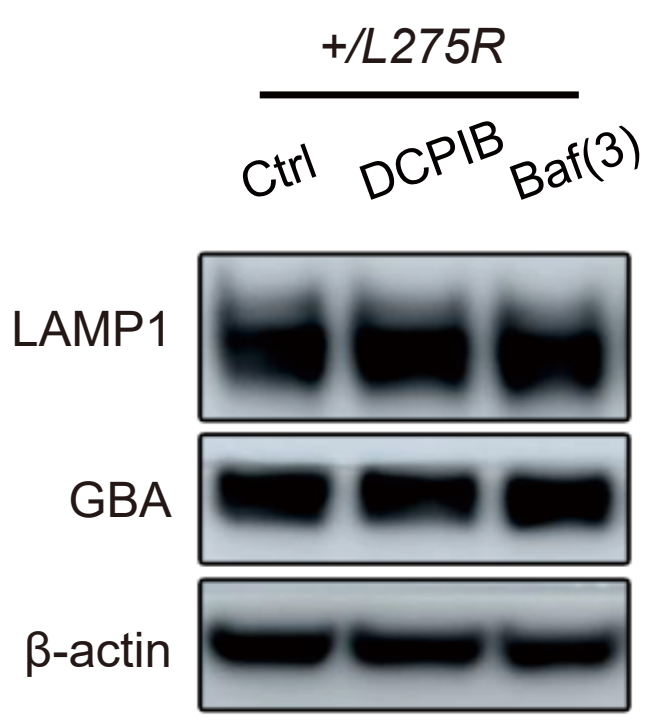

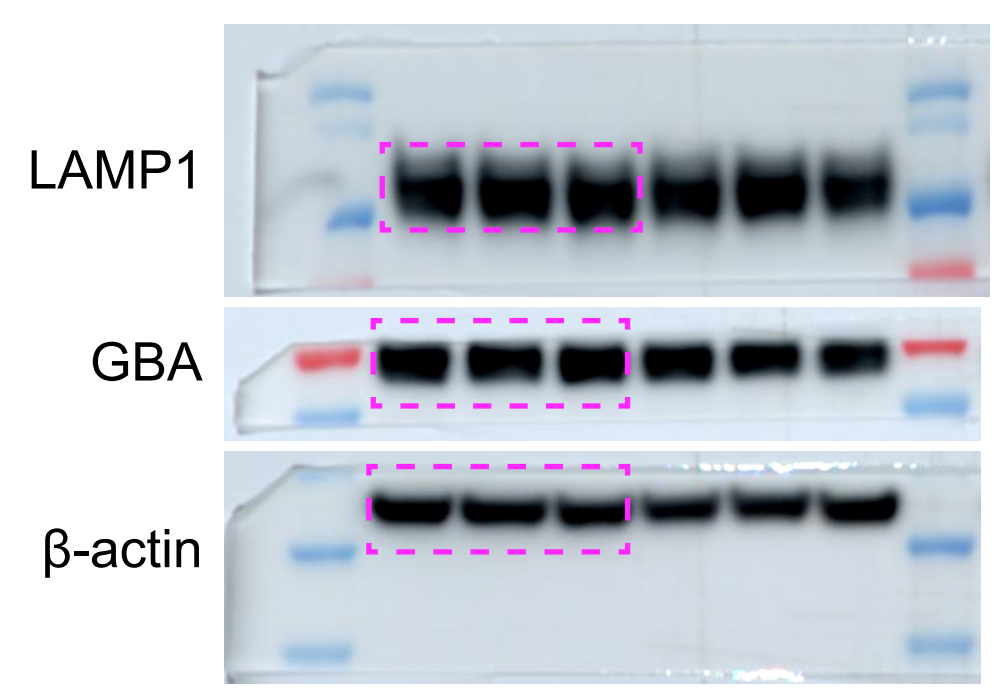

Fig.S8F (cropped)

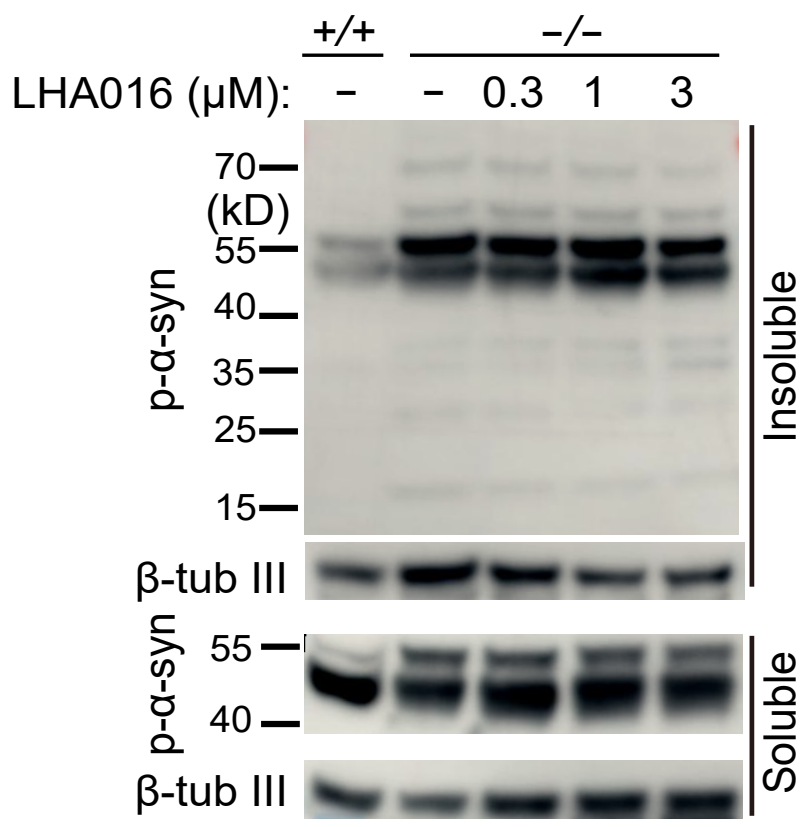

Full unedited blot for Fig. S8F

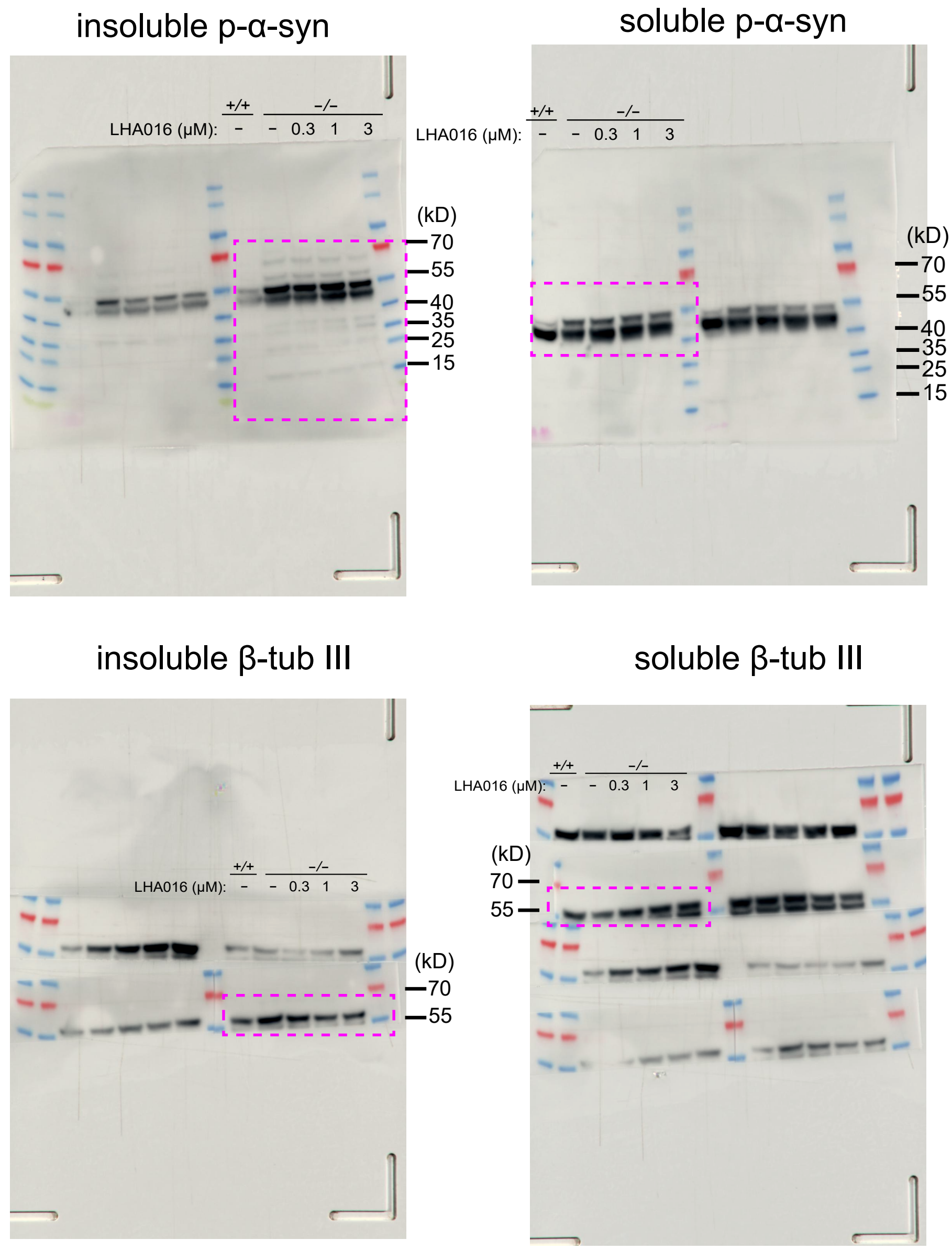

Fig.S10B (cropped)

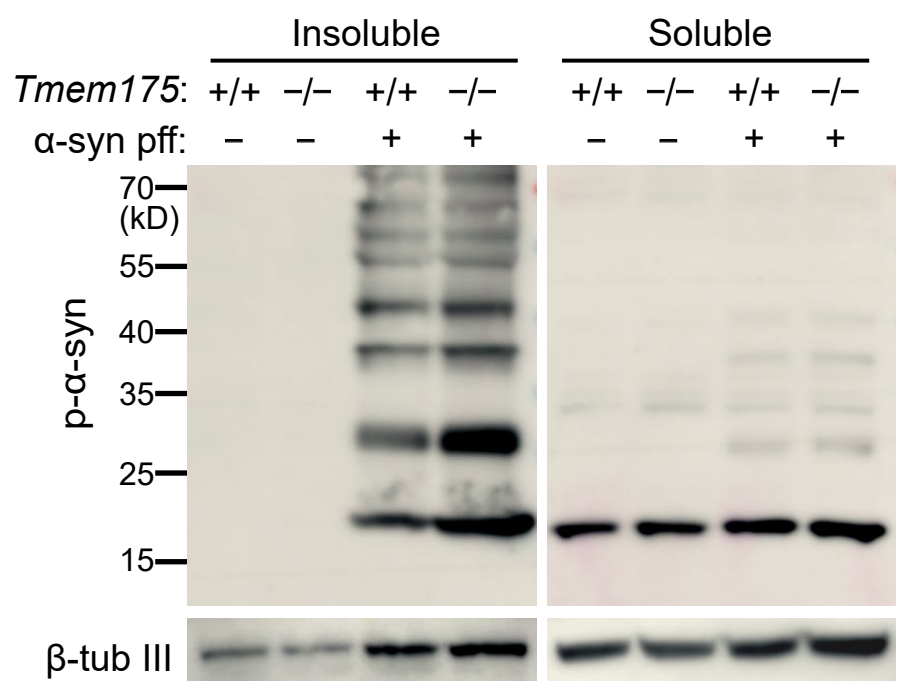

Full unedited blot for Fig. S10B

Insoluble p-α-syn

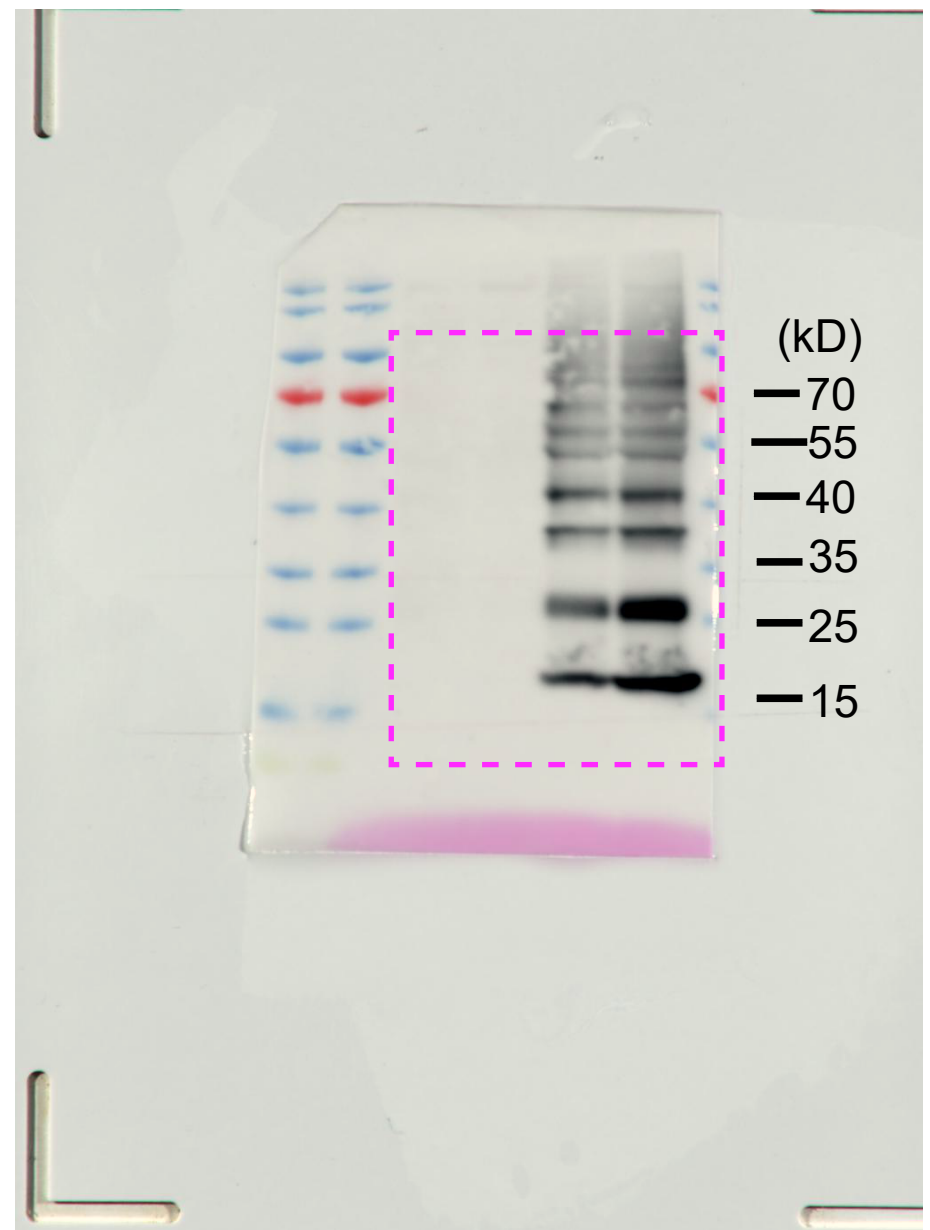

Soluble p-α-syn

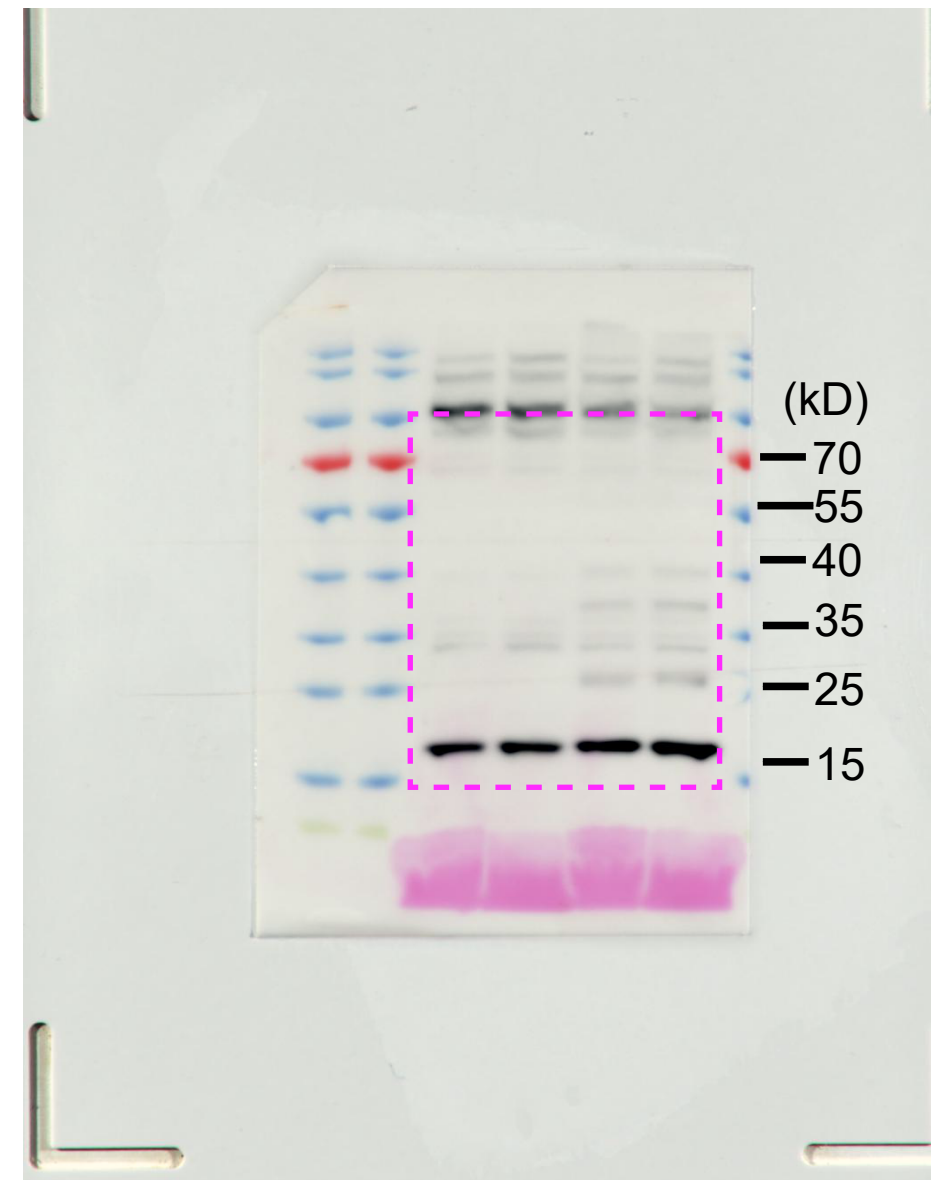

Insoluble β-tub III

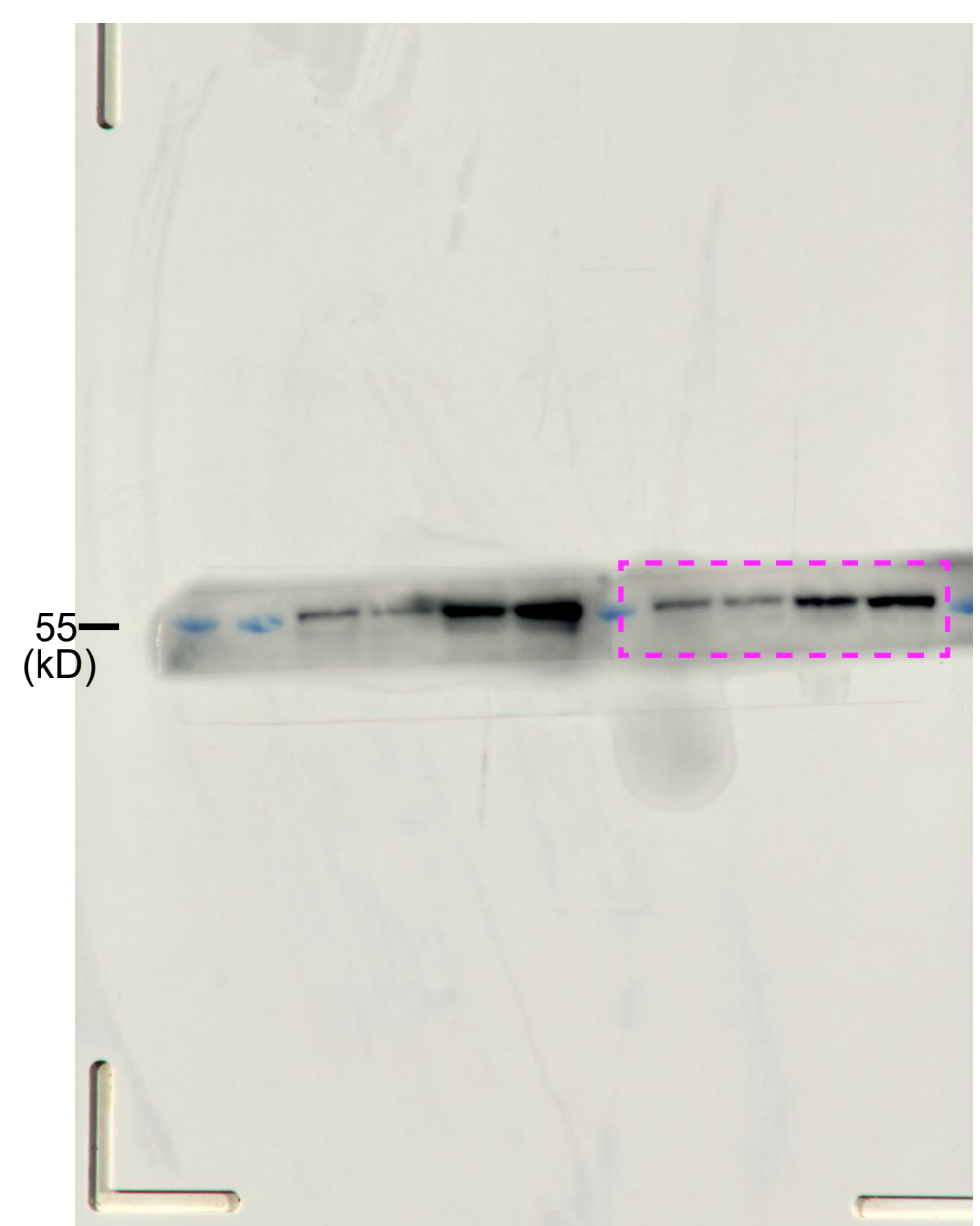

Soluble β-tub III

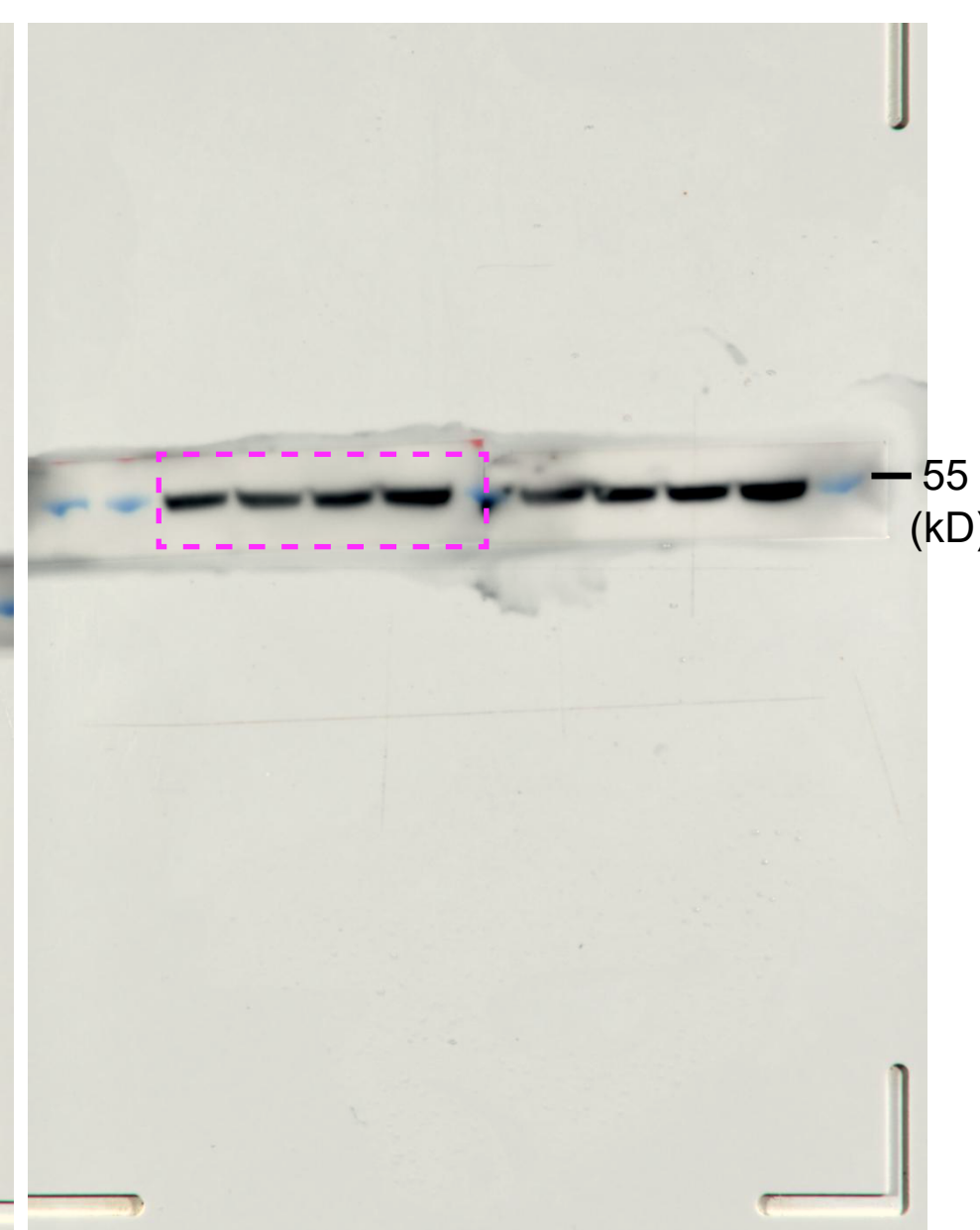

Supplement: Supplementary file 2 [file jci-136-206733-s149.pdf]
